# Association of inflammatory bowel disease with cardiovascular disease, and the mediating role of inflammation: a matched-cohort study

**DOI:** 10.1177/17562848261424145

**Published:** 2026-02-24

**Authors:** Jiawei Geng, Jie Chen, Ola Olén, Jonas Halfvarson, Johan Sundström, Shuai Yuan, Jonas F. Ludvigsson, Jiangwei Sun

**Affiliations:** Department of Big Data in Health Science, School of Public Health, Center of Clinical Big Data and Analytics of The Second Affiliated Hospital, Zhejiang University School of Medicine, Hangzhou, China; Department of Gastroenterology, The Third Xiangya Hospital, Central South University, Changsha, China; Division of Clinical Epidemiology, Department of Medicine Solna, Karolinska Institutet, Stockholm, Sweden; Sachs’ Children and Youth Hospital, Stockholm South General Hospital, Stockholm, Sweden; Department of Gastroenterology, Faculty of Medicine and Health, Örebro University, Örebro, Sweden; Department of Medical Sciences, Uppsala University, Uppsala, Sweden; The George Institute for Global Health, University of New South Wales, Kensington, NSW, Australia; Unit of Cardiovascular and Nutritional Epidemiology, Institute of Environmental Medicine, Karolinska Institutet, Stockholm, Sweden; Department of Surgery, Perelman School of Medicine at the University of Pennsylvania, Philadelphia, PA, USA; Corporal Michael J. Crescenz VA Medical Center, Philadelphia, PA, USA; Department of Pediatrics, Örebro University Hospital, Örebro, Sweden; Division of Digestive and Liver Disease, Department of Medicine, Columbia University Medical Center, New York, NY, USA; Department of Medical Epidemiology and Biostatistics, Karolinska Institutet, Stockholm, Sweden; Department of Medical Epidemiology and Biostatistics, Karolinska Institutet, Nobels väg 12A, Solna 17165, Sweden

**Keywords:** cardiovascular disease, inflammatory, inflammatory bowel disease

## Abstract

**Background:**

The elevated risk of cardiovascular disease (CVD) in inflammatory bowel disease (IBD) has been increasingly recognized. Although inflammation is implicated in both IBD and CVD, the IBD-CVD association through the mediation of systemic inflammation remains underexplored.

**Objective:**

To evaluate the associations between IBD and incident CVD and examine the mediating role of inflammatory biomarkers.

**Design:**

A sex- and age-matched cohort study.

**Methods:**

Using data from the UK Biobank, the study included 1494 participants with a first-ever diagnosis of IBD from 1999 to the recruitment date (2006–2010), and 14,940 matched reference individuals. The primary outcome was any CVD, whereas secondary outcomes included 7 major CVD categories and 19 specific CVD entities. Fifteen inflammatory biomarkers and indices measured at recruitment were included, serving as proxies of underlying inflammatory status. Cox proportional hazard model estimated the adjusted hazard ratios (HRs) and 95% confidence intervals (CI) of associations between IBD and CVD and between inflammatory biomarkers and CVD. Model-based mediation analyses were conducted to explore the potential mediating role of inflammatory biomarkers in IBD-CVD associations.

**Results:**

Compared with reference individuals, patients with IBD had an increased risk of any CVD (HR = 1.34, 95%CI 1.20, 1.49), ischemic heart disease (HR = 1.20, 95%CI 1.02, 1.41), heart failure (HR = 1.61, 95%CI 1.28, 2.02), arrhythmias (HR = 1.34, 95%CI 1.13, 1.59), atrial fibrillation (HR = 1.34, 95%CI 1.11, 1.62), other supraventricular arrhythmia (HR = 1.83, 95%CI 1.12, 2.99), and venous thromboembolism (HR = 1.74, 95%CI 1.38, 2.21). The mediation proportion was generally higher in Crohn’s disease and in CVD subtypes (ischemic heart disease, heart failure, and arrhythmias) than in venous thromboembolism.

**Conclusion:**

IBD is associated with various CVD outcomes, and inflammatory biomarkers mediate their associations, suggesting that reducing inflammation may help alleviate cardiac burden in patients with IBD.

## Plain language summary

Association of inflammatory bowel disease with cardiovascular disease, and the mediating role of inflammation

Based on data from the UK Biobank cohort, we conducted a matched-cohort study. The study observed higher risks of any incident cardiovascular disease (CVD) and its wide range entities, including ischemic heart disease, heart failure, arrhythmias, atrial fibrillation, other supraventricular arrhythmia, and venous thromboembolism. Inflammatory mediation appeared to be higher in Crohn’s disease (CD) than in ulcerative colitis (UC) or IBD-unclassified (IBD-U), with the low-grade inflammation score (INFLA score, a comprehensive composite index assessing inflammation) mediating 14.5%, 10.5%, and 5.3% of the association, respectively. Also, the mediation proportion was generally higher in some CVD subtypes (i.e., ischemic heart disease, heart failure, and arrhythmias) than in venous thromboembolism. The study indicated that IBD was associated with an increased risk of CVD, highlighting the complex pathophysiological process between them. It also suggested that reducing inflammatory level may lower cardiac burden in patients with IBD.

## Introduction

Inflammatory bowel disease (IBD), including Crohn’s disease (CD), ulcerative colitis (UC), and IBD-unclassified (IBD-U), is becoming increasingly common worldwide.^1^ As a complex and incurable disease, its comorbidities pose a great challenge in disease management.^2^ Therefore, early preventive interventions for comorbid conditions may reduce their disease burdens during the chronic course of IBD.^3^

Cardiovascular diseases (CVD) are the world’s leading causes of mortality. Growing evidence suggests an interaction between the digestive and cardiovascular systems via the gut-heart connection.^4–6^ Some previous studies have observed an increased risk of CVD in patients with IBD,^7–13^ while others suggested null associations.^14–16^ The high heterogeneity across individual studies suggested the need for a further comprehensive investigation of the IBD and CVD association.^7^ Although some studies based on UK Biobank data have assessed the associations for specific CVD outcomes, almost all studies have exclusively focused on prevalent IBD, which might introduce bias due to not accounting for disease duration and combining new-onset cases with prevalent ones. The potential role of IBD in other, more specific CVD subtypes, such as arrhythmia and hemorrhagic/ischemic stroke, has yet to be fully explored in the UK Biobank cohort. Moreover, the underlying mechanisms linking IBD to CVD remain unclear. Evidence has suggested that increased inflammatory status in IBD^17^ and a potential mediating role of inflammation on CVD in immune-mediated diseases (e.g., psoriasis and rheumatoid arthritis),^18–20^ which underscores the need to further assess the mediating role of inflammation in the IBD-CVD association.

Therefore, using data from the UK Biobank, we aimed to conduct a matched-cohort study to first assess the associations between IBD and CVD and then explore the mediating effect of inflammatory biomarkers on their associations.

## Materials and methods

### Study population

The UK Biobank is one of the largest prospective population-based cohorts, which recruited more than half a million participants aged between 40 and 69 years old at 22 assessment centers from 2006 to 2010 (recruitment date) across the United Kingdom. At recruitment, blood samples were collected for various key biochemical markers, and participants completed questionnaires, including demographics and lifestyle factors, computer-assisted interviews, and physical and functional assessments.^21^

**Figure 1.**
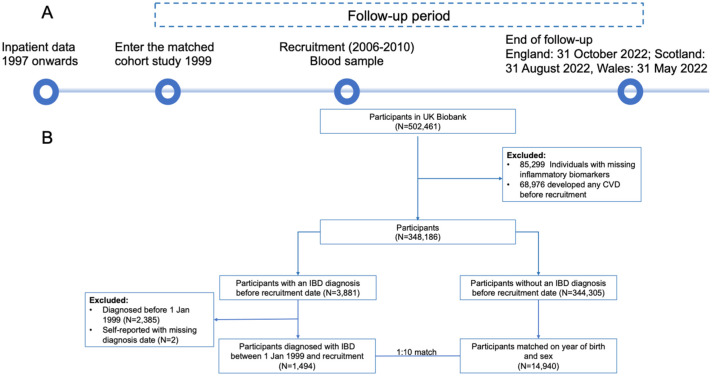
Study flowchart of (a) study period and (b) participant selection. The UK Biobank study recruited participants between 2006 and 2010, during which individuals provided blood samples. Hospital inpatient data have been available since 1997. CVD, cardiovascular disease; IBD, inflammatory bowel disease.

A flowchart of the study period and participant selection is shown in Figure 1. Briefly, among 502,461 participants, to examine the mediating role of inflammatory biomarkers on the IBD-CVD association, we removed individuals with missing inflammatory biomarkers (*N* = 85,299) or those who developed any CVD before entering the UK Biobank study at recruitment (*N* = 68,976), leaving a total of 348,186 participants. Individuals who were diagnosed with IBD before the recruitment date were included (*N* = 3881). However, because hospital linkage was only available from 1997, individuals who had an IBD record between 1997 and 1998 were removed from the analysis to identify patients with a first-ever diagnosis of IBD to minimize the potential impact of including prevalent cases,^22^ which resulted in 1494 cases. Then, for each newly onset IBD, we individually matched up to 10 non-IBD individuals by year of birth (±1 year) and sex (i.e., reference individuals) from the general population.^23^ The index date was defined as the date of diagnosis for IBD patients and the date of being selected for reference individuals.

### Ascertainment of IBD

Patients with IBD and subtypes were ascertained via inpatient data, primary care data, and self-report information in a verbal interview at recruitment. International Classification of Diseases (ICD)-10 and ICD-9 were used to identify IBD (Table S1 and Figure S1). The diagnostic accuracy of IBD has a positive predictive value of 87%–92%.^24,25^ Individuals with a record of both CD and UC at recruitment were classified as IBD-U.

### Ascertainment of CVD

The primary outcome is newly onset CVD after the recruitment date, which was identified via data from inpatient, primary care, self-report, and death registries using ICD-9 and ICD-10 (Table S2). Secondary outcomes included seven major categories of CVD: (1) ischemic heart disease (myocardial infarction); (2) cerebrovascular disease (including stroke, hemorrhagic stroke, and ischemic stroke); (3) myocarditis; (4) heart failure; (5) arrhythmias (including atrial fibrillation, bradyarrhythmia, other supraventricular arrhythmias, and cardiac arrest and ventricular arrhythmias); (6) peripheral artery disease; and (7) venous thromboembolism (including pulmonary embolism and deep vein thrombosis).

### Measurement of inflammatory biomarkers

Biomarkers were chosen from the UK Biobank Biomarker Project, which were extracted from peripheral blood samples at recruitment. A total of 15 potential inflammatory biomarkers or indices were included (definitions in Table S3) based on data availability and evidence from previous investigations.^26^ These inflammatory biomarkers included albumin, C-reactive protein (CRP), lymphocyte, platelet, and white blood cells (including eosinophil, monocyte, and neutrophil). In addition, seven composite indices were assessed, including lymphocyte-to-monocyte ratio (LMR), neutrophil-to-lymphocyte ratio (NLR), platelet-to-lymphocyte ratio (PLR), CRP-to-albumin ratio (CAR), systemic immune-inflammatory index (SII), systemic inflammatory response index (SIRI), and low-grade inflammation score (INFLA score). Composite indices were proposed as more reliable long-term indicators than individual biomarkers, with the INFLA score providing the most comprehensive evaluation of the body’s inflammation response.^27^ As described, the INFLA score was calculated by using deciles of four biomarkers (CRP, white blood cell count, platelet count, and the NLR). For each of the biomarkers, the highest four deciles (7th to 10th) were assigned 1–4 values, the lowest deciles were assigned from −4 to −1 (1st to 4th), and the remaining were assigned as 0. The sum of the values across the four biomarkers is the INFLA score, ranging from −16 to 16.

### Assessment of covariates

The following covariates were considered^9,10^: age at index date (continuous), sex (male/female), Townsend deprivation index (TDI; low, middle, and high deprivation; categorized by tertiles), ethnicity (white/non-white), education attainment (below/above college), body mass index (BMI <25 kg/m^2^, 25 ⩽ BMI < 30 kg/m^2^, and ⩾30 kg/m^2^), physical activity (regular/irregular), smoking status (never, previous, and current smokers), drinking status (never, previous, and current drinkers), history of diabetes (yes/no), hypertension (yes/no), and hyperlipemia (yes/no) at index date, and adherence to healthy diet (i.e., cardioprotective diet; healthy or not). A directed acyclic graph was used to visualize their relationships (Figure S2). A cardioprotective diet, based on American Heart Association recommendations, specifies the optimal dietary intake for cardiometabolic health. Considering the potential influence of IBD-related medications,^28^ we additionally adjusted for medication history in the model, including 5-aminosalicylates, biologic drugs (note that only adalimumab was recorded), immunosuppressants, and glucocorticoids. Medication use was collected from baseline touch-screen questionnaires and verbal interviews (yes/no).^29,30^ All missing data of covariates were classified into a separate classification coded as “Unknown.” The detailed definition of each covariate can be found in Table S4.

### Statistical analysis

Characteristics of participants on index date were described as median (interquartile range (IQR)) for continuous variables and number (percentage) for categorical variables, stratifying by patients with IBD and reference individuals. Kruskal–Wallis and Chi-square tests were used to compare the differences between groups. Spearman correlation test was used to examine the associations between 15 biomarkers or indices.

### IBD-CVD analysis

Cox proportional hazard (PH) model was used to calculate the hazard ratios (HRs) of CVD outcomes with 95% confidence intervals (CI). We tested the PH assumption through Schoenfeld residuals. In cases where the PH assumption is violated, HR derived from the proportional Cox regression model can be interpreted as a weighted average effect during the follow-up period. In Model 1, we adjusted for age, sex, TDI, ethnicity, and education, and in Model 2, we further adjusted for BMI, alcohol drinking, smoking status, physical activity, history of hypertension, diabetes, hyperlipidemia at index date, and adherence to cardioprotective diet. All participants were followed from the index date and ended at CVD diagnosis, death, loss to follow-up, or the end of follow-up (October 31, 2022 for England, August 31, 2022 for Scotland, and May 31, 2022 for Wales), whichever came first. Reference individuals were censored if being diagnosed with IBD during the follow-up period. Incidence rate (IR; per 1000 person-years) with 95%CI was calculated.

### IBD-biomarkers analysis

To investigate the association of IBD and every 15 inflammatory biomarkers, we applied linear-mixed effect models. In each model, IBD and covariates were considered as fixed effects, while the UK Biobank assessment center was regarded as a random effect. Inflammatory markers were standardized to *z*-scores when the model was fitted. Multiple comparisons were adjusted by using the Bonferroni correction. The adjustments were the same as the ones that were applied in the fully adjusted model in the IBD-CVD analysis.

### Biomarker-CVD analysis

We restricted this analysis to CVD outcomes that had at least one significant association with IBD in the IBD-CVD analysis. Cox PH model was applied to assess the linear and nonlinear associations between inflammatory biomarkers and CVD. In the linear analysis, we treated biomarkers as continuous variables (per standard deviation (SD)) for each inflammatory biomarker, while in the nonlinear analysis, we used the restricted cubic spline function, in which the reference value (i.e., HR = 1) was set at the 50th percentile and the knots were set at the 10th, 50th, and 90th percentiles of the distribution of the biomarkers.

### Mediation analysis

To investigate if any of the IBD-CVD associations were mediated by inflammatory markers, we performed the mediation analysis by using the “Mediation” R package.^31^ Only CVD outcomes that reached statistical significance in the IBD-CVD analysis were included. To decompose the total effect of exposure on the outcomes (i.e., the sum of direct effect and indirect effect) into direct (i.e., the effect of exposure on outcome independent of the mediator) and indirect effect (i.e., the effect of exposure on the outcome through a mediator), a standard three-variable path model was constructed. We first fitted the association between IBD and inflammatory biomarkers using a linear regression model and then fitted survival regression models for the association between both IBD-CVD and biomarkers-CVD associations.^32^ By combining these models, the direct effect, the indirect effect, and the total effect in the IBD and CVD association could be estimated.^31^ The mediation proportion was calculated by dividing the indirect effect by the total effect, representing the proportion of the association between IBD and CVD that can be explained by specific inflammatory biomarkers. The significance of mediating effects was determined by 1000 quasi-Bayesian approximation iterations. All fitted models were adjusted using the same covariates described above in the IBD-CVD analysis. In the analysis by IBD subtypes, CVD outcomes with fewer than 100 events were not analyzed due to power issues, but were included in analyses for the relevant main categories.

### Subgroup and sensitivity analyses

Subgroup analyses were conducted to detect the potential effect modification. The *p*-interaction was calculated by including the multiplication interaction term of IBD with age at index date (⩽50 years, >50 years), sex (male, female), and the time interval between index date to recruitment date (⩽5 years, >5 years; to explore whether the time from IBD diagnosis to covariates assessed at recruitment influenced the results) in the Cox PH model using the Wald test. In sensitivity analyses, we also additionally adjusted for family history of CVD (defined as parental stroke or heart disease),^9^ Charlson comorbidity index (CCI, as a continuous variable, a proxy for general health status), and metabolic health (healthy/unhealthy) to further assess their potential influence on the IBD and CVD association. Second, participants with self-reported IBD were removed from the analysis to further assess the associations between IBD and CVD outcomes. In addition, we conducted a sensitivity analysis where patients with IBD-U were identified as those diagnosed with both UC and CD during the whole study period (a stringent definition, *n* = 308). For pairs that did not meet the PH assumption (CD-any CVD, IBD-U-any CVD, IBD-heart failure, IBD-U-heart failure, and CD-Peripheral artery disease), we employed a step function by dividing the follow-up period into arbitrary intervals: 0–5 years, 5–10 years, and more than 10 years as supplement.^10^

All statistical analyses were conducted using R software (version 4.2.1, R Core Team, Vienna, Austria), and a two-tailed *p* < 0.05 was considered statistically significant. This study is reported as per the Strengthening the Reporting of Observational Studies in Epidemiology (STROBE) guideline (see Supplemental Data).

## Results

### Characteristics of study participants

A total of 1494 newly onset IBD patients (331 CD, 964 UC, and 199 IBD-U) and 14,940 reference individuals were included in the analysis, with a median follow-up of around 17.5 years. The median age of participants at the index date was 52 years, and 50.7% were females. Compared with the reference individuals, IBD patients had a higher socioeconomic deprivation, a higher rate of smoking history, lower education attainment, were more likely to suffer from comorbidities, and engaged in physical activity less regularly, and were less likely to adhere to a cardioprotective healthy diet at recruitment. Statistical differences were observed for almost all inflammatory biomarkers between IBD patients and their matched reference individuals (Table 1).

**Table 1.** Characteristics of patients with IBD and their matched reference individuals.

Characteristics	Reference individuals (*N* = 14,940)	IBD patients (*N* = 1494)	*p*
Age at index date, median (IQR)	52 (44, 59)	52 (44, 58)	0.997
Female (%)	7570 (50.7)	757 (50.7)	1
Body mass index (%)			
<25 kg/m^2^	5056 (33.8)	511 (34.2)	0.946
25–30 kg/m^2^	6364 (42.6)	626 (41.9)	
⩾30 kg/m^2^	3463 (23.2)	352 (23.6)	
Unknown	57 (0.4)	5 (0.3)	
Townsend deprivation index (%)			0.001
Low deprivation	5070 (33.9)	440 (29.5)	
Moderate deprivation	5046 (33.8)	512 (34.3)	
High deprivation	4808 (32.2)	542 (36.3)	
Unknown	16 (0.1)	0 (0.0)	
Ethnicity			0.771
Non-White	804 (5.4)	74 (5.0)	
White	14,072 (94.2)	1414 (94.6)	
Unknown	64 (0.4)	6 (0.4)	
Education attainment			<0.001
Below college	9712 (65.0)	1045 (69.9)	
College degree and above	5066 (33.9)	429 (28.7)	
Unknown	162 (1.1)	20 (1.3)	
Alcohol drinking (%)			<0.001
Never	652 (4.4)	76 (5.1)	
Previous	445 (3.0)	74 (5.0)	
Current	13,810 (92.4)	1340 (89.7)	
Unknown	33 (0.2)	4 (0.3)	
Smoking (%)			<0.001
Never	8341 (55.8)	664 (44.4)	
Previous	4971 (33.3)	666 (44.6)	
Current	1554 (10.4)	160 (10.7)	
Unknown	74 (0.5)	4 (0.3)	
Physical activity (%)			0.003
Irregular	4327 (29.0)	488 (32.7)	
Regular	10,613 (71.0)	1006 (67.3)	
Adherence to a cardioprotective healthy diet (%)			0.003
No	5035 (33.7)	569 (38.1)	
Yes	9889 (66.2)	924 (61.8)	
Unknown	16 (0.1)	1 (0.1)	
Hypertension (%)	2162 (14.5)	223 (14.9)	0.662
Diabetes (%)	328 (2.2)	43 (2.9)	0.109
Hyperlipidemia (%)	814 (5.4)	78 (5.2)	0.756
Charlson comorbidity index			<0.001
0	14,144 (94.7)	1334 (89.3)	
⩾1	796 (5.3)	160 (10.7)	
Charlson comorbidity index, median (IQR)	0.00 (0.00, 0.00)	0.00 (0.00, 0.00)	<0.001
Charlson comorbidity index, mean (SD)	0.10 (0.54)	0.19 (0.67)	<0.001
Metabolic status			0.631
Healthy	8255 (55.3)	819 (54.8)	
Unhealthy	6675 (44.7)	673 (45.0)	
Unknown	10 (0.1)	2 (0.1)	
5-aminosalicylates user (%)	32 (0.2)	579 (38.8)	<0.001
Biological drugs user (%)	4 (0.0)	2 (0.1)	0.175
Immunosuppressants user (%)	89 (0.6)	176 (11.8)	<0.001
Glucocorticoids user (%)	120 (0.8)	102 (6.8)	<0.001
IBD			<0.001
CD	0 (0.0)	331 (22.2)	
UC	0 (0.0)	964 (64.5)	
IBD-U	0 (0.0)	199 (13.3)	
Inflammatory biomarkers			
Albumin, median (IQR)	45.31 (43.58, 46.99)	44.66 (42.90, 46.54)	<0.001
CRP, median (IQR)	1.28 (0.64, 2.61)	1.85 (0.85, 4.12)	<0.001
Lymphocyte count, median (IQR)	1.87 (1.50, 2.29)	1.76 (1.40, 2.21)	<0.001
Platelet count, median (IQR)	247.80 (213.40, 286.00)	259.10 (220.43, 304.00)	<0.001
Eosinophil count, median (IQR)	0.13 (0.10, 0.20)	0.14 (0.10, 0.22)	0.169
Monocyte count, median (IQR)	0.45 (0.36, 0.57)	0.46 (0.36, 0.59)	0.185
Neutrophil count, median (IQR)	4.00 (3.23, 4.90)	4.30 (3.40, 5.40)	<0.001
White blood cell count, median (IQR)	6.60 (5.60, 7.79)	6.88 (5.70, 8.29)	<0.001
LMR, median (IQR)	4.20 (3.28, 5.33)	3.93 (2.95, 5.16)	<0.001
NLR, median (IQR)	2.14 (1.67, 2.76)	2.45 (1.81, 3.30)	<0.001
PLR, median (IQR)	132.27 (105.82, 167.06)	146.42 (114.80, 192.07)	<0.001
CAR, median (IQR)	0.03 (0.01, 0.06)	0.04 (0.02, 0.09)	<0.001
SII, median (IQR)	526.80 (390.67, 710.46)	626.54 (443.44, 909.47)	<0.001
SIRI, median (IQR)	0.94 (0.67, 1.34)	1.10 (0.75, 1.70)	<0.001
INFLA score, median (IQR)	0.00 (−5.00, 4.00)	2.00 (−3.00, 7.00)	<0.001
Incident CVD			
Any CVD (%)	2960 (19.8)	391 (26.2)	<0.001
Ischemic heart disease (%)	1325 (8.9)	163 (10.9)	0.010
Myocardial infarction (%)	379 (2.5)	43 (2.9)	0.478
Cerebrovascular disease (%)	599 (4.0)	68 (4.6)	0.345
Stroke (%)	355 (2.4)	40 (2.7)	0.525
Hemorrhagic stroke (%)	96 (0.6)	11 (0.7)	0.794
Ischemic stroke (%)	245 (1.6)	26 (1.7)	0.854
Myocarditis (%)	101 (0.7)	12 (0.8)	0.687
Heart failure (%)	511 (3.4)	86 (5.8)	<0.001
Arrhythmias (%)	1146 (7.7)	156 (10.4)	<0.001
Atrial fibrillation (%)	908 (6.1)	124 (8.3)	0.001
Bradyarrhythmia (%)	136 (0.9)	19 (1.3)	0.216
Other supraventricular arrhythmias (%)	105 (0.7)	19 (1.3)	0.023
Cardiac arrest and ventricular arrhythmias (%)	168 (1.1)	19 (1.3)	0.701
Peripheral artery disease (%)	157 (1.1)	20 (1.3)	0.370
Venous thromboembolism (%)	467 (3.1)	82 (5.5)	<0.001
Pulmonary embolism (%)	253 (1.7)	29 (1.9)	0.550
Deep vein thrombosis (%)	259 (1.7)	59 (3.9)	<0.001

CAR, C-reactive protein to albumin ratio; CD, Crohn’s disease; CRP, C-reactive protein; CVD, cardiovascular disease; IBD, inflammatory bowel disease; IBD-U, IBD-unclassified; IQR, interquartile range, the 25th percentile to the 75th percentile; LMR, lymphocyte-to-monocyte ratio; PLR, platelet-to-lymphocyte ratio; NLR, neutrophil-to-lymphocyte ratio; SD, standard deviation; SII, systemic immune-inflammatory index; SIRI, systemic inflammation response index; UC, ulcerative colitis.

### Associations between IBD, inflammatory biomarkers, and CVD

**Figure 2.**
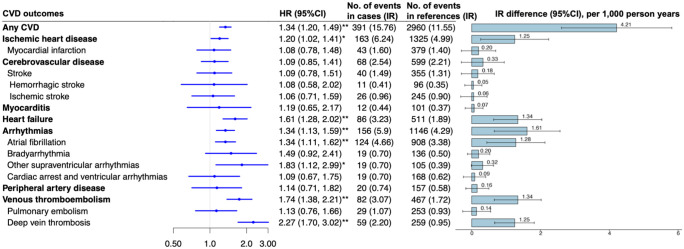
Incident CVD in patients with IBD compared with their matched reference individuals. The HR and 95% CI were estimated from the Cox regression model by adjusting for age, sex, Townsend deprivation index, ethnicity, education, body mass index, alcohol drinking, smoking status, physical activity, hypertension, diabetes, hyperlipidemia, and adherence to cardioprotective diet. The association with Bonferroni corrected *p* values<0.05 was labeled as **, while Bonferroni corrected *p* ⩾ 0.05 and *p* < 0.05 were labeled as *. CI, confidence intervals; CVD, cardiovascular disease; HR, hazard ratio; IBD, inflammatory bowel disease; IR, incidence rate.

In model 1, IBD was associated with eight of the 18 CVD outcomes, and all remained statistically significant even in model 2 (Table S5). The HRs were 1.34 (95%CI 1.20, 1.49) for any CVD, 1.20 (95%CI 1.02, 1.41) for ischemic heart disease, 1.61 (95%CI 1.28, 2.02) for heart failure, 1.34 (95%CI 1.13, 1.59) for arrhythmias (including its subtypes: atrial fibrillation (HR = 1.34, 95%CI 1.11, 1.62), other supraventricular arrhythmias (HR = 1.83, 95%CI 1.12, 2.99), as well as 1.74 (95%CI 1.38, 2.21) for venous thromboembolism (including deep vein thrombosis: 2.27 (95%CI 1.70, 3.02); Figure 2). These findings were robust to Bonferroni correction, except for ischemic heart disease and other supraventricular arrhythmias. The corresponding IR difference was 4.21 (95%CI 2.61, 5.82) for any CVD per 1000 person-years and ranged from 1.25 (0.26, 2.24) for ischemic heart disease to 1.61 (95%CI 0.66, 2.57) for arrhythmias per 1000 person-years in sub-categories (Figure 2 and Table S5). No notable change was observed when further adjusted for family history of CVD, CCI, and metabolic status (Table S6), and self-reported IBD was removed from the analysis (Table S7). The overall results were consistent after further adjusting for the use of 5-aminosalicylates, biologic drugs, immunosuppressants, and glucocorticoids. However, for IBD and ischemic heart disease, its association was attenuated to null after further adjustment for the use of immunosuppressants or glucocorticoids (Table S8).

In the subgroup analysis, although no effect modification was observed among different age and sex groups (all *p*-interaction >0.05), the point estimates for significant CVD outcomes in the IBD-CVD analysis tend to be higher in individuals aged over 50 years at the index date than in their younger counterparts, except for heart failure, other supraventricular arrhythmias, and venous thromboembolism and its subtypes. The HRs for females were generally higher than for males. The IBD-CVD association did not differ by the time interval between the index date and recruitment date, but the HRs for arrhythmias (*p*-interaction = 0.007) and atrial fibrillation (*p*-interaction = 0.002) were attenuated to null when this interval exceeded 5 years (Table S9).

The significant association with any CVD was maintained across IBD subtypes (Figure S3). The adjusted HR was 1.53 for CD (95%CI 1.23, 1.90), 1.21 for UC (95%CI 1.05, 1.38), and 1.64 for IBD-U (95%CI 1.24, 2.17). Heart failure was significantly associated with all three IBD subtypes. Significant associations with venous thromboembolism were observed in both UC (HR = 1.74, 95%CI 1.30, 2.33) and IBD-U (HR = 2.27, 95%CI 1.20, 4.30) but not CD (HR = 1.46, 95%CI 0.85, 2.49). However, the association with cerebrovascular disease (HR = 1.70, 95%CI 1.02, 2.83) and other supraventricular arrhythmias (HR = 2.88, 95%CI 1.28, 6.49) was only present in patients with CD. Arrhythmias were significantly associated with CD (HR = 1.74, 95%CI 1.25, 2.43) and UC (HR = 1.30, 95%CI 1.06, 1.61), but not IBD-U (HR = 0.84, 95%CI 0.48, 1.49). Significant associations were detected with CD (HR = 1.79, 95%CI 1.23, 2.62) and atrial fibrillation, while a marginal association was observed for UC (HR = 1.29, 95%CI 1.02, 1.63) but not with IBD-U (HR = 0.93, 95%CI 0.50, 1.74) (Figure S3). Comparable results were observed when further adjusting for family history of CVD, CCI, metabolic status, and IBD-related medications in the sensitivity analysis (Tables S10 and S11). When a stringent definition of IBD subtypes was applied, the observed associations persisted except for UC with heart failure (HR = 1.31, 95%CI 0.95, 1.82) and atrial fibrillation (HR = 1.26, 95%CI 0.99, 1.61; Table S12). For analyses that use the step function, a decrease in magnitude was observed for CD and IBD-U with any CVD, as well as IBD with heart failure. Significant associations were maintained across follow-up periods for CD and any IBD; however, the elevated risk did not reach statistical significance when the follow-up period exceeded 10 years for IBD-U and any CVD (HR = 1.24, 95%CI 0.84, 1.83), or IBD and heart failure (HR = 1.32, 95%CI 0.97, 1.78; Table S13).

**Figure 3.**
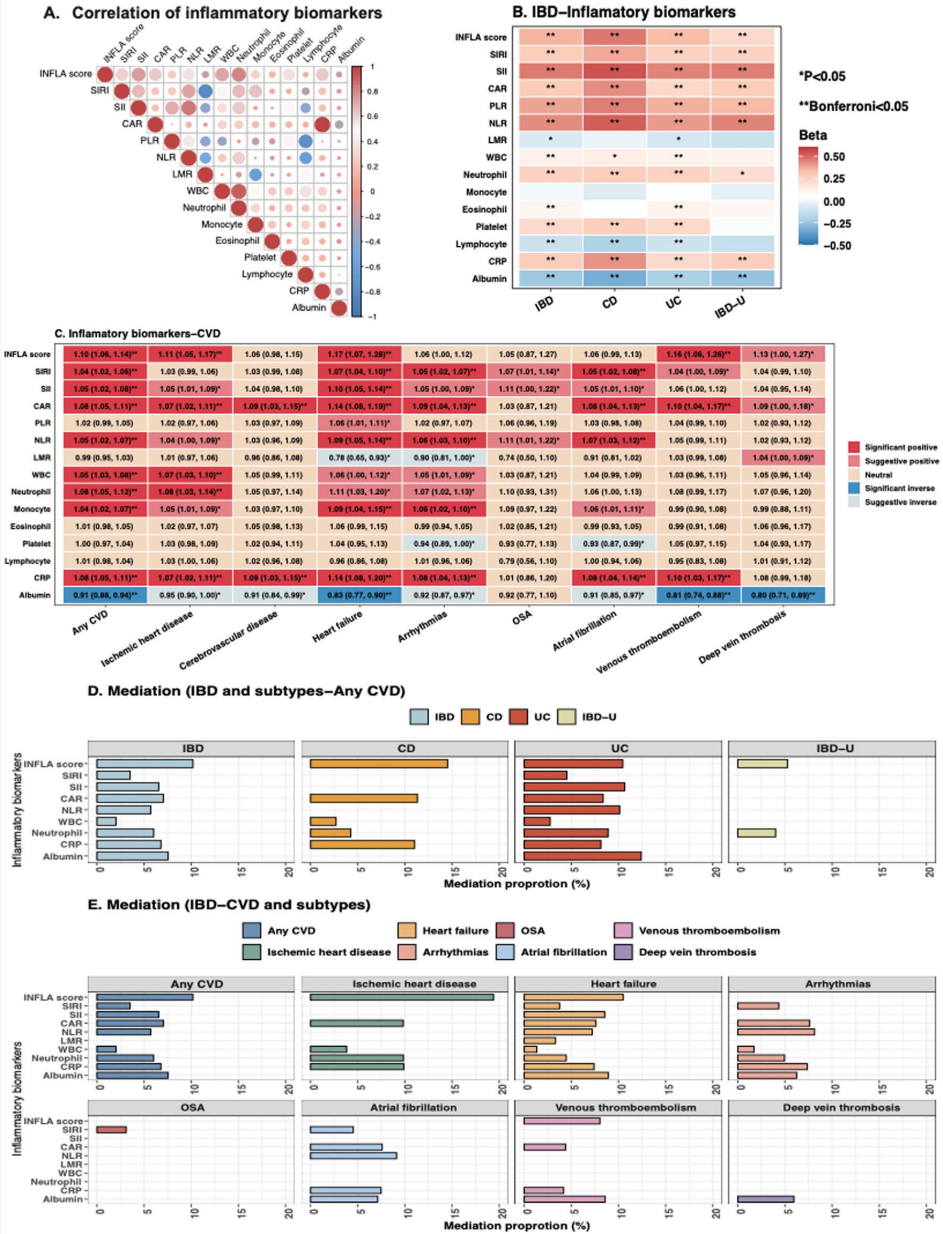
(a) Spearman correlation of 15 inflammatory biomarkers. (b) Associations of IBD and subtypes with 15 inflammatory biomarkers, using a linear-mixed effect model. The association with Bonferroni corrected *p* values<0.05 was labeled as **, while Bonferroni corrected *p* ⩾0.05 and *p* value<0.05 were labeled as * in the heatmap. (c) Associations between 15 inflammatory biomarkers and eight out of 18 CVD outcomes demonstrated at least one significant association in the IBD-CVD analysis. Labeled text is HRs with corresponding 95% CI. The association with a *p* value <0.05, but Bonferroni corrected adjusted *p* values >0.05, was considered suggestive, and adjusted *p* values <0.05 were deemed significant. (d) Bar chart of inflammatory biomarkers that significantly mediated the associations between IBD and subtypes with any CVD. (e) Bar chart of inflammatory biomarkers that significantly mediated the associations between IBD and specific CVD. In panel (c), the lower CI for the association between INFLA score and arrhythmias is 0.99975, and the *p* value is 0.051. CI, confidence intervals; CVD, cardiovascular disease; HR, hazard ratio; OSA, other supraventricular arrhythmias.

INFLA score showed the strongest overall correlation with other markers (Figure 3(a)). Using linear-mixed effect models, we identified consistent significant associations between IBD and these inflammatory biomarkers, except for eosinophils, monocytes, and LMR (Table S14). When restricted to eight CVD outcomes that reached statistical significance in the IBD-CVD analysis, all showed evidence of an association with inflammatory biomarkers (Figure 3(c)). Among these, heart failure demonstrated the most notable risk elevation (Figure 3(c) and Table S15). No nonlinear associations were found for albumin, eosinophil, monocyte, white blood cell, NLR, SII, SIRI, and INFLA score with any CVD (*p* nonlinear >0.05), although a reverse J shape was observed for CRP and CAR as well as a U-shape association was observed for lymphocyte, platelet, neutrophil, LMR, and PLR (Figure S4).

### Mediation analysis

The mediation proportion was generally higher in CD than in UC and IBD-U (Figure 2(d) and Table S16). For example, the mediation proportion of INFLA score in CD, UC, and IBD-U with any CVD was 14.5%, 10.5%, and 5.3% in primary analysis (Table S16), and 13.6%, 10.1%, and 9.9% when the stringent IBD subtype definition was applied in sensitivity mediation analysis (Table S17). The mediation proportion of inflammatory biomarkers was much higher for heart failure, ischemic heart disease, and arrhythmias than for venous thromboembolism and deep vein thrombosis (Figure 2(e) and Table S16).

## Discussion

Using data from the UK Biobank, we found that IBD patients were at a higher risk of developing any CVD compared to their matched reference individuals, regardless of IBD subtypes. Higher risks of developing specific CVD conditions (including ischemic heart disease, heart failure, arrhythmias, and venous thromboembolism) were also observed. Moreover, inflammatory biomarkers partially mediated the IBD-CVD association, with a slightly higher mediation proportion observed in CD. Heart failure, ischemic heart disease, and arrhythmias emerged as the primary CVD linked to inflammatory mediators with IBD, including well-established biomarkers related to IBD pathophysiology (e.g., CRP).

When stratified by IBD subtypes, we found that an increased risk of cerebrovascular disease was only observed in CD, which was consistent with some previous studies.^33,34^ However, it is worth noting that a meta-analysis summarized results from 12 cohort studies found a positive association between IBD and stroke, with a pooled relative risk of 1.19 (95%CI, 1.14, 1.24).^35^ In our analysis, the increased risks of heart failure were shown across IBD and three subtypes, while the relative risk estimates were lower in UC, which was in line with previous studies.^12,36^ Our results showed significant associations of CD and UC with arrhythmias, but not with IBD-U, possibly due to insufficient statistical power from a small sample size of patients with IBD-U. A nationwide cohort study from Sweden reported that patients with CD, UC, and IBD-U had 1.14- to 1.30-fold increased risk of developing new-onset arrhythmias after being diagnosed with IBD, respectively.^37^ In contrast, a US study using inpatient data reported lower rates of hospitalization-related arrhythmias in the IBD population when compared to the general population.^38^ This discrepancy might be attributed to variations in risk across different courses of IBD. As suggested in a Danish study, an increased risk of developing atrial fibrillation during IBD flares (defined by corticosteroid prescriptions, IBD hospital admissions, and biological treatment) but not in remission periods was observed.^39^ Previous studies suggested an increased risk of venous thromboembolism in both CD and UC,^28^ whereas our study found such an association only in UC. Although a distinction exists, the greater risk of venous thromboembolism in patients with UC has been noted.^28^ Also, a Mendelian randomization study suggested a causal relationship between genetically predicted UC and the risk of venous thromboembolism and deep vein thrombosis, but no such association was found in CD.^40^

We found a higher inflammation-mediated proportion of CVD in CD than in UC. For instance, the INFLA score accounted for 14.5% of the CVD risk in CD, whereas it accounted for 10.5% in UC. While the underlying mechanisms between IBD and CVD remain speculative, potential pathways may include chronic systemic inflammation and a shifted gut microbiome. The pro-inflammatory biomarkers related to IBD drive chronic inflammation, endothelial dysfunction, and smooth muscle cell proliferation, which may consequently contribute to plaque formation and impair vascular health.^41^ Although CD and UC share overlapping characteristics in clinical presentation,^42^ CD is characterized by full-thickness inflammation that affects all tissue layers of the gastrointestinal lining, while UC is typically limited to the inflammation of the mucosal layer of the colon.^42^ The gut microbiome, which might be regulated by inflammatory status, may also impact the IBD and CVD associations.^43^ A recent study from the Framingham Heart Study, the longest-running cohort on CVD, explored the metabolic mechanisms underlying the gut-heart connection and revealed a wide range of microbe-metabolite associations in patients with CVD.^5^ Dysbiosis can alter metabolites such as trimethylamine-N-oxide and consequently increase the risk of CVD.^44^ It is widely reported that dysbiosis is more pronounced in patients with CD than in UC.^45^ However, although, as suggested by our findings, some IBD and CVD associations were mediated by inflammatory biomarkers or indices, other noninflammatory pathways may also be involved and require further investigations.

The proportion mediated by inflammatory biomarkers varied between different CVD subtypes. Among them, we found that heart failure, ischemic heart disease, and arrhythmias (including atrial fibrillation) are the ones with higher proportions, which was supported by the evidence that inflammation is tightly associated with these conditions.^46–48^ In contrast, the proportion mediated by inflammatory biomarkers was much less in venous thromboembolism (including deep vein thrombosis), which might be because venous thromboembolism is particularly associated with acute severe UC, likely due to intense acute inflammation.^49^ Of note, inflammation and thrombosis are independent physiological processes, although they interact and synergize to induce inflammation-dependent coagulation, contributing to thrombotic diseases.^50^ Moreover, the risk of thrombotic events was more likely to be modulated by IBD therapies, such as surgery, corticosteroids, and methotrexate use in the absence of folate supplementation.^28^

### Strengths and limitations

This study has several strengths, including a large population-based cohort study design with a long-term follow-up period and a comprehensive investigation of a wide range of CVD outcomes. Besides, unlike previous studies in the UK Biobank that included prevalent IBD cases, we applied a matched-cohort design by only including new-onset IBD patients, which may clarify the temporal relationship between IBD and CVD and enable a longitudinal design on the mediation analysis.

Nevertheless, some limitations should be acknowledged. First, the study might be subject to unmeasured confounding due to a lack of information on disease activity, since a recent Swedish study reported a higher risk of major adverse cardiovascular events during histologic inflammation period (vs histologic remission period) and clinically active IBD period (vs clinically quiescent period).^51^ Also, IBD-related medications were not well documented in the UK Biobank. Although we observed that the association between IBD and ischemic heart disease was attenuated to null after adjustment for immunosuppressants or glucocorticoids, further study applying appropriate design and robust statistical methods (e.g., target trial emulation and new-user design) is warranted to determine the extent to which the IBD-CVD association is influenced by IBD-related medications.^12^ Second, since all biomarkers were measured at the recruitment date, rather than around IBD diagnosis, their levels may be affected by disease duration, disease activity, IBD treatments, and IBD-related complications. Although they may serve as a proxy for underlying inflammatory burden, their dynamic patterns may be inadequately reflected by a single measurement. Third, although we applied two different definitions of IBD subtypes, the possibility of misclassification led by “greedy coding” may exist, and the results related to IBD-U (which had limited cases) should be interpreted with caution. However, the overall consistent findings increase the reliability of our results. Fourth, changes in lifestyle behavior after IBD diagnosis could not be considered due to a lack of data; future studies with such information are warranted. Fifth, while our study extended that the inflammation markers play an integral mediating role in the development of incident CVD among patients with IBD, further research is needed to determine the most risk-relevant markers and optimal timing and thresholds for measurement. To investigate the mediation role of inflammation biomarkers, we had to enroll IBD patients who were healthy enough to survive until the recruitment date. We cannot rule out that these patients were slightly healthier than the average IBD patient, which may underestimate the IBD-CVD association. Finally, the UK Biobank is dominated by European ancestry and enrolled participants aged between 40 and 69 years. Considering the relatively younger age of IBD onset (usually 20–40 years old), cases diagnosed in childhood or early adulthood could not be covered. Therefore, generalizability of our findings should be cautious, and further studies with more diverse populations are warranted.

## Conclusion

In this matched-cohort study, we found that patients with IBD were at an increased risk of developing any and specific CVD, including heart failure, ischemic heart disease, arrhythmias (including atrial fibrillation and other supraventricular arrhythmias), and venous thromboembolism (including deep vein thrombosis) Moreover, we observed that the increased risks were partially mediated by inflammatory biomarkers, with the mediation proportion generally appearing higher in CD and specific CVD subtypes (including ischemic heart disease, heart failure, and arrhythmias). Our findings suggested that reducing inflammatory status may help to alleviate cardiac burden in patients with IBD in clinical practice.

## Supplemental Material

sj-docx-1-tag-10.1177_17562848261424145 – Supplemental material for Association of inflammatory bowel disease with cardiovascular disease, and the mediating role of inflammation: a matched-cohort study Supplemental material, sj-docx-1-tag-10.1177_17562848261424145 for Association of inflammatory bowel disease with cardiovascular disease, and the mediating role of inflammation: a matched-cohort study by Jiawei Geng, Jie Chen, Ola Olén, Jonas Halfvarson, Johan Sundström, Shuai Yuan, Jonas F. Ludvigsson and Jiangwei Sun in Therapeutic Advances in Gastroenterology

sj-docx-2-tag-10.1177_17562848261424145 – Supplemental material for Association of inflammatory bowel disease with cardiovascular disease, and the mediating role of inflammation: a matched-cohort study Supplemental material, sj-docx-2-tag-10.1177_17562848261424145 for Association of inflammatory bowel disease with cardiovascular disease, and the mediating role of inflammation: a matched-cohort study by Jiawei Geng, Jie Chen, Ola Olén, Jonas Halfvarson, Johan Sundström, Shuai Yuan, Jonas F. Ludvigsson and Jiangwei Sun in Therapeutic Advances in Gastroenterology

## Declarations

**Ethics approval and consent to participate:** Ethical approval of the North West Multicenter Research Ethics Committee (21/NW/0157), and written informed consent were obtained from all participants. This study was conducted with the UK Biobank Resource under application number 79612 and 73595.

**Consent for publication:** Not applicable.

**Availability of data and materials:** The datasets analyzed during the current study are available in a public open-access repository (https://www.ukbiobank.ac.uk/).
