# Supplementary material for: Association of inflammatory bowel disease with cardiovascular disease, and the mediating role of inflammation: a matched-cohort study
Source: Ther Adv Gastroenterol. 2026 Feb 24;19:17562848261424145. doi: 10.1177/17562848261424145 (PMC12932890; doi:10.1177/17562848261424145)
Supplement: sj-docx-2-tag-10.1177_17562848261424145 – Supplemental material for Association of inflammatory bowel disease with cardiovascular disease, and the mediating role of inflammation: a matched-cohort study [file sj-docx-2-tag-10.1177_17562848261424145.docx]

**Supplementary tables and figures**

[Table S1. Ascertainment of inflammatory bowel disease (IBD). 3](#_Toc219139900)

[Table S2. Ascertainment of cardiovascular disease (CVD). 4](#_Toc219139901)

[Table S3. Definition of inflammatory biomarkers. 6](#_Toc219139902)

[Table S4. Definition of covariates. 7](#_Toc219139903)

[Table S5. Incident cardiovascular disease (CVD) in patients with inflammatory bowel disease (IBD) and their matched reference individuals. 10](#_Toc219139904)

[Table S6. Association between inflammatory bowel disease (IBD) and the risk of cardiovascular disease (CVD) outcomes with further adjustment for history of CVD, metabolic status, and Charlson comorbidity index (CCI). 11](#_Toc219139905)

[Table S7. Association between inflammatory bowel disease (IBD) among participants ascertained IBD by primary healthcare or inpatient data (N=1,416) and the risk of cardiovascular disease (CVD). 12](#_Toc219139906)

[Table S8. Association between inflammatory bowel disease (IBD) and the risk of cardiovascular disease (CVD) outcomes with further adjustment for 5-aminosalicylates, biologic drugs, immunosuppressants, and glucocorticoids 13](#_Toc219139907)

[Table S9. Subgroup analysis of the associations between inflammatory bowel disease (IBD) and cardiovascular disease (CVD) outcomes by age, sex, and time interval between index date and recruitment. 15](#_Toc219139908)

[Table S10. Association between Crohn’s disease (CD), Ulcerative colitis (UC), and IBD-unclassified (IBD-U) and the risk of cardiovascular disease (CVD) outcomes with further adjustment for history of CVD, metabolic status, and Charlson comorbidity index (CCI). 18](#_Toc219139909)

[Table S11. Association between Crohn’s disease (CD), Ulcerative colitis (UC), and IBD-unclassified (IBD-U) and the risk of cardiovascular disease (CVD) outcomes with further adjustment for 5-aminosalicylates, biologic drugs, immunosuppressants, and glucocorticoids 21](#_Toc219139910)

[Table S12. Association between stringent defined IBD subtypes and the risk of cardiovascular disease (CVD) outcomes. 24](#_Toc219139911)

[Table S13. Results of step function for the analysis with unmet proportional hazard assumptions over different time intervals (0-5, 5-10, >10 years follow-up) 26](#_Toc219139912)

[Table S14. Associations of inflammatory bowel disease (IBD), Crohn’s disease (CD), ulcerative colitis (UC), and IBD-unclassified (IBD-U) with inflammatory biomarkers. 27](#_Toc219139913)

[Table S15. Associations of inflammatory biomarkers with nine cardiovascular disease (CVD) outcomes. 29](#_Toc219139914)

[Table S16. Results of mediation analysis. 32](#_Toc219139915)

[Table S17. Results of mediation analysis using stringent IBD subtype definition. 43](#_Toc219139916)

[Figure S1. Source of inflammatory bowel disease (IBD). 52](#_Toc219139917)

[Figure S2. Directed acyclic graph (DAG) of the potential causal relationship between exposure (IBD) and outcome (CVD) and other factors influencing the relationship. 53](#_Toc219139918)

[Figure S3. Forest plot of associations between Crohn’s disease (CD), ulcerative colitis (UC), and IBD-unclassified (IBD-U) and cardiovascular disease (CVD). Incidence rate (IR) difference expressed as per 1,000 person-years. CI, confidence interval. The association with Bonferroni corrected *P* values<0.05 was labelled as **, while Bonferroni corrected *P*≥0.05 and *P* value<0.05 were labelled as *. 54](#_Toc219139919)

[Figure S4. Restricted cubic spline function (RCS) assessing the nonlinear association between inflammatory biomarkers and incident any cardiovascular disease (CVD). Median value was used as the reference value for the RCS curve. 55](#_Toc219139920)

# Table S1. Ascertainment of inflammatory bowel disease (IBD).

| **Diseases** | **UK Biobank code** | **ICD code** | **Primary care code** |
| --- | --- | --- | --- |
| Crohn’s disease | 20002 (1462) | ICD-9: 555  ICD-10: K50 | **Read V2:** "J40.." "J400." "J4000" "J4001" "J4002" "J4003" "J4004" "J400z" "J401." "J4010" "J4011" "J401z" "J402." "J40z." "J08z9" "J4005" "J4012" "Jyu40";  **Read V3:** "J08z9" "J400." "J4000" "J4001" "J4002" "J4003" "J4004" "J400z" "J401." "J4010" "J4011" "J402." "J40z." "X20Pq" "X300J" "X301b" "X302j" "X302r" "X302s" "X302t" "X302u" "X303f" "X303j" "X304G" "X3050" "XE0af" "XE2QL" "Xa0lh" "J401z" "J5109" "Jyu40" "XE0ae" "Xa8Eh" "XaK6C" "XaK6D" |
| Ulcerative colitis | 20002 (1463) | ICD-9: 556  ICD-10: K51 | **Read V2:** "J41.." "J410." "J4100" "J4101" "J4102" "J4103" "J410z" "J411." "J412." "J41y." "J41y0" "J41y1" "J41yz" "J41z." "J4104" "J413." "J438." "Jyu41";  **Read V3:** "J410." "J4100" "J4103" "J410z" "J41y." "J41y0" "J41y1" "J41yz" "J41z." "X302j" "X302m" "X302y" "X3030" "X303e" "X303f" "X3047" "X304G" "X305p" "X30Bn" "XE0ag" "J41.." "Jyu41" "X302s" "X302z" "X304H" "XE0ae" "Xa8Eh" "XaB5z" "XaK6E" "XaYzX" "XaZ2j" |

ICD, International Classification of Diseases; Read v2, Read Version 2; Read v3, Read Version 3.

# Table S2. Ascertainment of cardiovascular disease (CVD).

| **Diseases** | **UK Biobank self-reported code** | **ICD code** |
| --- | --- | --- |
| **Ischemic heart disease (IHD)** | 20002 (1074-angina; 1075-heart attack/myocardial infarction);  6150 (1-heart attack, 2-angina diagnosed by doctor); 3627 (Age angina diagnosed); 3894 (Age heart attack diagnosed) | ICD-9: 410-414  ICD-10: I20-I25 |
| **-**Myocardial infarction/heart attack | 20002 (1075-heart attack/myocardial infarction);  6150 (1-heart attack diagnosed by doctor); 3894 (Age heart attack diagnosed) | ICD-9: 410  ICD-10: I21-I22 |
| **Cerebrovascular disease** | 2002 (1081-stoke, 1583-ischaemic stroke, 1086-subarachnoid haemorrhage, 1491-brain haemorrhage, 1082-transient ischaemic attack (tia), 1083-subdural haemorrhage/haematoma, 1425-cerebral aneurysm); 4056 (Age stroke diagnosed); 6150 (3-stroke) | ICD-9: 430-434, 436-438  ICD-10: I60-I69 |
| **-**Stroke | 20002 (1081-stroke, 1491-brain haemorrhage, 1086-subarachnoid haemorrhage, 1583-ischaemic stroke); 4056 (Age stroke diagnosed); 6150 (3-stroke) | ICD-9: 430-431, 434, 436  ICD-10: I60-I61, I63-I64 |
| **--**Hemorrhagic stroke | 20002 (1491-brain haemorrhage, 1086-subarachnoid haemorrhage) | ICD-9: 430, 431  ICD-10: I60-I61 |
| **--**Ischemic stroke | 20002 (1583-ischaemic stroke) | ICD-9: 434  ICD-10: I63 |
| **Myocarditis** | 20002 (1426-myocarditis) | ICD-9: 422, 429  ICD-10: I40, I41, I514 |
| **Heart failure** | 20002 (1076-heart failure/pulmonary odema) | ICD-9: 428  ICD-10: I50, I110 |
| **Arrhythmias** | 20002 (1471-atrial fibrillation, 1483-atrial flutte, 1484-wolff parkinson white/wpw syndrome, 1485-irregular heart beat, 1486-sick sinus syndrome, 1487-svt /supraventricular tachycardia, 1077-heart arrhythmia) | ICD-9: 4260, 4261, 4265,4266, 4267, 4268, 4269, 4270, 4271, 4273, 4274, 4275, 7981  ICD-10: I441, I442, I452, I453, I456, I459, I460, I461, I469, I470, I472, I471, I48, I490, I495, R960 |
| **-**Atrial fibrillation | 20002 (1471-atrial fibrillation, 1483-atrial flutte, 1484-wolff parkinson white/wpw syndrome, 1485-irregular heart beat, 1486-sick sinus syndrome, 1487-svt /supraventricular tachycardia) | ICD-9: 4273  ICD-10: I48 |
| -Bradyarrhythmia | / | ICD-9: 4260, 4261, 4265, 4266, 4269  ICD-10: I441, I442, I452, I453, I459, I495 |
| -Other supraventricular arrhythmias | / | ICD-9: 4267, 4268, 4270  ICD-10: I456, I471 |
| -Cardiac arrest and ventricular arrhythmias | 20002 (1077-heart arrhythmia) | ICD-9: 4271, 4274, 4275, 7981  ICD-10: I460, I461, I469, I470, I472, I490, R960 |
| **Peripheral artery disease (PAD)** | 20002 (1067-peripheral vascular disease) | ICD-9: 440, 443.9  ICD-10: I70, I73.9 |
| **Venous thromboembolism (VTE)** | 20002, 4012, 6152, 4022 | ICD-9: 415, 451-453  ICD-10: I26, I80-I82 |
| -pulmonary embolism (PE) | 20002 (1093- pulmonary embolism +/- dvt); 6152 (7-Blood clot in the lung); 4022 (Age pulmonary embolism (blood clot in lung) diagnosed) | ICD-9: 415  ICD-10: I26 |
| -deep vein thrombosis (DVT) | 20002 (1094- deep venous thrombosis (dvt));  6152 (5-Blood clot in the leg (DVT)); 4012 (Age deep-vein thrombosis (DVT, blood clot in leg) diagnosed) | ICD-9: 451-453  ICD-10: I80-I82 |

Disease definitions constructed using ICD-9, ICD-10, and self-reported data with choice-, or disease-specific codes between brackets are shown. ICD, international Classification of Diseases.

# Table S3. Definition of inflammatory biomarkers.

| **Inflammatory markers** | **UK Biobank code** | **Definitions** |
| --- | --- | --- |
| Albumin | 30600 | Continuous, g/L; Measured by BCG analysis on a Beckman Coulter AU5800 |
| C-reactive protein (CRP) | 30710 | Continuous, mg/L; Measured by immunoturbidimetric - high sensitivity analysis on a Beckman Coulter AU5800 |
| Lymphocyte count | 30120 | Continuous, 10^9 cells/Litre; Lymphocyte count is the proportion of (lymphoctyes/100) x white blood cell count |
| Platelet count | 30080 | Continuous, 10^9 cells/Litre |
| *Subtypes of White blood cell (leukocyte)* | | |
| Eosinophil count | 30150 | Continuous, 10^9 cells/Litre; Eosinophils count is the proportion of (eosinophils /100) x white blood cell count. |
| Monocyte count | 30130 | Continuous, 10^9 cells/Litre; Monocyte count is the proportion of (monoctyes/100) x white blood cell count. |
| Neutrophil count | 30140 | Continuous, 10^9 cells/Litre; Neutrophils count is the proportion of (neutrophils/100) x white blood cell count. |
| White blood cell (leukocyte) count | 30000 | Continuous, 10^9 cells/Litre; Analyser operating range was 0 to 9x10^11 cells/Litre. Typical reference range is 3.5x10^9 to 9.6x10^9 cells/Litre. |
| ***Composite measures*** |  |  |
| Lymphocyte to monocyte ratio (LMR) | 30120, 30130 | Continuous variable. |
| Neutrophil to lymphocyte ratio (NLR) | 30140, 30120 | Continuous variable. |
| Platelet to lymphocyte ratio (PLR) | 30080, 30120 | Continuous variable. |
| C-reactive protein to albumin ratio (CAR) | 30710, 30600 | Continuous variable. |
| Systemic immune-inflammatory index (SII) | 30080, 30140, 30000 | Continuous variable. Systemic immune-inflammation index (SII) = peripheral blood platelet × neutrophil counts/lymphocyte counts |
| Systemic inflammation response index (SIRI) | 30080, 30130, 30000 | Continuous variable.Systemic inflammation response index (SIRI) = peripheral neutrophil counts × monocyte counts/lymphocyte counts |
| INFLA-score | 30710, 30000, 30080, 30140 | Continuous variable. Aliquoted the four indicators (C-reactive protein, white blood cell count, platelet count, and the neutrophil-to-lymphocyte ratio), with the highest four deciles assigned values +1 to +4 (7^th^ to 10^th^) and the lowest four deciles assigned values -4 to -1 (1^st^ to 4^th^) and summed to obtain the score. |

# Table S4. Definition of covariates.

| **Covariates** | **Definitions** |
| --- | --- |
| **Age at index date** | Continuous variable.  Age at index date is based on the participant's date of birth and index date, truncated to a full year. |
| **Sex** | Categorical variable, male and female.  Acquired from central registry at recruitment. |
| **Ethnicity** | Categorical variable, white and non-white.  UK Biobank Touchscreen questionnaire asked the ethnic background. White included British, Irish, and any other white background. |
| **Education attainment** | Categorical variable, college or university degree and above, and high school and below. |
| **Townsend deprivation index (TDI)** | Categorical variable.  Townsend deprivation index was conducted as a complex indicator of socioeconomic status based on the preceding national census output areas at recruitment using the method mentioned online (<https://biobank.ndph.ox.ac.uk/showcase/label.cgi?id=76>).  We tertiled TDI as low, moderate, and high deprivation group among the whole UK Biobank participants. |
| **Body mass index** | Categorical variable, <25 weight/height^2^, kg/m^2^, 25-30 kg/m^2^, and ≥30 kg/m^2^. |
| **Smoking status** | Categorical variable, non-smoker, previous and current smoker. |
| **Alcohol drinking status** | Categorical variable, current and noncurrent drinkers |
| **Physical activity** | Categorical variable, regular and irregular.  UK Biobank Touchscreen questionnaire on the reported type and duration of physical activity (including walking, DIY, moderate and vigorous physical activity, strenuous sports, etc) recommended by the American Heart Association.  One of the following is equal to 1 score:  1.≥150 minutes of moderate activity per week.  2.≥ 75 minutes of vigorous activity per week.  3. Equivalent combination.  4. Moderate physical activity at least 5 days a week or vigorous activity once a week.  1 score and more indicated regular physical activity. |
| **A healthy diet** **(cardioprotective diet)** | Categorical variable, adherence to a healthy diet and not.  UK Biobank Food Frequency Questionnaire asked the frequency of intake of a range of common food and drink items. One diet point was given if the intakes were met for: 1. Fruits: ≥ 3 servings/day 2. Vegetables: ≥ 3 servings/day 3. Fish: ≥2 servings/week 4. Whole grains: ≥ 3 servings/day 5. Refined grains: ≤2 servings/day 6. Processed meats: ≤ 1 servings/week 7. Unprocessed red meats: ≤ 2 servings/week  A diet score ≥4 indicated a high adherence to a healthy dietary pattern. |
| **Hypertension** | Categorical variable, with and without. Ascertained by self-reported, primary health care, and inpatient healthcare data.  20002 (1065-hypertension, 1072- essential hypertension), 6150 (4-High blood pressure), 2966 (Age high blood pressure diagnosed); ICD-9: 401-405; ICD-10: I10-I13, I15 |
| **Hyperlipidaemia** | Categorical variable, with and without. Ascertained by self-reported, primary health care, and inpatient healthcare data.  20002 (1473- high cholesterol); ICD-9: 272; ICD-10: E78 |
| **Diabetes** | Categorical variable, with and without. Ascertained by self-reported, primary health care, and inpatient healthcare data.  20002 (1220-diabetes, 1222-type 1 diabetes, 1223-type 2 diabetes), 2976 (Age diabetes diagnosed), 2443 (1-Diabetes diagnosed by doctor); ICD-9: 250; ICD-10: E10-E14 |
| **Family history of cardiovascular disease (CVD)** | Categorical variable, with and without.  Defined as whether father or mother ever suffered from stroke or heart disease at recruitment. |
| **Metabolic status** | Categorical variable, healthy and unhealthy.  Unhealthy metabolic status was considered when participants satisfied more than one of the following criteria: hyperlipidemia (≥1.7 mmol/L) or taking lipid-lowering drugs, elevated systolic blood pressure (≥130 mmHg) or diastolic blood pressure (≥85 mmHg) or taking anti-hypertensive drugs, elevated random glucose (≥11.1 mmol/L) and glycated hemoglobin (≥42 mmol/mol) or medications for diabetes (insulin and oral anti-diabetic drugs). |
| **Charlson Comorbidity Index (CCI)** | Continuous variable.  CCI scores were computed based on 17 comorbidities at the index date for each participant, each assigned a weighted value according to their severity and mortality risk. The higher the score, the more likely the predicted negative outcome (like death) will occur. This index is widely used to adjust for the severity of comorbid conditions in risk adjustment models. |
| 5-aminosalicylates (5-ASA) | Categorical variable, yes or no.  Participants were classified as 5-ASA users if reported use of any of the following: Mesalazine, Sulfasalazine, Olsalazine, Balsalazide. |
| Biologic drugs | Categorical variable, yes or no.  Participants were classified as biologic drugs users if reported use of any of the following: Adalimumab. |
| Immunosuppressants | Categorical variable, yes or no.  Participants were classified as immunosuppressants users if reported use of any of the following: Azathioprine, Mercaptopurine, Methotrexate, Cyclosporine A, Tacrolimus. |
| Glucocorticoids | Defined by participants who reported following: Participants were classified as glucocorticoids users if reported use of any of the following: Budesonide, Dexamethasone, Hydrocortisone, Methylprednisolone, Prednisolone, Prednisone. |

# Table S5. Incident cardiovascular disease (CVD) in patients with inflammatory bowel disease (IBD) and their matched reference individuals.

|  | **No. of events  (IR, per 1,000 person-years)** | | **IR difference (95%CI), per 1,000 person-years** | **Model 1 ^a^** | | **Model 2 ^b^** | |
| --- | --- | --- | --- | --- | --- | --- | --- |
| **CVD outcomes** | **Patients** | **Reference** |  | **HR (95%CI)** | ***P*** | **HR (95%CI)** | ***P*** |
| Any CVD | 391 (15.76) | 2960 (11.55) | 4.21 (2.61,5.82) | 1.35 [1.22, 1.50] | 2.38E-08* | 1.34 [1.20, 1.49] | 8.85E-08* |
| Ischemic heart disease | 163 (6.24) | 1325 (4.99) | 1.25 (0.26,2.24) | 1.22 [1.04, 1.44] | 0.015 | 1.20 [1.02, 1.41] | 0.032 |
| Myocardial infarction | 43 (1.6) | 379 (1.4) | 0.2 (-0.3,0.7) | 1.11 [0.81, 1.52] | 0.513 | 1.08 [0.78, 1.48] | 0.648 |
| Cerebrovascular disease | 68 (2.54) | 599 (2.21) | 0.33 (-0.3,0.95) | 1.11 [0.87, 1.43] | 0.406 | 1.09 [0.85, 1.41] | 0.489 |
| Stroke | 40 (1.49) | 355 (1.31) | 0.18 (-0.3,0.66) | 1.11 [0.80, 1.54] | 0.532 | 1.09 [0.78, 1.51] | 0.617 |
| Hemorrhagic stroke | 11 (0.41) | 96 (0.35) | 0.05 (-0.2,0.3) | 1.12 [0.60, 2.09] | 0.727 | 1.08 [0.58, 2.02] | 0.816 |
| Ischemic stroke | 26 (0.96) | 245 (0.9) | 0.06 (-0.32,0.45) | 1.06 [0.70, 1.58] | 0.791 | 1.06 [0.71, 1.59] | 0.777 |
| Myocarditis | 12 (0.44) | 101 (0.37) | 0.07 (-0.19,0.33) | 1.18 [0.65, 2.15] | 0.587 | 1.19 [0.65, 2.17] | 0.577 |
| Heart failure | 86 (3.23) | 511 (1.89) | 1.34 (0.64,2.04) | 1.65 [1.31, 2.07] | 1.87E-05* | 1.61 [1.28, 2.02] | 5.37E-05* |
| Arrhythmias | 156 (5.9) | 1146 (4.29) | 1.61 (0.66,2.57) | 1.35 [1.14, 1.60] | 3.98E-04* | 1.34 [1.13, 1.59] | 0.001* |
| Atrial fibrillation | 124 (4.66) | 908 (3.38) | 1.28 (0.44,2.13) | 1.36 [1.13, 1.64] | 0.001* | 1.34 [1.11, 1.62] | 0.002* |
| Bradyarrhythmia | 19 (0.7) | 136 (0.5) | 0.2 (-0.12,0.53) | 1.39 [0.86, 2.25] | 0.176 | 1.49 [0.92, 2.41] | 0.108 |
| Other supraventricular arrhythmias | 19 (0.7) | 105 (0.39) | 0.32 (-0.01,0.64) | 1.81 [1.11, 2.95] | 0.018 | 1.83 [1.12, 2.99] | 0.016 |
| Cardiac arrest and ventricular arrhythmias | 19 (0.7) | 168 (0.62) | 0.09 (-0.24,0.41) | 1.09 [0.68, 1.76] | 0.710 | 1.09 [0.67, 1.75] | 0.734 |
| Peripheral artery disease | 20 (0.74) | 157 (0.58) | 0.16 (-0.17,0.5) | 1.18 [0.74, 1.88] | 0.485 | 1.14 [0.71, 1.82] | 0.579 |
| Venous thromboembolism | 82 (3.07) | 467 (1.72) | 1.34 (0.66,2.02) | 1.75 [1.38, 2.21] | 3.28E-06* | 1.74 [1.38, 2.21] | 4.06E-06* |
| Pulmonary embolism | 29 (1.07) | 253 (0.93) | 0.14 (-0.26,0.55) | 1.13 [0.77, 1.67] | 0.524 | 1.13 [0.76, 1.66] | 0.549 |
| Deep vein thrombosis | 59 (2.2) | 259 (0.95) | 1.25 (0.67,1.82) | 2.27 [1.71, 3.01] | 1.51E-08* | 2.27 [1.70, 3.02] | 1.82E-08* |

^a^ Model 1 adjusted for age, sex, Townsend deprivation index, ethnicity, and education.

^b^ Model 2 further adjusted for body mass index, alcohol drinking, smoking status, physical activity, history of hypertension, diabetes, hyperlipidemia at index date, and adherence to cardioprotective diet.

Asterisks (*) next to *P* indicate significance after Bonferroni correction.

CI, confidence interval; CVD, cardiovascular disease; HR, hazard ratio; IR, incidence rate

# Table S6. Association between inflammatory bowel disease (IBD) and the risk of cardiovascular disease (CVD) outcomes with further adjustment for history of CVD, metabolic status, and Charlson comorbidity index (CCI).

|  | **Further adjusted for family history of CVD ^a^** | | **Further adjusted for metabolic status ^b^** | | **Further adjusted for CCI ^c^** | |
| --- | --- | --- | --- | --- | --- | --- |
| **CVD outcomes** | **HR (95%CI)** | ***P*** | **HR (95%CI)** | ***P*** | **HR (95%CI)** | ***P*** |
| Any CVD | 1.34 [1.21, 1.49] | 5.38E-08 | 1.34 [1.20, 1.49] | 8.40E-08 | 1.32 [1.19, 1.47] | 2.33E-07 |
| Ischemic heart disease | 1.20 [1.02, 1.42] | 0.027 | 1.20 [1.02, 1.41] | 0.029 | 1.19 [1.01, 1.40] | 0.037 |
| Myocardial infarction | 1.08 [0.79, 1.49] | 0.619 | 1.08 [0.79, 1.49] | 0.622 | 1.07 [0.78, 1.47] | 0.673 |
| Cerebrovascular disease | 1.10 [0.85, 1.41] | 0.464 | 1.09 [0.85, 1.41] | 0.489 | 1.08 [0.84, 1.40] | 0.531 |
| Stroke | 1.09 [0.79, 1.52] | 0.595 | 1.09 [0.78, 1.51] | 0.616 | 1.08 [0.78, 1.50] | 0.651 |
| Hemorrhagic stroke | 1.08 [0.58, 2.02] | 0.809 | 1.08 [0.57, 2.02] | 0.817 | 1.12 [0.60, 2.11] | 0.715 |
| Ischemic stroke | 1.07 [0.71, 1.60] | 0.758 | 1.06 [0.71, 1.59] | 0.775 | 1.04 [0.69, 1.57] | 0.844 |
| Myocarditis | 1.20 [0.66, 2.19] | 0.553 | 1.18 [0.65, 2.16] | 0.584 | 1.21 [0.66, 2.21] | 0.533 |
| Heart failure | 1.61 [1.28, 2.03] | 4.56E-05 | 1.61 [1.28, 2.03] | 5.15E-05 | 1.58 [1.25, 1.99] | 1.10E-04 |
| Arrhythmias | 1.34 [1.14, 1.59] | 0.001 | 1.34 [1.13, 1.59] | 0.001 | 1.33 [1.12, 1.57] | 0.001 |
| Atrial fibrillation | 1.34 [1.11, 1.62] | 0.002 | 1.34 [1.11, 1.62] | 0.002 | 1.32 [1.09, 1.60] | 0.004 |
| Bradyarrhythmia | 1.48 [0.92, 2.41] | 0.110 | 1.50 [0.92, 2.43] | 0.101 | 1.45 [0.89, 2.35] | 0.135 |
| Other supraventricular arrhythmias | 1.83 [1.12, 2.99] | 0.017 | 1.82 [1.11, 2.98] | 0.017 | 1.82 [1.11, 2.99] | 0.017 |
| Cardiac arrest and ventricular arrhythmias | 1.09 [0.68, 1.75] | 0.726 | 1.09 [0.68, 1.76] | 0.710 | 1.08 [0.67, 1.75] | 0.746 |
| Peripheral artery disease | 1.15 [0.72, 1.84] | 0.561 | 1.15 [0.72, 1.84] | 0.561 | 1.13 [0.71, 1.80] | 0.613 |
| Venous thromboembolism | 1.75 [1.38, 2.22] | 3.56E-06 | 1.74 [1.38, 2.21] | 4.21E-06 | 1.71 [1.35, 2.17] | 8.66E-06 |
| Pulmonary embolism | 1.13 [0.77, 1.66] | 0.540 | 1.13 [0.77, 1.66] | 0.538 | 1.09 [0.74, 1.60] | 0.666 |
| Deep vein thrombosis | 2.28 [1.71, 3.03] | 1.51E-08 | 2.25 [1.69, 2.99] | 2.50E-08 | 2.27 [1.70, 3.02] | 1.96E-08 |

^a^ adjusted for age, sex, Townsend deprivation index, ethnicity, education, body mass index, alcohol drinking, smoking status, physical activity, history of hypertension, diabetes, hyperlipidemia at index date, adherence to cardioprotective diet, and family history of CVD.

^b^ adjusted for age, sex, Townsend deprivation index, ethnicity, education, body mass index, alcohol drinking, smoking status, physical activity, history of hypertension, diabetes, hyperlipidemia at index date, adherence to cardioprotective diet, and metabolic status.

^c^ adjusted for age, sex, Townsend deprivation index, ethnicity, education, body mass index, alcohol drinking, smoking status, physical activity, history of hypertension, diabetes, hyperlipidemia at index date, adherence to cardioprotective diet, and Charlson comorbidity index.

*CI, confidence interval; CVD, cardiovascular disease; CCI, Charlson comorbidity index; HR, hazard ratio.*

# Table S7. Association between inflammatory bowel disease (IBD) among participants ascertained IBD by primary healthcare or inpatient data (N=1,416) and the risk of cardiovascular disease (CVD).

| **CVD outcomes** | **Patient IR** | **Reference IR** | **IR difference (95%CI)** | **HR (95%CI)** | ***P*** ^a^ |
| --- | --- | --- | --- | --- | --- |
| Any CVD | 369 (15.64) | 2812 (11.56) | 4.09 (2.45,5.73) | 1.32 [1.18, 1.47] | 8.67E-07** |
| Ischemic heart disease | 156 (6.28) | 1253 (4.97) | 1.31 (0.29,2.33) | 1.20 [1.02, 1.42] | 0.032* |
| Myocardial infarction | 42 (1.64) | 357 (1.39) | 0.26 (-0.26,0.78) | 1.10 [0.80, 1.51] | 0.573 |
| Cerebrovascular disease | 64 (2.51) | 558 (2.17) | 0.34 (-0.3,0.98) | 1.11 [0.86, 1.44] | 0.431 |
| Stroke | 37 (1.45) | 337 (1.31) | 0.14 (-0.35,0.63) | 1.06 [0.75, 1.49] | 0.739 |
| Hemorrhagic stroke | 11 (0.43) | 91 (0.35) | 0.08 (-0.19,0.34) | 1.11 [0.59, 2.09] | 0.744 |
| Ischemic stroke | 23 (0.9) | 229 (0.89) | 0.01 (-0.37,0.39) | 1.01 [0.65, 1.55] | 0.978 |
| Myocarditis | 10 (0.39) | 99 (0.38) | 0.01 (-0.25,0.26) | 1.00 [0.52, 1.92] | 0.999 |
| Heart failure | 80 (3.16) | 488 (1.9) | 1.26 (0.55,1.97) | 1.54 [1.22, 1.96] | 3.54E-04** |
| Arrhythmias | 147 (5.85) | 1105 (4.35) | 1.49 (0.52,2.47) | 1.31 [1.10, 1.55] | 0.003** |
| Atrial fibrillation | 116 (4.59) | 878 (3.44) | 1.14 (0.28,2.01) | 1.30 [1.07, 1.57] | 0.009* |
| Bradyarrhythmia | 18 (0.7) | 133 (0.51) | 0.19 (-0.15,0.52) | 1.44 [0.87, 2.36] | 0.152 |
| Other supraventricular arrhythmias | 19 (0.74) | 102 (0.39) | 0.35 (0,0.69) | 1.88 [1.15, 3.08] | 0.012* |
| Cardiac arrest and ventricular arrhythmias | 19 (0.74) | 161 (0.62) | 0.12 (-0.23,0.46) | 1.13 [0.70, 1.82] | 0.625 |
| Peripheral artery disease | 20 (0.78) | 147 (0.57) | 0.21 (-0.14,0.56) | 1.21 [0.76, 1.94] | 0.417 |
| Venous thromboembolism | 77 (3.03) | 447 (1.74) | 1.29 (0.6,1.98) | 1.70 [1.33, 2.17] | 2.02E-05** |
| Pulmonary embolism | 26 (1.01) | 243 (0.94) | 0.07 (-0.33,0.48) | 1.04 [0.70, 1.57] | 0.836 |
| Deep vein thrombosis | 57 (2.23) | 247 (0.96) | 1.28 (0.68,1.87) | 2.28 [1.71, 3.05] | 2.51E-08** |

^a^ adjusted for age, sex, Townsend deprivation index, ethnicity, education, body mass index, alcohol drinking, smoking status, physical activity, history of hypertension, diabetes, hyperlipidemia at index date, and adherence to cardioprotective diet.

*CI, confidence interval; CVD, cardiovascular disease; HR, hazard ratio; IR, incident rate.*

# Table S8. Association between inflammatory bowel disease (IBD) and the risk of cardiovascular disease (CVD) outcomes with further adjustment for 5-aminosalicylates, biologic drugs, immunosuppressants, and glucocorticoids

|  | **Further adjusted for use of 5-aminosalicylates ^a^** | | **Further adjusted for use of biologic drugs ^b^** | | **Further adjusted for use of immunosuppressants ^c^** | | **Further adjusted for use of glucocorticoids ^d^** | |
| --- | --- | --- | --- | --- | --- | --- | --- | --- |
| **CVD outcomes** | **HR (95%CI)** | ***P*** | **HR (95%CI)** | ***P*** | **HR (95%CI)** | ***P*** | **HR (95%CI)** | ***P*** |
| Any CVD | 1.49 [1.32, 1.69] | 3.79E-10 | 1.34 [1.20, 1.49] | 8.04E-08 | 1.29 [1.16, 1.44] | 6.24E-06 | 1.29 [1.16, 1.44] | 3.05E-06 |
| Ischemic heart disease | 1.38 [1.14, 1.67] | 0.001 | 1.20 [1.02, 1.41] | 0.031 | 1.17 [0.99, 1.39] | 0.067 | 1.15 [0.97, 1.36] | 0.107 |
| Myocardial infarction | 1.13 [0.77, 1.65] | 0.531 | 1.08 [0.78, 1.48] | 0.645 | 1.11 [0.80, 1.53] | 0.539 | 0.94 [0.68, 1.31] | 0.727 |
| Cerebrovascular disease | 1.15 [0.85, 1.56] | 0.363 | 1.09 [0.85, 1.41] | 0.484 | 1.09 [0.84, 1.42] | 0.497 | 1.12 [0.87, 1.44] | 0.390 |
| Stroke | 1.25 [0.86, 1.83] | 0.240 | 1.09 [0.78, 1.51] | 0.613 | 1.10 [0.78, 1.54] | 0.585 | 1.12 [0.80, 1.56] | 0.509 |
| Hemorrhagic stroke | 1.47 [0.74, 2.90] | 0.269 | 1.08 [0.58, 2.02] | 0.812 | 1.20 [0.64, 2.24] | 0.575 | 1.10 [0.59, 2.08] | 0.758 |
| Ischemic stroke | 1.27 [0.80, 2.02] | 0.303 | 1.06 [0.71, 1.59] | 0.775 | 1.04 [0.69, 1.58] | 0.851 | 1.12 [0.75, 1.68] | 0.588 |
| Myocarditis | 1.61 [0.84, 3.08] | 0.150 | 1.19 [0.65, 2.17] | 0.575 | 1.16 [0.63, 2.16] | 0.630 | 1.14 [0.62, 2.09] | 0.679 |
| Heart failure | 1.84 [1.41, 2.40] | 7.72E-06 | 1.61 [1.28, 2.02] | 5.27E-05 | 1.53 [1.20, 1.95] | 4.92E-04 | 1.51 [1.19, 1.91] | 0.001 |
| Arrhythmias | 1.52 [1.24, 1.85] | 3.59E-05 | 1.34 [1.13, 1.59] | 0.001 | 1.30 [1.09, 1.55] | 0.003 | 1.30 [1.10, 1.55] | 0.002 |
| Atrial fibrillation | 1.57 [1.27, 1.96] | 4.45E-05 | 1.34 [1.11, 1.62] | 0.002 | 1.28 [1.06, 1.56] | 0.013 | 1.30 [1.07, 1.58] | 0.007 |
| Bradyarrhythmia | 1.68 [0.96, 2.96] | 0.070 | 1.49 [0.92, 2.41] | 0.107 | 1.45 [0.88, 2.39] | 0.149 | 1.42 [0.86, 2.32] | 0.168 |
| Other supraventricular arrhythmias | 2.05 [1.16, 3.63] | 0.014 | 1.83 [1.12, 2.99] | 0.016 | 1.88 [1.13, 3.11] | 0.014 | 1.73 [1.05, 2.86] | 0.033 |
| Cardiac arrest and ventricular arrhythmias | 1.15 [0.65, 2.04] | 0.635 | 1.09 [0.67, 1.75] | 0.731 | 1.09 [0.67, 1.78] | 0.738 | 1.03 [0.63, 1.67] | 0.909 |
| Peripheral artery disease | 1.00 [0.54, 1.83] | 0.989 | 1.14 [0.72, 1.82] | 0.577 | 1.04 [0.64, 1.70] | 0.879 | 1.03 [0.64, 1.67] | 0.896 |
| Venous thromboembolism | 1.78 [1.34, 2.37] | 7.89E-05 | 1.74 [1.38, 2.21] | 3.97E-06 | 1.63 [1.27, 2.08] | 1.29E-04 | 1.70 [1.34, 2.16] | 1.47E-05 |
| Pulmonary embolism | 1.31 [0.84, 2.05] | 0.235 | 1.13 [0.76, 1.66] | 0.548 | 1.06 [0.71, 1.59] | 0.772 | 1.08 [0.73, 1.60] | 0.698 |
| Deep vein thrombosis | 2.25 [1.58, 3.18] | 5.46E-06 | 2.27 [1.71, 3.02] | 1.77E-08 | 2.15 [1.59, 2.89] | 5.75E-07 | 2.24 [1.68, 2.99] | 4.56E-08 |

^a^ adjusted for age, sex, Townsend deprivation index, ethnicity, education, body mass index, alcohol drinking, smoking status, physical activity, history of hypertension, diabetes, hyperlipidemia at index date, adherence to cardioprotective diet, and 5-aminosalicylates.

^b^ adjusted for age, sex, Townsend deprivation index, ethnicity, education, body mass index, alcohol drinking, smoking status, physical activity, history of hypertension, diabetes, hyperlipidemia at index date, adherence to cardioprotective diet, and biologic drugs.

^c^ adjusted for age, sex, Townsend deprivation index, ethnicity, education, body mass index, alcohol drinking, smoking status, physical activity, history of hypertension, diabetes, hyperlipidemia at index date, adherence to cardioprotective diet, and immunosuppressants.

^d^ adjusted for age, sex, Townsend deprivation index, ethnicity, education, body mass index, alcohol drinking, smoking status, physical activity, history of hypertension, diabetes, hyperlipidemia at index date, adherence to cardioprotective diet, and glucocorticoids.

CI, confidence interval; CVD, cardiovascular disease; CCI, Charlson comorbidity index; HR, hazard ratio.

# Table S9. Subgroup analysis of the associations between inflammatory bowel disease (IBD) and cardiovascular disease (CVD) outcomes by age, sex, and time interval between index date and recruitment.

|  | **Stratified by age groups** | | | | |
| --- | --- | --- | --- | --- | --- |
|  | **≤50 years old** | | **>50 years old** | |  |
| **CVD outcomes** | **HR (95%CI)** ^a^ | ***P*** | **HR (95%CI)** ^a^ | ***P*** | ***P for interaction*** |
| Any CVD | 1.26 [1.01, 1.56] | 0.037 | 1.35 [1.19, 1.52] | <0.001 | 0.935 |
| Ischemic heart disease | 0.98 [0.69, 1.38] | 0.893 | 1.27 [1.06, 1.53] | 0.011 | 0.350 |
| Myocardial infarction | 1.04 [0.55, 1.95] | 0.906 | 1.10 [0.76, 1.58] | 0.628 | 0.735 |
| Cerebrovascular disease | 1.19 [0.70, 2.01] | 0.514 | 1.05 [0.79, 1.41] | 0.718 | 0.380 |
| Stroke | 0.91 [0.44, 1.90] | 0.806 | 1.12 [0.78, 1.63] | 0.531 | 0.933 |
| Hemorrhagic stroke | 0.74 [0.17, 3.14] | 0.681 | 1.19 [0.59, 2.39] | 0.629 | 0.660 |
| Ischemic stroke | 1.06 [0.45, 2.49] | 0.898 | 1.03 [0.65, 1.64] | 0.889 | 0.469 |
| Myocarditis | 0.85 [0.20, 3.66] | 0.823 | 1.23 [0.63, 2.39] | 0.538 | 0.724 |
| Heart failure | 2.04 [1.28, 3.26] | 0.003 | 1.51 [1.16, 1.97] | 0.002 | 0.305 |
| Arrhythmias | 1.09 [0.73, 1.63] | 0.663 | 1.39 [1.16, 1.68] | <0.001 | 0.324 |
| Atrial fibrillation | 1.05 [0.64, 1.71] | 0.853 | 1.39 [1.13, 1.71] | 0.002 | 0.319 |
| Bradyarrhythmia | 1.69 [0.58, 4.88] | 0.336 | 1.45 [0.84, 2.49] | 0.184 | 0.735 |
| Other supraventricular arrhythmias | 2.61 [1.07, 6.35] | 0.034 | 1.64 [0.91, 2.97] | 0.103 | 0.595 |
| Cardiac arrest and ventricular arrhythmias | 0.70 [0.25, 1.95] | 0.497 | 1.23 [0.72, 2.12] | 0.450 | 0.412 |
| Peripheral artery disease | 2.06 [0.90, 4.72] | 0.089 | 0.93 [0.52, 1.65] | 0.801 | 0.419 |
| Venous thromboembolism | 2.17 [1.42, 3.31] | <0.001 | 1.57 [1.18, 2.09] | 0.002 | 0.291 |
| Pulmonary embolism | 0.97 [0.42, 2.27] | 0.953 | 1.15 [0.74, 1.77] | 0.536 | 0.627 |
| Deep vein thrombosis | 2.76 [1.72, 4.40] | <0.001 | 2.02 [1.41, 2.90] | <0.001 | 0.458 |
|  | **Stratified by sex** | | | | |
|  | **Female** | | **Male** | |  |
| **CVD outcomes** | **HR (95%CI) ^b^** | ***P*** | **HR (95%CI) ^b^** | ***P*** | ***P for interaction*** |
| Any CVD | 1.38 [1.18, 1.63] | <0.001 | 1.28 [1.12, 1.48] | <0.001 | 0.435 |
| Ischemic heart disease | 1.11 [0.84, 1.47] | 0.450 | 1.23 [1.00, 1.51] | 0.045 | 0.653 |
| Myocardial infarction | 0.80 [0.42, 1.54] | 0.505 | 1.19 [0.82, 1.71] | 0.361 | 0.306 |
| Cerebrovascular disease | 1.05 [0.72, 1.53] | 0.790 | 1.09 [0.77, 1.53] | 0.628 | 0.932 |
| Stroke | 1.18 [0.74, 1.90] | 0.482 | 0.97 [0.61, 1.54] | 0.892 | 0.514 |
| Hemorrhagic stroke | 1.57 [0.70, 3.52] | 0.270 | 0.69 [0.25, 1.92] | 0.475 | 0.251 |
| Ischemic stroke | 1.01 [0.55, 1.85] | 0.969 | 1.04 [0.60, 1.81] | 0.884 | 0.916 |
| Myocarditis | 1.19 [0.47, 3.04] | 0.714 | 1.12 [0.51, 2.46] | 0.783 | 0.912 |
| Heart failure | 2.05 [1.48, 2.86] | <0.001 | 1.31 [0.95, 1.81] | 0.102 | 0.059 |
| Arrhythmias | 1.53 [1.18, 1.99] | 0.002 | 1.22 [0.98, 1.52] | 0.071 | 0.194 |
| Atrial fibrillation | 1.47 [1.09, 1.98] | 0.012 | 1.26 [0.99, 1.61] | 0.060 | 0.413 |
| Bradyarrhythmia | 2.33 [1.02, 5.35] | 0.046 | 1.23 [0.68, 2.25] | 0.493 | 0.211 |
| Other supraventricular arrhythmias | 2.00 [1.00, 3.97] | 0.049 | 1.64 [0.80, 3.32] | 0.174 | 0.610 |
| Cardiac arrest and ventricular arrhythmias | 1.23 [0.55, 2.72] | 0.614 | 1.00 [0.55, 1.82] | 0.997 | 0.684 |
| Peripheral artery disease | 0.91 [0.39, 2.13] | 0.824 | 1.28 [0.73, 2.25] | 0.389 | 0.555 |
| Venous thromboembolism | 1.83 [1.29, 2.59] | 0.001 | 1.65 [1.20, 2.28] | 0.002 | 0.558 |
| Pulmonary embolism | 1.12 [0.62, 2.05] | 0.701 | 1.10 [0.66, 1.83] | 0.709 | 0.991 |
| Deep vein thrombosis | 2.25 [1.47, 3.43] | <0.001 | 2.26 [1.54, 3.33] | <0.001 | 0.789 |
|  | **Stratified by time interval between index date and recruitment date** ^d^ | | | | |
|  | **≤5 years** | | **>5 years** | |  |
| **CVD outcomes** | **HR (95%CI) ^c^** | ***P*** | **HR (95%CI) ^c^** | ***P*** | ***P for interaction*** |
| Any CVD | 1.45 [1.26, 1.68] | <0.001 | 1.23 [1.05, 1.43] | 0.010 | 0.134 |
| Ischemic heart disease | 1.27 [1.01, 1.59] | 0.038 | 1.13 [0.89, 1.43] | 0.327 | 0.508 |
| Myocardial infarction | 1.20 [0.78, 1.84] | 0.413 | 0.95 [0.59, 1.53] | 0.836 | 0.478 |
| Cerebrovascular disease | 1.11 [0.78, 1.58] | 0.553 | 1.05 [0.73, 1.51] | 0.785 | 0.854 |
| Stroke | 0.96 [0.59, 1.56] | 0.859 | 1.18 [0.75, 1.84] | 0.477 | 0.456 |
| Hemorrhagic stroke | 0.96 [0.38, 2.41] | 0.929 | 1.19 [0.50, 2.81] | 0.690 | 0.703 |
| Ischemic stroke | 1.10 [0.62, 1.95] | 0.750 | 1.00 [0.56, 1.78] | 0.998 | 0.982 |
| Myocarditis | 1.40 [0.66, 2.94] | 0.378 | 0.86 [0.31, 2.43] | 0.779 | 0.514 |
| Heart failure | 1.95 [1.44, 2.64] | <0.001 | 1.30 [0.91, 1.86] | 0.148 | 0.130 |
| Arrhythmias | 1.67 [1.34, 2.08] | <0.001 | 1.04 [0.80, 1.35] | 0.758 | **0.007** |
| Atrial fibrillation | 1.75 [1.37, 2.23] | <0.001 | 0.96 [0.70, 1.30] | 0.770 | **0.002** |
| Bradyarrhythmia | 1.67 [0.88, 3.17] | 0.114 | 1.28 [0.61, 2.68] | 0.520 | 0.746 |
| Other supraventricular arrhythmias | 1.90 [0.96, 3.75] | 0.065 | 1.79 [0.88, 3.67] | 0.111 | 0.868 |
| Cardiac arrest and ventricular arrhythmias | 1.70 [0.94, 3.09] | 0.080 | 0.61 [0.27, 1.41] | 0.248 | **0.037** |
| Peripheral artery disease | 1.04 [0.54, 2.01] | 0.912 | 1.27 [0.65, 2.48] | 0.482 | 0.640 |
| Venous thromboembolism | 1.76 [1.28, 2.44] | 0.001 | 1.74 [1.23, 2.46] | 0.002 | 0.984 |
| Pulmonary embolism | 1.19 [0.71, 2.00] | 0.516 | 1.03 [0.58, 1.83] | 0.927 | 0.778 |
| Deep vein thrombosis | 2.09 [1.40, 3.13] | <0.001 | 2.55 [1.70, 3.82] | <0.001 | 0.526 |

*CI, confidence interval; CVD, cardiovascular disease; HR, hazard ratio.*

^a^ adjusted for sex, Townsend deprivation index, ethnicity, education, body mass index, alcohol drinking, smoking status, physical activity, history of hypertension, diabetes, hyperlipidemia at index date, and adherence to cardioprotective diet.

^b^ adjusted for age, Townsend deprivation index, ethnicity, education, body mass index, alcohol drinking, smoking status, physical activity, history of hypertension, diabetes, hyperlipidemia at index date, and adherence to cardioprotective diet.

^c^ adjusted for age, sex, Townsend deprivation index, ethnicity, education, body mass index, alcohol drinking, smoking status, physical activity, history of hypertension, diabetes, hyperlipidemia at index date, and adherence to cardioprotective diet.

^d^ Index date is the diagnosis date of inflammatory bowel disease.

Note that some lower or upper 95% CIs are presented as 1.00 due to rounding.

*CI, confidence interval; CVD, cardiovascular disease; CCI, Charlson comorbidity index; HR, hazard ratio.*

# Table S10. Association between Crohn’s disease (CD), Ulcerative colitis (UC), and IBD-unclassified (IBD-U) and the risk of cardiovascular disease (CVD) outcomes with further adjustment for history of CVD, metabolic status, and Charlson comorbidity index (CCI).

|  |  | **Further adjusted for family history of CVD ^a^** | | **Further adjusted for metabolic status ^b^** | | **Further adjusted for CCI ^c^** | |
| --- | --- | --- | --- | --- | --- | --- | --- |
|  | **CVD outcomes** | **HR (95%CI)** | ***P*** | **HR (95%CI)** | ***P*** | **HR (95%CI)** | ***P*** |
| CD | Any CVD | 1.53 [1.23, 1.90] | 1.65E-04 | 1.53 [1.22, 1.90] | 1.67E-04 | 1.53 [1.22, 1.90] | 1.68E-04 |
|  | Ischemic heart disease | 1.28 [0.89, 1.82] | 0.183 | 1.28 [0.90, 1.83] | 0.175 | 1.29 [0.90, 1.84] | 0.165 |
|  | Myocardial infarction | 1.21 [0.64, 2.31] | 0.557 | 1.20 [0.63, 2.29] | 0.571 | 1.22 [0.64, 2.32] | 0.543 |
|  | Cerebrovascular disease | 1.68 [1.01, 2.79] | 0.046 | 1.70 [1.02, 2.84] | 0.040 | 1.70 [1.02, 2.83] | 0.040 |
|  | Stroke | 1.72 [0.89, 3.31] | 0.105 | 1.74 [0.90, 3.36] | 0.097 | 1.75 [0.91, 3.37] | 0.095 |
|  | Hemorrhagic stroke | 1.55 [0.52, 4.62] | 0.436 | 1.56 [0.52, 4.66] | 0.426 | 1.66 [0.55, 4.97] | 0.366 |
|  | Ischemic stroke | 1.99 [0.87, 4.56] | 0.102 | 2.05 [0.90, 4.68] | 0.089 | 2.08 [0.91, 4.75] | 0.083 |
|  | Myocarditis | 0.60 [0.14, 2.57] | 0.492 | 0.60 [0.14, 2.56] | 0.488 | 0.61 [0.14, 2.60] | 0.505 |
|  | Heart failure | 1.88 [1.20, 2.96] | 0.006 | 1.92 [1.22, 3.01] | 0.005 | 1.90 [1.21, 2.98] | 0.005 |
|  | Arrhythmias | 1.74 [1.25, 2.43] | 0.001 | 1.74 [1.25, 2.43] | 0.001 | 1.76 [1.26, 2.45] | 0.001 |
|  | Atrial fibrillation | 1.79 [1.23, 2.62] | 0.003 | 1.80 [1.23, 2.63] | 0.002 | 1.82 [1.25, 2.67] | 0.002 |
|  | Bradyarrhythmia | 1.61 [0.55, 4.75] | 0.384 | 1.64 [0.56, 4.79] | 0.370 | 1.66 [0.56, 4.89] | 0.358 |
|  | Other supraventricular arrhythmias | 2.93 [1.30, 6.61] | 0.009 | 2.89 [1.28, 6.53] | 0.010 | 2.88 [1.28, 6.50] | 0.011 |
|  | Cardiac arrest and ventricular arrhythmias | 0.69 [0.21, 2.29] | 0.549 | 0.69 [0.21, 2.26] | 0.537 | 0.70 [0.21, 2.31] | 0.560 |
|  | Peripheral artery disease | 1.08 [0.41, 2.87] | 0.874 | 1.12 [0.42, 2.97] | 0.821 | 1.11 [0.42, 2.95] | 0.837 |
|  | Venous thromboembolism | 1.45 [0.85, 2.48] | 0.174 | 1.47 [0.86, 2.50] | 0.163 | 1.45 [0.85, 2.48] | 0.173 |
|  | Pulmonary embolism | 1.07 [0.46, 2.52] | 0.871 | 1.07 [0.46, 2.52] | 0.873 | 1.08 [0.46, 2.53] | 0.866 |
|  | Deep vein thrombosis | 1.64 [0.85, 3.17] | 0.139 | 1.67 [0.87, 3.23] | 0.124 | 1.65 [0.85, 3.18] | 0.136 |
| UC | Any CVD | 1.21 [1.06, 1.39] | 0.005 | 1.21 [1.05, 1.38] | 0.007 | 1.19 [1.04, 1.36] | 0.012 |
|  | Ischemic heart disease | 1.05 [0.85, 1.30] | 0.651 | 1.04 [0.84, 1.29] | 0.692 | 1.03 [0.83, 1.28] | 0.772 |
|  | Myocardial infarction | 0.84 [0.55, 1.29] | 0.423 | 0.84 [0.55, 1.29] | 0.418 | 0.82 [0.54, 1.27] | 0.380 |
|  | Cerebrovascular disease | 0.95 [0.68, 1.31] | 0.743 | 0.94 [0.68, 1.30] | 0.714 | 0.93 [0.67, 1.29] | 0.667 |
|  | Stroke | 0.99 [0.65, 1.51] | 0.960 | 0.99 [0.65, 1.51] | 0.948 | 0.98 [0.64, 1.50] | 0.939 |
|  | Hemorrhagic stroke | 0.74 [0.30, 1.86] | 0.526 | 0.75 [0.30, 1.88] | 0.539 | 0.78 [0.31, 1.95] | 0.597 |
|  | Ischemic stroke | 0.96 [0.57, 1.60] | 0.862 | 0.95 [0.57, 1.60] | 0.851 | 0.94 [0.56, 1.58] | 0.815 |
|  | Myocarditis | 1.56 [0.77, 3.16] | 0.217 | 1.52 [0.75, 3.07] | 0.246 | 1.54 [0.76, 3.13] | 0.228 |
|  | Heart failure | 1.39 [1.03, 1.89] | 0.034 | 1.38 [1.02, 1.87] | 0.038 | 1.35 [1.00, 1.84] | 0.053 |
|  | Arrhythmias | 1.31 [1.06, 1.62] | 0.011 | 1.31 [1.06, 1.61] | 0.012 | 1.28 [1.04, 1.58] | 0.022 |
|  | Atrial fibrillation | 1.30 [1.03, 1.64] | 0.028 | 1.29 [1.02, 1.63] | 0.031 | 1.26 [0.99, 1.59] | 0.055 |
|  | Bradyarrhythmia | 1.47 [0.82, 2.64] | 0.200 | 1.49 [0.83, 2.69] | 0.181 | 1.43 [0.80, 2.58] | 0.229 |
|  | Other supraventricular arrhythmias | 1.61 [0.82, 3.16] | 0.165 | 1.60 [0.81, 3.13] | 0.173 | 1.59 [0.81, 3.13] | 0.176 |
|  | Cardiac arrest and ventricular arrhythmias | 1.24 [0.71, 2.18] | 0.447 | 1.24 [0.71, 2.18] | 0.447 | 1.23 [0.70, 2.16] | 0.469 |
|  | Peripheral artery disease | 0.93 [0.48, 1.78] | 0.817 | 0.93 [0.48, 1.78] | 0.818 | 0.88 [0.46, 1.70] | 0.711 |
|  | Venous thromboembolism | 1.75 [1.31, 2.34] | 1.69E-04 | 1.74 [1.30, 2.33] | 1.85E-04 | 1.71 [1.28, 2.30] | 3.01E-04 |
|  | Pulmonary embolism | 1.18 [0.74, 1.88] | 0.496 | 1.18 [0.74, 1.88] | 0.492 | 1.11 [0.70, 1.78] | 0.656 |
|  | Deep vein thrombosis | 2.27 [1.59, 3.24] | 5.73E-06 | 2.26 [1.58, 3.22] | 6.79E-06 | 2.30 [1.61, 3.28] | 4.35E-06 |
| IBD-U | Any CVD | 1.62 [1.22, 2.14] | 0.001 | 1.65 [1.24, 2.18] | 4.82E-04 | 1.62 [1.23, 2.15] | 0.001 |
|  | Ischemic heart disease | 1.78 [1.21, 2.62] | 0.003 | 1.80 [1.23, 2.65] | 0.003 | 1.78 [1.21, 2.62] | 0.003 |
|  | Myocardial infarction | 1.93 [0.92, 4.04] | 0.082 | 1.98 [0.94, 4.16] | 0.072 | 2.04 [0.97, 4.28] | 0.060 |
|  | Cerebrovascular disease | 1.09 [0.56, 2.12] | 0.798 | 1.08 [0.55, 2.10] | 0.821 | 1.09 [0.56, 2.13] | 0.791 |
|  | Stroke | 0.81 [0.32, 2.05] | 0.654 | 0.80 [0.32, 2.03] | 0.641 | 0.81 [0.32, 2.05] | 0.656 |
|  | Hemorrhagic stroke | 2.09 [0.47, 9.36] | 0.336 | 1.85 [0.41, 8.28] | 0.423 | 2.10 [0.47, 9.40] | 0.332 |
|  | Ischemic stroke | 0.71 [0.22, 2.31] | 0.565 | 0.72 [0.22, 2.35] | 0.587 | 0.71 [0.22, 2.32] | 0.571 |
|  | Myocarditis | 1.09 [0.14, 8.50] | 0.936 | 1.08 [0.14, 8.45] | 0.940 | 1.10 [0.14, 8.60] | 0.927 |
|  | Heart failure | 2.07 [1.14, 3.75] | 0.016 | 2.09 [1.15, 3.79] | 0.015 | 2.04 [1.12, 3.69] | 0.019 |
|  | Arrhythmias | 0.83 [0.47, 1.48] | 0.534 | 0.85 [0.48, 1.51] | 0.576 | 0.84 [0.47, 1.49] | 0.542 |
|  | Atrial fibrillation | 0.92 [0.49, 1.73] | 0.804 | 0.93 [0.49, 1.73] | 0.809 | 0.93 [0.50, 1.74] | 0.818 |
|  | Bradyarrhythmia | 1.20 [0.28, 5.21] | 0.808 | 1.28 [0.29, 5.60] | 0.739 | 1.17 [0.27, 5.10] | 0.834 |
|  | Other supraventricular arrhythmias | 0.79 [0.10, 5.97] | 0.817 | 0.76 [0.10, 5.77] | 0.792 | 0.78 [0.10, 5.90] | 0.809 |
|  | Cardiac arrest and ventricular arrhythmias | 0.85 [0.19, 3.74] | 0.833 | 0.87 [0.20, 3.83] | 0.851 | 0.85 [0.19, 3.79] | 0.834 |
|  | Peripheral artery disease | 1.70 [0.60, 4.80] | 0.319 | 1.69 [0.60, 4.77] | 0.320 | 1.71 [0.61, 4.83] | 0.309 |
|  | Venous thromboembolism | 2.28 [1.20, 4.32] | 0.012 | 2.23 [1.18, 4.24] | 0.014 | 2.17 [1.14, 4.14] | 0.018 |
|  | Pulmonary embolism | 0.83 [0.25, 2.78] | 0.762 | 0.82 [0.24, 2.73] | 0.743 | 0.84 [0.25, 2.81] | 0.777 |
|  | Deep vein thrombosis | 4.14 [1.96, 8.74] | 1.93E-04 | 4.07 [1.93, 8.60] | 2.37E-04 | 3.80 [1.78, 8.13] | 0.001 |

^a^ adjusted for age, sex, Townsend deprivation index, ethnicity, education, body mass index, alcohol drinking, smoking status, physical activity, history of hypertension, diabetes, hyperlipidemia at index date, adherence to cardioprotective diet, and family history of CVD.

^b^ adjusted for age, sex, Townsend deprivation index, ethnicity, education, body mass index, alcohol drinking, smoking status, physical activity, history of hypertension, diabetes, hyperlipidemia at index date, adherence to cardioprotective diet, and metabolic status.

^c^ adjusted for age, sex, Townsend deprivation index, ethnicity, education, body mass index, alcohol drinking, smoking status, physical activity, history of hypertension, diabetes, hyperlipidemia at index date, adherence to cardioprotective diet, and Charlson comorbidity index.

Note that some lower or upper 95% CIs are presented as 1.00 due to rounding.

*CI, confidence interval; CVD, cardiovascular disease; CCI, Charlson comorbidity index; HR, hazard ratio*

# Table S11. Association between Crohn’s disease (CD), Ulcerative colitis (UC), and IBD-unclassified (IBD-U) and the risk of cardiovascular disease (CVD) outcomes with further adjustment for 5-aminosalicylates, biologic drugs, immunosuppressants, and glucocorticoids

|  |  | **Further adjusted for use of 5-aminosalicylates ^a^** | | **Further adjusted for use of biologic drugs ^b^** | | **Further adjusted for use of immunosuppressants ^c^** | | **Further adjusted for use of glucocorticoids ^d^** | |
| --- | --- | --- | --- | --- | --- | --- | --- | --- | --- |
|  | **CVD outcomes** | **HR (95%CI)** | ***P*** | **HR (95%CI)** | ***P*** | **HR (95%CI)** | ***P*** | **HR (95%CI)** | ***P*** |
| CD | Any CVD | 1.75 [1.36, 2.24] | 9.89E-06 | 1.54 [1.23, 1.92] | 1.33E-04 | 1.50 [1.19, 1.88] | 0.001 | 1.50 [1.20, 1.88] | 3.52E-04 |
|  | Ischemic heart disease | 1.60 [1.08, 2.37] | 0.018 | 1.29 [0.90, 1.85] | 0.159 | 1.32 [0.92, 1.91] | 0.134 | 1.29 [0.89, 1.85] | 0.174 |
|  | Myocardial infarction | 1.18 [0.55, 2.54] | 0.679 | 1.23 [0.65, 2.34] | 0.531 | 1.22 [0.62, 2.37] | 0.565 | 1.14 [0.60, 2.20] | 0.686 |
|  | Cerebrovascular disease | 1.83 [1.03, 3.26] | 0.040 | 1.71 [1.03, 2.85] | 0.038 | 1.76 [1.04, 2.96] | 0.034 | 1.71 [1.02, 2.86] | 0.040 |
|  | Stroke | 1.94 [0.93, 4.07] | 0.079 | 1.75 [0.91, 3.38] | 0.094 | 1.97 [1.02, 3.79] | 0.043 | 1.69 [0.87, 3.28] | 0.124 |
|  | Hemorrhagic stroke | 1.81 [0.54, 6.07] | 0.339 | 1.58 [0.53, 4.73] | 0.415 | 1.79 [0.60, 5.35] | 0.295 | 1.45 [0.48, 4.44] | 0.510 |
|  | Ischemic stroke | 2.17 [0.84, 5.57] | 0.109 | 2.05 [0.90, 4.70] | 0.088 | 2.27 [0.99, 5.18] | 0.052 | 2.14 [0.94, 4.90] | 0.071 |
|  | Myocarditis | 0.86 [0.20, 3.68] | 0.840 | 0.60 [0.14, 2.58] | 0.496 | 0.66 [0.15, 2.83] | 0.577 | 0.64 [0.15, 2.72] | 0.541 |
|  | Heart failure | 1.98 [1.17, 3.34] | 0.011 | 1.92 [1.22, 3.01] | 0.005 | 1.89 [1.18, 3.01] | 0.008 | 1.82 [1.15, 2.88] | 0.011 |
|  | Arrhythmias | 1.83 [1.26, 2.68] | 0.002 | 1.75 [1.25, 2.44] | 0.001 | 1.68 [1.19, 2.38] | 0.003 | 1.72 [1.23, 2.40] | 0.002 |
|  | Atrial fibrillation | 1.87 [1.21, 2.89] | 0.005 | 1.80 [1.23, 2.63] | 0.002 | 1.70 [1.14, 2.53] | 0.009 | 1.75 [1.19, 2.57] | 0.005 |
|  | Bradyarrhythmia | 1.72 [0.52, 5.72] | 0.375 | 1.64 [0.56, 4.80] | 0.368 | 1.91 [0.65, 5.58] | 0.237 | 1.71 [0.58, 5.02] | 0.329 |
|  | Other supraventricular arrhythmias | 2.75 [1.05, 7.20] | 0.039 | 2.89 [1.28, 6.52] | 0.010 | 3.24 [1.44, 7.32] | 0.005 | 2.79 [1.22, 6.39] | 0.015 |
|  | Cardiac arrest and ventricular arrhythmias | 1.08 [0.33, 3.56] | 0.893 | 0.70 [0.21, 2.32] | 0.565 | 0.65 [0.19, 2.26] | 0.501 | 0.66 [0.20, 2.22] | 0.507 |
|  | Peripheral artery disease | 1.07 [0.32, 3.55] | 0.911 | 1.11 [0.42, 2.96] | 0.832 | 0.94 [0.34, 2.62] | 0.906 | 1.01 [0.37, 2.72] | 0.989 |
|  | Venous thromboembolism | 1.81 [1.02, 3.23] | 0.044 | 1.46 [0.86, 2.50] | 0.163 | 1.30 [0.74, 2.30] | 0.361 | 1.40 [0.81, 2.40] | 0.230 |
|  | Pulmonary embolism | 1.63 [0.69, 3.83] | 0.261 | 1.09 [0.46, 2.55] | 0.850 | 0.98 [0.40, 2.41] | 0.971 | 0.98 [0.41, 2.34] | 0.956 |
|  | Deep vein thrombosis | 1.87 [0.89, 3.90] | 0.096 | 1.66 [0.86, 3.20] | 0.130 | 1.50 [0.75, 3.00] | 0.252 | 1.65 [0.85, 3.20] | 0.138 |
|  |  | **Further adjusted for use of 5-aminosalicylates ^a^** | | **Further adjusted for use of biologic drugs ^b^** | | **Further adjusted for use of immunosuppressants ^c^** | | **Further adjusted for use of glucocorticoids ^d^** | |
|  | **CVD outcomes** | **HR (95%CI)** | ***P*** | **HR (95%CI)** | ***P*** | **HR (95%CI)** | ***P*** | **HR (95%CI)** | ***P*** |
| UC | Any CVD | 1.37 [1.16, 1.61] | 1.35E-04 | 1.21 [1.05, 1.38] | 0.007 | 1.16 [1.01, 1.34] | 0.038 | 1.16 [1.01, 1.33] | 0.034 |
|  | Ischemic heart disease | 1.25 [0.98, 1.60] | 0.076 | 1.04 [0.84, 1.28] | 0.717 | 0.99 [0.79, 1.24] | 0.937 | 0.98 [0.79, 1.21] | 0.831 |
|  | Myocardial infarction | 0.99 [0.61, 1.62] | 0.970 | 0.83 [0.54, 1.28] | 0.402 | 0.87 [0.56, 1.35] | 0.533 | 0.71 [0.46, 1.11] | 0.136 |
|  | Cerebrovascular disease | 0.98 [0.66, 1.45] | 0.914 | 0.94 [0.68, 1.30] | 0.709 | 0.93 [0.66, 1.30] | 0.663 | 0.97 [0.70, 1.34] | 0.843 |
|  | Stroke | 1.13 [0.69, 1.85] | 0.631 | 0.99 [0.65, 1.50] | 0.946 | 0.96 [0.62, 1.48] | 0.842 | 1.03 [0.67, 1.57] | 0.903 |
|  | Hemorrhagic stroke | 1.27 [0.51, 3.17] | 0.612 | 0.75 [0.30, 1.88] | 0.541 | 0.83 [0.33, 2.06] | 0.682 | 0.80 [0.32, 2.00] | 0.636 |
|  | Ischemic stroke | 1.13 [0.62, 2.05] | 0.688 | 0.95 [0.57, 1.59] | 0.848 | 0.90 [0.53, 1.53] | 0.695 | 1.00 [0.60, 1.68] | 0.989 |
|  | Myocarditis | 2.02 [0.93, 4.41] | 0.077 | 1.52 [0.75, 3.08] | 0.245 | 1.40 [0.67, 2.93] | 0.375 | 1.39 [0.68, 2.87] | 0.366 |
|  | Heart failure | 1.70 [1.20, 2.41] | 0.003 | 1.38 [1.02, 1.87] | 0.039 | 1.26 [0.92, 1.74] | 0.155 | 1.28 [0.93, 1.74] | 0.126 |
|  | Arrhythmias | 1.49 [1.16, 1.91] | 0.002 | 1.30 [1.06, 1.61] | 0.013 | 1.27 [1.02, 1.57] | 0.034 | 1.26 [1.02, 1.56] | 0.030 |
|  | Atrial fibrillation | 1.52 [1.15, 2.00] | 0.003 | 1.29 [1.02, 1.63] | 0.032 | 1.24 [0.97, 1.58] | 0.087 | 1.25 [0.99, 1.59] | 0.061 |
|  | Bradyarrhythmia | 1.59 [0.78, 3.24] | 0.203 | 1.48 [0.83, 2.67] | 0.187 | 1.34 [0.72, 2.49] | 0.361 | 1.35 [0.74, 2.47] | 0.323 |
|  | Other supraventricular arrhythmias | 2.21 [1.06, 4.62] | 0.035 | 1.60 [0.82, 3.15] | 0.169 | 1.52 [0.75, 3.08] | 0.248 | 1.54 [0.77, 3.06] | 0.220 |
|  | Cardiac arrest and ventricular arrhythmias | 1.36 [0.70, 2.66] | 0.363 | 1.24 [0.71, 2.16] | 0.458 | 1.30 [0.74, 2.29] | 0.369 | 1.19 [0.67, 2.11] | 0.548 |
|  | Peripheral artery disease | 0.85 [0.37, 1.96] | 0.700 | 0.91 [0.48, 1.76] | 0.790 | 0.82 [0.41, 1.64] | 0.577 | 0.82 [0.42, 1.62] | 0.571 |
|  | Venous thromboembolism | 1.78 [1.24, 2.55] | 0.002 | 1.74 [1.30, 2.33] | 1.80E-04 | 1.69 [1.25, 2.29] | 0.001 | 1.71 [1.27, 2.30] | 3.54E-04 |
|  | Pulmonary embolism | 1.37 [0.79, 2.39] | 0.260 | 1.17 [0.73, 1.87] | 0.506 | 1.16 [0.72, 1.88] | 0.542 | 1.16 [0.73, 1.87] | 0.525 |
|  | Deep vein thrombosis | 2.20 [1.41, 3.44] | 0.001 | 2.26 [1.59, 3.22] | 6.34E-06 | 2.19 [1.51, 3.18] | 3.28E-05 | 2.19 [1.53, 3.14] | 2.06E-05 |
|  |  | **Further adjusted for use of 5-aminosalicylates ^a^** | | **Further adjusted for use of immunosuppressants ^c^** | | **Further adjusted for use of glucocorticoids ^d^** | |  |  |
|  | **CVD outcomes** | **HR (95%CI)** | ***P*** | **HR (95%CI)** | ***P*** | **HR (95%CI)** | ***P*** |  |  |
| IBD-U | Any CVD | 1.64 [1.16, 2.30] | 0.005 | 1.59 [1.19, 2.13] | 0.002 | 1.62 [1.21, 2.15] | 0.001 |  |  |
|  | Ischemic heart disease | 1.56 [0.96, 2.53] | 0.070 | 1.82 [1.23, 2.71] | 0.003 | 1.78 [1.20, 2.64] | 0.004 |  |  |
|  | Myocardial infarction | 1.39 [0.51, 3.80] | 0.518 | 2.00 [0.94, 4.27] | 0.072 | 1.65 [0.75, 3.60] | 0.210 |  |  |
|  | Cerebrovascular disease | 1.14 [0.50, 2.60] | 0.751 | 1.11 [0.56, 2.19] | 0.757 | 1.14 [0.58, 2.23] | 0.708 |  |  |
|  | Stroke | 1.12 [0.40, 3.12] | 0.823 | 0.82 [0.32, 2.12] | 0.682 | 0.87 [0.35, 2.21] | 0.776 |  |  |
|  | Hemorrhagic stroke | 1.59 [0.35, 7.10] | 0.546 | 2.49 [0.56, 11.14] | 0.233 | 2.19 [0.49, 9.81] | 0.305 |  |  |
|  | Ischemic stroke | 1.11 [0.34, 3.62] | 0.868 | 0.68 [0.20, 2.33] | 0.543 | 0.77 [0.24, 2.51] | 0.662 |  |  |
|  | Myocarditis | 1.87 [0.24, 14.60] | 0.551 | 1.12 [0.14, 8.72] | 0.916 | 1.15 [0.15, 8.98] | 0.894 |  |  |
|  | Heart failure | 2.14 [1.06, 4.32] | 0.035 | 2.07 [1.13, 3.79] | 0.018 | 2.04 [1.12, 3.72] | 0.020 |  |  |
|  | Arrhythmias | 1.08 [0.57, 2.06] | 0.810 | 0.82 [0.46, 1.49] | 0.519 | 0.79 [0.44, 1.43] | 0.444 |  |  |
|  | Atrial fibrillation | 1.42 [0.74, 2.74] | 0.292 | 0.91 [0.48, 1.74] | 0.783 | 0.88 [0.46, 1.67] | 0.689 |  |  |
|  | Bradyarrhythmia | 1.77 [0.41, 7.68] | 0.447 | 1.32 [0.30, 5.73] | 0.713 | 1.29 [0.30, 5.58] | 0.735 |  |  |
|  | Other supraventricular arrhythmias | 0.16 [0.02, 1.20] | 0.074 | 0.87 [0.12, 6.63] | 0.897 | 0.58 [0.08, 4.36] | 0.593 |  |  |
|  | Cardiac arrest and ventricular arrhythmias | 0.22 [0.02, 2.83] | 0.245 | 0.79 [0.18, 3.53] | 0.754 | 0.75 [0.17, 3.42] | 0.714 |  |  |
|  | Peripheral artery disease | 1.24 [0.30, 5.18] | 0.768 | 2.05 [0.71, 5.97] | 0.187 | 1.83 [0.63, 5.34] | 0.266 |  |  |
|  | Venous thromboembolism | 1.82 [0.78, 4.23] | 0.163 | 1.94 [0.98, 3.83] | 0.058 | 2.19 [1.14, 4.23] | 0.019 |  |  |
|  | Pulmonary embolism | 0.49 [0.08, 2.89] | 0.428 | 0.74 [0.22, 2.52] | 0.628 | 0.69 [0.20, 2.41] | 0.565 |  |  |
|  | Deep vein thrombosis | 4.07 [1.65, 10.04] | 0.002 | 3.71 [1.65, 8.33] | 0.002 | 4.47 [2.12, 9.42] | 8.56E-05 |  |  |

Due to the power issue, the adjustment for biologic drugs were not performed for IBD-U.

^a^ adjusted for age, sex, Townsend deprivation index, ethnicity, education, body mass index, alcohol drinking, smoking status, physical activity, history of hypertension, diabetes, hyperlipidemia at index date, adherence to cardioprotective diet, and 5-aminosalicylates.

^b^ adjusted for age, sex, Townsend deprivation index, ethnicity, education, body mass index, alcohol drinking, smoking status, physical activity, history of hypertension, diabetes, hyperlipidemia at index date, adherence to cardioprotective diet, and biologic drugs.

^c^ adjusted for age, sex, Townsend deprivation index, ethnicity, education, body mass index, alcohol drinking, smoking status, physical activity, history of hypertension, diabetes, hyperlipidemia at index date, adherence to cardioprotective diet, and immunosuppressants.

^d^ adjusted for age, sex, Townsend deprivation index, ethnicity, education, body mass index, alcohol drinking, smoking status, physical activity, history of hypertension, diabetes, hyperlipidemia at index date, adherence to cardioprotective diet, and glucocorticoids.

Due to the power issue, the adjustment for biologic drugs were not performed for IBD-U.

CI, confidence interval; CVD, cardiovascular disease; CCI, Charlson comorbidity index; HR, hazard ratio.

# Table S12. Association between stringent defined IBD subtypes and the risk of cardiovascular disease (CVD) outcomes.

|  | **CVD outcomes** | **Patient IR** | **Reference IR** | **IR difference (95%CI)** | **HR (95%CI)** | ***P*** ^a^ |
| --- | --- | --- | --- | --- | --- | --- |
| CD  (N=300) | Any CVD | 85 (17.65) | 544 (10.67) | 6.98 (3.15,10.8) | **1.53 [1.21, 1.93]** | **3.39E-04**** |
|  | Ischemic heart disease | 31 (6.1) | 235 (4.46) | 1.64 (-0.57,3.85) | 1.23 [0.84, 1.81] | 0.282 |
|  | Myocardial infarction | 10 (1.91) | 73 (1.36) | 0.55 (-0.67,1.77) | 1.13 [0.58, 2.22] | 0.720 |
|  | Cerebrovascular disease | 16 (3.06) | 95 (1.77) | 1.29 (-0.25,2.83) | **1.76 [1.02, 3.02]** | **0.041** |
|  | Stroke | 10 (1.9) | 56 (1.04) | 0.86 (-0.34,2.07) | 1.84 [0.92, 3.67] | 0.082 |
|  | Hemorrhagic stroke | 3 (0.57) | 19 (0.35) | 0.21 (-0.44,0.87) | 1.35 [0.39, 4.73] | 0.637 |
|  | Ischemic stroke | 6 (1.14) | 35 (0.65) | 0.49 (-0.44,1.43) | 1.91 [0.78, 4.64] | 0.154 |
|  | Myocarditis | 2 (0.38) | 25 (0.46) | -0.09 (-0.64,0.47) | 0.70 [0.16, 3.07] | 0.642 |
|  | Heart failure | 20 (3.83) | 100 (1.86) | 1.97 (0.26,3.69) | **1.64 [1.00, 2.69]** | **0.050** |
|  | Arrhythmias | 38 (7.36) | 212 (3.99) | 3.37 (0.98,5.76) | **1.75 [1.23, 2.49]** | **0.002**** |
|  | Atrial fibrillation | 29 (5.58) | 159 (2.98) | 2.61 (0.53,4.69) | **1.87 [1.25, 2.80]** | **0.002**** |
|  | Bradyarrhythmia | 4 (0.75) | 25 (0.46) | 0.29 (-0.47,1.05) | 1.68 [0.56, 4.99] | 0.355 |
|  | Other supraventricular arrhythmias | 7 (1.33) | 26 (0.48) | 0.84 (-0.15,1.84) | **2.63 [1.10, 6.29]** | **0.030** |
|  | Cardiac arrest and ventricular arrhythmias | 2 (0.38) | 36 (0.67) | -0.29 (-0.86,0.28) | 0.46 [0.11, 1.94] | 0.289 |
|  | Peripheral artery disease | 5 (0.95) | 29 (0.54) | 0.41 (-0.44,1.27) | 1.34 [0.50, 3.61] | 0.566 |
|  | Venous thromboembolism | 14 (2.67) | 89 (1.66) | 1.01 (-0.42,2.45) | 1.39 [0.78, 2.47] | 0.262 |
|  | Pulmonary embolism | 5 (0.94) | 48 (0.89) | 0.05 (-0.81,0.92) | 0.95 [0.38, 2.43] | 0.923 |
|  | Deep vein thrombosis | 10 (1.9) | 52 (0.96) | 0.94 (-0.27,2.15) | 1.66 [0.83, 3.33] | 0.156 |
| UC  (N=886) | Any CVD | 218 (14.53) | 1821 (11.92) | 2.61 (0.62,4.6) | **1.20 [1.05, 1.39]** | **0.010** |
|  | Ischemic heart disease | 90 (5.72) | 829 (5.24) | 0.48 (-0.75,1.71) | 1.06 [0.85, 1.32] | 0.589 |
|  | Myocardial infarction | 22 (1.36) | 244 (1.51) | -0.14 (-0.74,0.46) | 0.86 [0.55, 1.33] | 0.501 |
|  | Cerebrovascular disease | 36 (2.24) | 382 (2.36) | -0.13 (-0.89,0.64) | 0.90 [0.64, 1.28] | 0.569 |
|  | Stroke | 23 (1.43) | 219 (1.35) | 0.08 (-0.53,0.68) | 1.02 [0.66, 1.57] | 0.932 |
|  | Hemorrhagic stroke | 5 (0.31) | 59 (0.36) | -0.05 (-0.34,0.23) | 0.79 [0.32, 1.98] | 0.614 |
|  | Ischemic stroke | 15 (0.93) | 155 (0.95) | -0.03 (-0.52,0.46) | 0.96 [0.57, 1.64] | 0.891 |
|  | Myocarditis | 9 (0.56) | 57 (0.35) | 0.21 (-0.17,0.58) | 1.62 [0.80, 3.29] | 0.183 |
|  | Heart failure | 42 (2.63) | 312 (1.93) | 0.7 (-0.13,1.52) | 1.31 [0.95, 1.82] | 0.102 |
|  | Arrhythmias | 90 (5.67) | 702 (4.4) | 1.27 (0.06,2.49) | **1.26 [1.01, 1.57]** | **0.041** |
|  | Atrial fibrillation | 73 (4.58) | 565 (3.52) | 1.06 (-0.03,2.14) | 1.26 [0.99, 1.61] | 0.065 |
|  | Bradyarrhythmia | 11 (0.68) | 85 (0.52) | 0.16 (-0.26,0.57) | 1.31 [0.70, 2.46] | 0.404 |
|  | Other supraventricular arrhythmias | 8 (0.49) | 55 (0.34) | 0.16 (-0.2,0.51) | 1.46 [0.69, 3.09] | 0.318 |
|  | Cardiac arrest and ventricular arrhythmias | 13 (0.8) | 100 (0.61) | 0.19 (-0.27,0.64) | 1.29 [0.72, 2.31] | 0.392 |
|  | Peripheral artery disease | 9 (0.55) | 93 (0.57) | -0.02 (-0.4,0.36) | 0.89 [0.45, 1.78] | 0.749 |
|  | Venous thromboembolism | 50 (3.12) | 287 (1.77) | 1.35 (0.46,2.23) | **1.77 [1.31, 2.40]** | **2.21E-04**** |
|  | Pulmonary embolism | 18 (1.11) | 154 (0.95) | 0.16 (-0.37,0.7) | 1.16 [0.71, 1.90] | 0.551 |
|  | Deep vein thrombosis | 36 (2.24) | 158 (0.97) | 1.26 (0.52,2.01) | **2.34 [1.62, 3.37]** | **5.12E-06**** |
| IBD-U  (N=308) | Any CVD | 88 (17.67) | 595 (11.33) | 6.34 (2.57,10.11) | **1.51 [1.20, 1.90]** | **3.66E-04**** |
|  | Ischemic heart disease | 42 (7.95) | 261 (4.8) | 3.14 (0.68,5.61) | **1.52 [1.09, 2.11]** | **0.013** |
|  | Myocardial infarction | 11 (2) | 62 (1.12) | 0.89 (-0.33,2.1) | 1.60 [0.83, 3.06] | 0.160 |
|  | Cerebrovascular disease | 16 (2.92) | 122 (2.2) | 0.72 (-0.76,2.2) | 1.27 [0.75, 2.16] | 0.369 |
|  | Stroke | 7 (1.27) | 80 (1.44) | -0.17 (-1.16,0.82) | 0.86 [0.39, 1.88] | 0.708 |
|  | Hemorrhagic stroke | 3 (0.54) | 18 (0.32) | 0.22 (-0.41,0.85) | 2.04 [0.60, 6.93] | 0.253 |
|  | Ischemic stroke | 5 (0.9) | 55 (0.99) | -0.09 (-0.92,0.75) | 0.92 [0.36, 2.31] | 0.853 |
|  | Myocarditis | 1 (0.18) | 19 (0.34) | -0.16 (-0.55,0.22) | 0.47 [0.06, 3.53] | 0.461 |
|  | Heart failure | 24 (4.44) | 99 (1.79) | 2.65 (0.84,4.46) | **2.31 [1.46, 3.64]** | **3.15E-04**** |
|  | Arrhythmias | 28 (5.17) | 232 (4.24) | 0.93 (-1.05,2.92) | 1.15 [0.78, 1.72] | 0.477 |
|  | Atrial fibrillation | 22 (4.03) | 184 (3.35) | 0.68 (-1.07,2.43) | 1.15 [0.73, 1.79] | 0.552 |
|  | Bradyarrhythmia | 4 (0.72) | 26 (0.47) | 0.26 (-0.47,0.99) | 1.81 [0.62, 5.27] | 0.278 |
|  | Other supraventricular arrhythmias | 4 (0.72) | 24 (0.43) | 0.29 (-0.44,1.02) | 1.76 [0.60, 5.16] | 0.302 |
|  | Cardiac arrest and ventricular arrhythmias | 4 (0.72) | 32 (0.57) | 0.15 (-0.59,0.88) | 1.07 [0.37, 3.05] | 0.905 |
|  | Peripheral artery disease | 6 (1.09) | 35 (0.63) | 0.46 (-0.44,1.35) | 1.45 [0.59, 3.57] | 0.418 |
|  | Venous thromboembolism | 18 (3.28) | 91 (1.64) | 1.64 (0.09,3.19) | **1.99 [1.19, 3.32]** | **0.008** |
|  | Pulmonary embolism | 6 (1.08) | 51 (0.92) | 0.17 (-0.73,1.07) | 1.09 [0.46, 2.58] | 0.838 |
|  | Deep vein thrombosis | 13 (2.36) | 49 (0.88) | 1.48 (0.18,2.79) | **2.79 [1.50, 5.19]** | **0.001**** |

^a^ adjusted for age, sex, Townsend deprivation index, ethnicity, education, body mass index, alcohol drinking, smoking status, physical activity, history of hypertension, diabetes, hyperlipidemia at index date, and adherence to cardioprotective diet.

Note that some lower or upper 95% CIs are presented as 1.00 due to rounding.

*CI, confidence interval; CVD, cardiovascular disease; HR, hazard ratio; IR, incident rate.*

# Table S13. Results of step function for the analysis with unmet proportional hazard assumptions over different time intervals (0-5, 5-10, >10 years follow-up)

|  | **CD-any CVD** | | **IBD-U-any CVD** | |
| --- | --- | --- | --- | --- |
| **Time interval** | **HR (95% CI)** | ***P*** | **HR (95% CI)** | ***P*** |
| 0-5 years | 2.15 [1.17, 3.94] | 0.013 | 4.56 [2.41, 8.62] | <0.001 |
| 5-10 years | 1.52 [1.02, 2.27] | 0.04 | 1.71 [1.01, 2.90] | 0.048 |
| >10 years | 1.43 [1.07, 1.91] | 0.015 | 1.24 [0.84, 1.83] | 0.285 |
|  | **IBD-heart failure** | | **IBD-U-heart failure** | |
| **Time interval** | **HR (95% CI)** | ***P*** | **HR (95% CI)** | ***P*** |
| 0-5 years | 2.51 [1.20, 5.23] | 0.014 | 1.80 [0.21, 15.47] | 0.593 |
| 5-10 years | 2.15 [1.44, 3.23] | <0.001 | 4.23 [1.90, 9.43] | <0.001 |
| >10 years | 1.32 [0.97, 1.78] | 0.075 | 0.99 [0.35, 2.80] | 0.992 |
|  | **CD-Peripheral artery disease** | |  |  |
| **Time interval** | **HR (95% CI)** | ***P*** |  |  |
| 0-5 years | / | / |  |  |
| 5-10 years | 2.02 [0.54, 7.52] | 0.293 |  |  |
| >10 years | / | / |  |  |

Model were all adjusted for age, sex, Townsend deprivation index, ethnicity, education, body mass index, alcohol drinking, smoking status, physical activity, history of hypertension, diabetes, hyperlipidemia at index date, and adherence to cardioprotective diet.

*CI, confidence interval; CVD, cardiovascular disease; HR, hazard ratio.*

#

# Table S14. Associations of inflammatory bowel disease (IBD), Crohn’s disease (CD), ulcerative colitis (UC), and IBD-unclassified (IBD-U) with inflammatory biomarkers.

| **Exposure: IBD** | | | | | **Exposure: CD** | | | | |
| --- | --- | --- | --- | --- | --- | --- | --- | --- | --- |
| **Inflammatory biomarkers** | **Beta** | **SE** | ***P*** | ***P* adjust ^a^** | **Inflammatory biomarkers** | **Beta** | **SE** | ***P*** | ***P* adjust ^a^** |
| Albumin | -0.245 | 0.026 | 1.49E-20 | 2.24E-19 | Albumin | -0.326 | 0.056 | 8.55E-09 | 1.28E-07 |
| CRP | 0.280 | 0.026 | 3.82E-26 | 5.73E-25 | CRP | 0.420 | 0.056 | 1.06E-13 | 1.59E-12 |
| Lymphocyte | -0.117 | 0.027 | 1.46E-05 | 2.19E-04 | Lymphocyte | -0.206 | 0.056 | 2.44E-04 | 0.004 |
| Platelet | 0.213 | 0.026 | 6.22E-16 | 9.33E-15 | Platelet | 0.264 | 0.056 | 2.93E-06 | 4.40E-05 |
| Eosinophil | 0.094 | 0.027 | 4.86E-04 | 0.007 | Eosinophil | 0.043 | 0.058 | 0.461 | 1.000 |
| Monocyte | 0.011 | 0.026 | 0.679 | 1.000 | Monocyte | -0.061 | 0.056 | 0.276 | 1.000 |
| Neutrophil | 0.251 | 0.026 | 1.20E-21 | 1.80E-20 | Neutrophil | 0.270 | 0.055 | 1.15E-06 | 1.72E-05 |
| WBC | 0.118 | 0.026 | 6.61E-06 | 9.92E-05 | WBC | 0.131 | 0.054 | 0.016 | 0.240 |
| LMR | -0.068 | 0.027 | 0.011 | 0.171 | LMR | -0.020 | 0.058 | 0.728 | 1.000 |
| NLR | 0.434 | 0.027 | 1.47E-58 | 2.21E-57 | NLR | 0.521 | 0.057 | 8.24E-20 | 1.24E-18 |
| PLR | 0.383 | 0.027 | 1.86E-46 | 2.79E-45 | PLR | 0.459 | 0.057 | 5.92E-16 | 8.88E-15 |
| CAR | 0.288 | 0.026 | 1.72E-27 | 2.58E-26 | CAR | 0.431 | 0.056 | 2.19E-14 | 3.28E-13 |
| SII | 0.468 | 0.027 | 1.26E-67 | 1.89E-66 | SII | 0.542 | 0.057 | 3.56E-21 | 5.34E-20 |
| SIRI | 0.277 | 0.027 | 2.32E-25 | 3.48E-24 | SIRI | 0.376 | 0.056 | 2.19E-11 | 3.28E-10 |
| INFLA score | 0.353 | 0.026 | 6.34E-43 | 9.51E-42 | INFLA score | 0.469 | 0.054 | 6.17E-18 | 9.26E-17 |
| **Exposure: UC** | | | | | **Exposure: IBD-U** | | | | |
| **Inflammatory biomarkers** | **Beta** | **SE** | ***P*** | ***P* adjust ^a^** | **Inflammatory biomarkers** | **Beta** | **SE** | ***P*** | ***P* adjust ^a^** |
| Albumin | -0.215 | 0.033 | 6.10E-11 | 9.15E-10 | Albumin | -0.257 | 0.073 | 4.08E-04 | 0.006 |
| CRP | 0.226 | 0.033 | 6.15E-12 | 9.22E-11 | CRP | 0.276 | 0.073 | 1.79E-04 | 0.003 |
| Lymphocyte | -0.105 | 0.034 | 0.002 | 0.028 | Lymphocyte | -0.132 | 0.072 | 0.069 | 1.000 |
| Platelet | 0.226 | 0.033 | 5.23E-12 | 7.84E-11 | Platelet | 0.031 | 0.073 | 0.667 | 1.000 |
| Eosinophil | 0.112 | 0.034 | 0.001 | 0.014 | Eosinophil | 0.082 | 0.074 | 0.271 | 1.000 |
| Monocyte | 0.043 | 0.033 | 0.194 | 1.000 | Monocyte | -0.031 | 0.073 | 0.671 | 1.000 |
| Neutrophil | 0.253 | 0.033 | 1.06E-14 | 1.59E-13 | Neutrophil | 0.205 | 0.073 | 0.005 | 0.074 |
| WBC | 0.114 | 0.033 | 0.001 | 0.008 | WBC | 0.119 | 0.072 | 0.099 | 1.000 |
| LMR | -0.084 | 0.033 | 0.011 | 0.171 | LMR | -0.117 | 0.072 | 0.101 | 1.000 |
| NLR | 0.404 | 0.033 | 2.36E-33 | 3.54E-32 | NLR | 0.452 | 0.073 | 8.38E-10 | 1.26E-08 |
| PLR | 0.362 | 0.033 | 1.65E-27 | 2.47E-26 | PLR | 0.342 | 0.074 | 4.24E-06 | 6.36E-05 |
| CAR | 0.231 | 0.033 | 1.97E-12 | 2.96E-11 | CAR | 0.287 | 0.073 | 9.86E-05 | 0.001 |
| SII | 0.448 | 0.033 | 1.68E-40 | 2.52E-39 | SII | 0.458 | 0.074 | 5.72E-10 | 8.58E-09 |
| SIRI | 0.256 | 0.033 | 1.37E-14 | 2.05E-13 | SIRI | 0.250 | 0.072 | 0.001 | 0.008 |
| INFLA score | 0.336 | 0.032 | 9.69E-26 | 1.45E-24 | INFLA score | 0.223 | 0.071 | 0.002 | 0.026 |

^a^ All *P* values were two-sided, and Bonferroni correction was used by IBD subtypes for multiple comparisons.

*CRP, C-reactive protein; LMR, lymphocyte to monocyte ratio; NLR, neutrophil to lymphocyte ratio; PLR, Platelet to lymphocyte ratio; CAR, C-reactive protein to albumin ratio; SII, Systemic immune-inflammatory index; SIRI, Systemic inflammation response index.*

# Table S15. Associations of inflammatory biomarkers with nine cardiovascular disease (CVD) outcomes.

| **Outcome: Any CVD** | | | | **Outcome: Ischemic heart disease** | | | |
| --- | --- | --- | --- | --- | --- | --- | --- |
| **Inflammatory biomarkers** | **HR (95%CI)** | ***P*** | ***P* adjust ^a^** | **Inflammatory biomarkers** | **HR (95%CI)** | ***P*** | ***P* adjust ^a^** |
| Albumin | 0.91 [0.88, 0.94] | 6.73E-08 | 1.01E-06 | Albumin | 0.95 [0.90, 1.00] | 0.035 | 0.531 |
| CRP | 1.08 [1.05, 1.11] | 2.31E-08 | 3.47E-07 | CRP | 1.07 [1.02, 1.11] | 0.002 | 0.029 |
| Lymphocyte | 1.01 [0.98, 1.04] | 0.422 | 1.000 | Lymphocyte | 1.03 [1.00, 1.06] | 0.059 | 0.881 |
| Platelet | 1.00 [0.97, 1.04] | 0.789 | 1.000 | Platelet | 1.03 [0.98, 1.09] | 0.246 | 1.000 |
| Eosinophil | 1.01 [0.98, 1.05] | 0.417 | 1.000 | Eosinophil | 1.02 [0.97, 1.07] | 0.421 | 1.000 |
| Monocyte | 1.04 [1.02, 1.07] | 0.003 | 0.038 | Monocyte | 1.05 [1.01, 1.09] | 0.008 | 0.125 |
| Neutrophil | 1.08 [1.05, 1.12] | 5.35E-06 | 8.03E-05 | Neutrophil | 1.08 [1.03, 1.14] | 0.002 | 0.033 |
| WBC | 1.05 [1.03, 1.08] | 1.40E-05 | 2.10E-04 | WBC | 1.07 [1.03, 1.10] | 9.69E-05 | 0.001 |
| LMR | 0.99 [0.95, 1.03] | 0.626 | 1.000 | LMR | 1.01 [0.97, 1.06] | 0.598 | 1.000 |
| NLR | 1.05 [1.02, 1.07] | 2.93E-04 | 0.004 | NLR | 1.04 [1.00, 1.09] | 0.037 | 0.548 |
| PLR | 1.02 [0.99, 1.05] | 0.118 | 1.000 | PLR | 1.02 [0.97, 1.06] | 0.504 | 1.000 |
| CAR | 1.08 [1.05, 1.11] | 9.47E-09 | 1.42E-07 | CAR | 1.07 [1.02, 1.11] | 0.002 | 0.028 |
| SII | 1.05 [1.02, 1.08] | 1.97E-04 | 0.003 | SII | 1.05 [1.01, 1.09] | 0.009 | 0.139 |
| SIRI | 1.04 [1.02, 1.06] | 4.74E-05 | 0.001 | SIRI | 1.03 [0.99, 1.06] | 0.102 | 1.000 |
| INFLA score | 1.10 [1.06, 1.14] | 7.43E-07 | 1.12E-05 | INFLA score | 1.11 [1.05, 1.17] | 2.53E-04 | 0.004 |
| **Outcome: Cerebrovascular disease** | | | | **Outcome: Heart failure** | | | |
| **Inflammatory biomarkers** | **HR (95%CI)** | ***P*** | ***P* adjust ^a^** | **Inflammatory biomarkers** | **HR (95%CI)** | ***P*** | ***P* adjust ^a^** |
| Albumin | 0.91 [0.84, 0.99] | 0.022 | 0.323 | Albumin | 0.83 [0.77, 0.90] | 1.20E-05 | 1.80E-04 |
| CRP | 1.09 [1.03, 1.15] | 0.002 | 0.027 | CRP | 1.14 [1.08, 1.20] | 4.01E-07 | 6.02E-06 |
| Lymphocyte | 1.02 [0.96, 1.08] | 0.611 | 1.000 | Lymphocyte | 0.96 [0.86, 1.08] | 0.487 | 1.000 |
| Platelet | 1.02 [0.94, 1.11] | 0.616 | 1.000 | Platelet | 1.04 [0.95, 1.13] | 0.395 | 1.000 |
| Eosinophil | 1.05 [0.98, 1.13] | 0.186 | 1.000 | Eosinophil | 1.06 [0.99, 1.15] | 0.100 | 1.000 |
| Monocyte | 1.03 [0.97, 1.10] | 0.329 | 1.000 | Monocyte | 1.09 [1.04, 1.15] | 0.001 | 0.013 |
| Neutrophil | 1.05 [0.97, 1.14] | 0.196 | 1.000 | Neutrophil | 1.11 [1.03, 1.20] | 0.009 | 0.130 |
| WBC | 1.05 [0.99, 1.11] | 0.129 | 1.000 | WBC | 1.06 [1.00, 1.12] | 0.037 | 0.553 |
| LMR | 0.96 [0.86, 1.08] | 0.493 | 1.000 | LMR | 0.78 [0.65, 0.93] | 0.005 | 0.082 |
| NLR | 1.03 [0.96, 1.09] | 0.429 | 1.000 | NLR | 1.09 [1.05, 1.14] | 4.41E-05 | 0.001 |
| PLR | 1.03 [0.97, 1.09] | 0.283 | 1.000 | PLR | 1.06 [1.01, 1.11] | 0.020 | 0.305 |
| CAR | 1.09 [1.03, 1.15] | 0.002 | 0.031 | CAR | 1.14 [1.08, 1.19] | 1.77E-07 | 2.65E-06 |
| SII | 1.04 [0.98, 1.10] | 0.233 | 1.000 | SII | 1.10 [1.05, 1.14] | 1.06E-05 | 1.58E-04 |
| SIRI | 1.03 [0.99, 1.08] | 0.159 | 1.000 | SIRI | 1.07 [1.04, 1.10] | 2.48E-06 | 3.71E-05 |
| INFLA score | 1.06 [0.98, 1.15] | 0.159 | 1.000 | INFLA score | 1.17 [1.07, 1.28] | 3.77E-04 | 0.006 |
| **Outcome: Arrhythmias** | | | | **Outcome: Atrial fibrillation** | | | |
| **Inflammatory biomarkers** | **HR (95%CI)** | ***P*** | ***P* adjust ^a^** | **Inflammatory biomarkers** | **HR (95%CI)** | ***P*** | ***P* adjust ^a^** |
| Albumin | 0.92 [0.87, 0.97] | 0.004 | 0.055 | Albumin | 0.91 [0.85, 0.97] | 0.004 | 0.054 |
| CRP | 1.08 [1.04, 1.13] | 1.21E-04 | 0.002 | CRP | 1.08 [1.04, 1.14] | 0.001 | 0.008 |
| Lymphocyte | 1.01 [0.96, 1.06] | 0.818 | 1.000 | Lymphocyte | 1.00 [0.94, 1.06] | 0.975 | 1.000 |
| Platelet | 0.94 [0.89, 1.00] | 0.046 | 0.688 | Platelet | 0.93 [0.87, 0.99] | 0.024 | 0.360 |
| Eosinophil | 0.99 [0.94, 1.05] | 0.804 | 1.000 | Eosinophil | 0.99 [0.93, 1.05] | 0.684 | 1.000 |
| Monocyte | 1.06 [1.02, 1.10] | 0.003 | 0.043 | Monocyte | 1.06 [1.01, 1.11] | 0.017 | 0.256 |
| Neutrophil | 1.07 [1.02, 1.13] | 0.012 | 0.176 | Neutrophil | 1.06 [1.00, 1.13] | 0.067 | 1.000 |
| WBC | 1.05 [1.01, 1.09] | 0.022 | 0.334 | WBC | 1.04 [0.99, 1.09] | 0.119 | 1.000 |
| LMR | 0.90 [0.81, 1.00] | 0.047 | 0.709 | LMR | 0.91 [0.81, 1.02] | 0.100 | 1.000 |
| NLR | 1.06 [1.03, 1.10] | 0.001 | 0.017 | NLR | 1.07 [1.03, 1.12] | 0.001 | 0.017 |
| PLR | 1.02 [0.97, 1.07] | 0.505 | 1.000 | PLR | 1.03 [0.98, 1.08] | 0.252 | 1.000 |
| CAR | 1.09 [1.04, 1.13] | 6.58E-05 | 0.001 | CAR | 1.08 [1.04, 1.13] | 3.84E-04 | 0.006 |
| SII | 1.05 [1.00, 1.09] | 0.032 | 0.483 | SII | 1.05 [1.01, 1.10] | 0.026 | 0.390 |
| SIRI | 1.05 [1.02, 1.07] | 9.30E-05 | 0.001 | SIRI | 1.05 [1.02, 1.08] | 1.95E-04 | 0.003 |
| INFLA score | 1.06 [1.00, 1.12] | 0.051 | 0.765 | INFLA score | 1.06 [0.99, 1.13] | 0.075 | 1.000 |
| **Outcome: Other supraventricular arrhythmias** | | | | **Outcome: Venous thromboembolism** | | | |
| **Inflammatory biomarkers** | **HR (95%CI)** | ***P*** | ***P* adjust ^a^** | **Inflammatory biomarkers** | **HR (95%CI)** | ***P*** | ***P* adjust ^a^** |
| Albumin | 0.92 [0.77, 1.10] | 0.369 | 1.000 | Albumin | 0.81 [0.74, 0.88] | 1.82E-06 | 2.73E-05 |
| CRP | 1.01 [0.86, 1.20] | 0.873 | 1.000 | CRP | 1.10 [1.03, 1.17] | 0.003 | 0.038 |
| Lymphocyte | 0.79 [0.56, 1.10] | 0.166 | 1.000 | Lymphocyte | 0.95 [0.83, 1.08] | 0.453 | 1.000 |
| Platelet | 0.93 [0.77, 1.13] | 0.481 | 1.000 | Platelet | 1.05 [0.97, 1.15] | 0.227 | 1.000 |
| Eosinophil | 1.02 [0.85, 1.21] | 0.866 | 1.000 | Eosinophil | 0.99 [0.91, 1.08] | 0.847 | 1.000 |
| Monocyte | 1.09 [0.97, 1.22] | 0.146 | 1.000 | Monocyte | 0.99 [0.90, 1.08] | 0.796 | 1.000 |
| Neutrophil | 1.10 [0.93, 1.31] | 0.278 | 1.000 | Neutrophil | 1.08 [0.99, 1.17] | 0.081 | 1.000 |
| WBC | 1.03 [0.87, 1.21] | 0.741 | 1.000 | WBC | 1.03 [0.96, 1.11] | 0.404 | 1.000 |
| LMR | 0.74 [0.50, 1.10] | 0.140 | 1.000 | LMR | 1.03 [0.99, 1.08] | 0.089 | 1.000 |
| NLR | 1.11 [1.01, 1.22] | 0.026 | 0.386 | NLR | 1.05 [0.99, 1.11] | 0.111 | 1.000 |
| PLR | 1.06 [0.96, 1.19] | 0.254 | 1.000 | PLR | 1.04 [0.99, 1.10] | 0.098 | 1.000 |
| CAR | 1.03 [0.87, 1.21] | 0.762 | 1.000 | CAR | 1.10 [1.04, 1.17] | 0.002 | 0.025 |
| SII | 1.11 [1.00, 1.22] | 0.048 | 0.721 | SII | 1.06 [1.00, 1.12] | 0.065 | 0.971 |
| SIRI | 1.07 [1.01, 1.14] | 0.025 | 0.375 | SIRI | 1.04 [1.00, 1.09] | 0.045 | 0.676 |
| INFLA score | 1.05 [0.87, 1.27] | 0.601 | 1.000 | INFLA score | 1.16 [1.06, 1.26] | 0.001 | 0.022 |
| **Outcome: Deep vein thrombosis** | | | |  |  |  |  |
| **Inflammatory biomarkers** | **HR (95%CI)** | ***P*** | ***P* adjust ^a^** |  |  |  |  |
| Albumin | 0.80 [0.71, 0.89] | 7.98E-05 | 0.001 |  |  |  |  |
| CRP | 1.08 [0.99, 1.18] | 0.065 | 0.982 |  |  |  |  |
| Lymphocyte | 1.01 [0.91, 1.12] | 0.872 | 1.000 |  |  |  |  |
| Platelet | 1.04 [0.93, 1.17] | 0.499 | 1.000 |  |  |  |  |
| Eosinophil | 1.06 [0.96, 1.17] | 0.262 | 1.000 |  |  |  |  |
| Monocyte | 0.99 [0.88, 1.11] | 0.899 | 1.000 |  |  |  |  |
| Neutrophil | 1.07 [0.96, 1.20] | 0.204 | 1.000 |  |  |  |  |
| WBC | 1.05 [0.96, 1.14] | 0.286 | 1.000 |  |  |  |  |
| LMR | 1.04 [1.00, 1.09] | 0.041 | 0.613 |  |  |  |  |
| NLR | 1.02 [0.93, 1.12] | 0.678 | 1.000 |  |  |  |  |
| PLR | 1.02 [0.93, 1.12] | 0.667 | 1.000 |  |  |  |  |
| CAR | 1.09 [1.00, 1.18] | 0.049 | 0.730 |  |  |  |  |
| SII | 1.04 [0.95, 1.14] | 0.374 | 1.000 |  |  |  |  |
| SIRI | 1.04 [0.99, 1.10] | 0.131 | 1.000 |  |  |  |  |
| INFLA score | 1.13 [1.00, 1.27] | 0.043 | 0.644 |  |  |  |  |

^a^ All *P* values were two-sided, and Bonferroni correction was used by CVD subtypes for multiple comparisons.

Note that some lower or upper 95% CIs are presented as 1.00 due to rounding.

*CVD, cardiovascular diseases; CRP, C-reactive protein; LMR, lymphocyte to monocyte ratio; NLR, neutrophil to lymphocyte ratio; PLR, Platelet to lymphocyte ratio; CAR, C-reactive protein to albumin ratio; SII, Systemic immune-inflammatory index; SIRI, Systemic inflammation response index.*

# Table S16. Results of mediation analysis.

| **CVD Outcomes: Any CVD** | | | | | |
| --- | --- | --- | --- | --- | --- |
| **Exposure** | **Inflammation biomarker** | **Proportion mediation** | **95% Lower** | **95% Upper** | ***P*** |
| IBD | Albumin | 7.53% | 3.96% | 13.58% | <0.001 |
|  | CRP | 6.78% | 3.60% | 11.79% | <0.001 |
|  | Lymphocyte | -0.53% | -2.13% | 0.57% | 0.298 |
|  | Platelet | 0.05% | -2.85% | 2.87% | 0.964 |
|  | Eosinophil | 0.38% | -0.59% | 1.75% | 0.438 |
|  | Monocyte | 0.17% | -0.78% | 1.21% | 0.628 |
|  | Neutrophil | 6.01% | 3.05% | 11.48% | <0.001 |
|  | WBC | 2.02% | 0.83% | 3.93% | <0.001 |
|  | LMR | 0.17% | -0.97% | 1.49% | 0.708 |
|  | NLR | 5.70% | 1.47% | 12.06% | 0.010 |
|  | PLR | 2.14% | -1.85% | 6.66% | 0.304 |
|  | CAR | 7.04% | 3.91% | 12.06% | <0.001 |
|  | SII | 6.55% | 2.31% | 12.78% | 0.010 |
|  | SIRI | 3.52% | 1.57% | 6.64% | <0.001 |
|  | INFLA score | 10.17% | 4.90% | 18.54% | <0.001 |
| CD | Albumin | 3.48% | -2.59% | 13.20% | 0.240 |
|  | CRP | 11.03% | 4.72% | 29.48% | <0.001 |
|  | Lymphocyte | -3.27% | -10.77% | -0.11% | 0.050 |
|  | Platelet | -0.13% | -5.87% | 6.10% | 0.964 |
|  | Eosinophil | 0.24% | -1.29% | 2.79% | 0.592 |
|  | Monocyte | -0.58% | -3.16% | 0.51% | 0.294 |
|  | Neutrophil | 4.26% | 0.18% | 12.04% | 0.040 |
|  | WBC | 2.70% | 0.09% | 8.66% | 0.042 |
|  | LMR | -0.01% | -1.39% | 1.29% | 0.956 |
|  | NLR | 1.74% | -8.54% | 11.94% | 0.692 |
|  | PLR | -3.23% | -15.08% | 6.84% | 0.496 |
|  | CAR | 11.32% | 4.50% | 26.75% | <0.001 |
|  | SII | 2.67% | -7.16% | 15.63% | 0.562 |
|  | SIRI | 5.01% | -0.33% | 15.84% | 0.066 |
|  | INFLA score | 14.53% | 5.51% | 38.31% | 0.004 |
| UC | Albumin | 12.41% | 5.39% | 41.41% | 0.008 |
|  | CRP | 8.14% | 2.99% | 28.69% | 0.010 |
|  | Lymphocyte | -0.59% | -4.81% | 1.68% | 0.512 |
|  | Platelet | -0.93% | -10.90% | 6.59% | 0.696 |
|  | Eosinophil | -0.25% | -4.97% | 2.92% | 0.810 |
|  | Monocyte | 0.67% | -0.67% | 3.93% | 0.240 |
|  | Neutrophil | 8.90% | 2.43% | 37.15% | 0.010 |
|  | WBC | 2.75% | 0.62% | 10.26% | 0.006 |
|  | LMR | 0.07% | -3.38% | 3.86% | 0.918 |
|  | NLR | 10.13% | 3.37% | 38.24% | 0.004 |
|  | PLR | 3.93% | -2.91% | 18.15% | 0.216 |
|  | CAR | 8.37% | 3.35% | 27.93% | 0.006 |
|  | SII | 10.66% | 3.17% | 46.26% | 0.012 |
|  | SIRI | 4.53% | 0.93% | 16.82% | 0.016 |
|  | INFLA score | 10.45% | 2.03% | 39.16% | 0.018 |
| IBD-U | Albumin | 2.89% | -2.15% | 12.49% | 0.258 |
|  | CRP | 1.26% | -3.87% | 7.60% | 0.574 |
|  | Lymphocyte | 0.48% | -3.08% | 5.15% | 0.610 |
|  | Platelet | 0.01% | -2.17% | 2.35% | 0.952 |
|  | Eosinophil | 0.56% | -1.58% | 4.66% | 0.462 |
|  | Monocyte | -0.45% | -5.98% | 2.96% | 0.626 |
|  | Neutrophil | 4.01% | 0.10% | 13.75% | 0.042 |
|  | WBC | 1.95% | -0.40% | 8.28% | 0.134 |
|  | LMR | 1.21% | -1.98% | 7.40% | 0.360 |
|  | NLR | 0.72% | -6.02% | 9.99% | 0.830 |
|  | PLR | -0.02% | -6.39% | 6.82% | 0.994 |
|  | CAR | 1.47% | -3.22% | 7.72% | 0.488 |
|  | SII | 1.79% | -5.49% | 12.69% | 0.574 |
|  | SIRI | 2.83% | -1.80% | 11.32% | 0.176 |
|  | INFLA score | 5.27% | 0.75% | 17.32% | 0.016 |
| CVD Outcomes: Ischemic heart disease | | | | | |
| **Exposure** | **Inflammation biomarker** | **Proportion mediation** | **95% Lower** | **95% Upper** | ***P*** |
| IBD | Albumin | 6.75% | -2.13% | 38.29% | 0.096 |
|  | CRP | 9.89% | 1.43% | 58.68% | 0.034 |
|  | Lymphocyte | -1.74% | -11.09% | 0.58% | 0.090 |
|  | Platelet | 3.36% | -4.46% | 24.23% | 0.300 |
|  | Eosinophil | 0.92% | -2.31% | 7.14% | 0.422 |
|  | Monocyte | 0.30% | -2.48% | 4.54% | 0.650 |
|  | Neutrophil | 9.84% | 1.76% | 61.81% | 0.032 |
|  | WBC | 3.83% | 0.69% | 19.06% | 0.048 |
|  | LMR | -0.41% | -6.57% | 2.44% | 0.614 |
|  | NLR | 8.96% | -3.11% | 55.18% | 0.116 |
|  | PLR | 2.76% | -12.89% | 28.66% | 0.618 |
|  | CAR | 9.83% | 1.93% | 51.46% | 0.026 |
|  | SII | 12.40% | -0.96% | 70.12% | 0.062 |
|  | SIRI | 3.68% | -1.94% | 23.90% | 0.172 |
|  | INFLA score | 19.40% | 4.69% | 115.70% | 0.028 |
| IBD-U | Albumin | 4.03% | -2.39% | 17.39% | 0.200 |
|  | CRP | 1.65% | -4.20% | 10.36% | 0.520 |
|  | Lymphocyte | 0.06% | -5.10% | 4.95% | 0.914 |
|  | Platelet | 0.15% | -3.94% | 4.61% | 0.812 |
|  | Eosinophil | 0.91% | -1.62% | 7.50% | 0.390 |
|  | Monocyte | -0.71% | -8.53% | 3.99% | 0.620 |
|  | Neutrophil | 3.62% | -1.00% | 15.75% | 0.122 |
|  | WBC | 1.97% | -0.81% | 10.51% | 0.188 |
|  | LMR | 1.16% | -2.79% | 9.16% | 0.438 |
|  | NLR | 0.32% | -9.14% | 12.07% | 0.930 |
|  | PLR | -0.19% | -8.81% | 8.79% | 0.952 |
|  | CAR | 1.77% | -3.71% | 10.14% | 0.470 |
|  | SII | 2.85% | -5.87% | 18.73% | 0.456 |
|  | SIRI | 1.52% | -5.47% | 10.99% | 0.528 |
|  | INFLA score | 5.31% | 0.17% | 21.66% | 0.044 |
| **CVD Outcomes: Cerebrovascular disease** | | | | | |
| **Exposure** | **Inflammation biomarker** | **Proportion mediation** | **95% Lower** | **95% Upper** | ***P*** |
| CD | Albumin | 2.25% | -20.03% | 31.64% | 0.714 |
|  | CRP | 11.22% | -68.47% | 88.74% | 0.118 |
|  | Lymphocyte | 0.24% | -20.23% | 17.33% | 0.934 |
|  | Platelet | -0.63% | -29.17% | 28.02% | 0.878 |
|  | Eosinophil | 0.10% | -4.13% | 5.47% | 0.818 |
|  | Monocyte | -0.34% | -6.50% | 2.31% | 0.616 |
|  | Neutrophil | -2.27% | -35.88% | 16.31% | 0.632 |
|  | WBC | -0.49% | -12.22% | 6.60% | 0.774 |
|  | LMR | 0.51% | -13.45% | 35.48% | 0.818 |
|  | NLR | 6.34% | -39.12% | 49.32% | 0.466 |
|  | PLR | 10.76% | -8.15% | 79.06% | 0.138 |
|  | CAR | 11.95% | -25.39% | 91.47% | 0.086 |
|  | SII | 10.38% | -12.11% | 89.43% | 0.222 |
|  | SIRI | 7.77% | -44.65% | 70.54% | 0.130 |
|  | INFLA score | -4.29% | -51.91% | 36.72% | 0.658 |
| **CVD Outcomes: Heart failure** | | | | | |
| **Exposure** | **Inflammation biomarker** | **Proportion mediation** | **95% Lower** | **95% Upper** | ***P*** |
| IBD | Albumin | 8.91% | 3.93% | 19.22% | <0.001 |
|  | CRP | 7.41% | 3.48% | 15.63% | <0.001 |
|  | Lymphocyte | 0.73% | -2.61% | 4.20% | 0.610 |
|  | Platelet | 1.19% | -2.90% | 5.92% | 0.506 |
|  | Eosinophil | 1.08% | -0.20% | 3.50% | 0.092 |
|  | Monocyte | 0.23% | -0.97% | 1.57% | 0.628 |
|  | Neutrophil | 4.45% | 0.37% | 11.99% | 0.036 |
|  | WBC | 1.34% | 0.00% | 3.73% | 0.050 |
|  | LMR | 3.32% | 0.44% | 10.37% | 0.022 |
|  | NLR | 7.22% | 2.40% | 17.11% | 0.002 |
|  | PLR | 3.99% | -0.15% | 10.14% | 0.060 |
|  | CAR | 7.61% | 3.80% | 15.48% | <0.001 |
|  | SII | 8.54% | 3.59% | 19.53% | 0.004 |
|  | SIRI | 3.76% | 1.86% | 8.14% | <0.001 |
|  | INFLA score | 10.49% | 3.18% | 24.75% | 0.004 |
| CD | Albumin | 7.60% | -1.30% | 30.37% | 0.090 |
|  | CRP | 11.75% | 4.00% | 52.38% | 0.016 |
|  | Lymphocyte | -3.76% | -16.49% | -0.04% | 0.048 |
|  | Platelet | -3.22% | -17.76% | 4.97% | 0.358 |
|  | Eosinophil | 0.04% | -2.11% | 2.90% | 0.882 |
|  | Monocyte | -0.51% | -4.09% | 0.64% | 0.378 |
|  | Neutrophil | 2.14% | -5.16% | 13.09% | 0.532 |
|  | WBC | 2.01% | -1.15% | 9.41% | 0.186 |
|  | LMR | 0.01% | -2.95% | 3.70% | 0.974 |
|  | NLR | 7.16% | -5.16% | 32.25% | 0.216 |
|  | PLR | -3.04% | -24.17% | 14.17% | 0.686 |
|  | CAR | 12.36% | 3.91% | 42.11% | 0.006 |
|  | SII | 3.03% | -11.68% | 23.92% | 0.620 |
|  | SIRI | 6.96% | 0.52% | 32.98% | 0.034 |
|  | INFLA score | 9.44% | -3.31% | 45.50% | 0.152 |
| UC | Albumin | 9.97% | 0.94% | 55.71% | 0.038 |
|  | CRP | 9.05% | -15.46% | 51.93% | 0.056 |
|  | Lymphocyte | 2.87% | -6.90% | 22.71% | 0.372 |
|  | Platelet | 6.42% | -1.63% | 34.89% | 0.092 |
|  | Eosinophil | 2.64% | -3.37% | 17.36% | 0.126 |
|  | Monocyte | 1.16% | -1.88% | 7.95% | 0.218 |
|  | Neutrophil | 8.05% | -7.21% | 70.62% | 0.098 |
|  | WBC | 1.91% | -1.67% | 14.51% | 0.168 |
|  | LMR | 8.01% | 0.15% | 40.20% | 0.046 |
|  | NLR | 10.73% | 1.07% | 68.51% | 0.050 |
|  | PLR | 6.08% | -3.27% | 38.13% | 0.078 |
|  | CAR | 9.22% | 2.51% | 63.43% | 0.038 |
|  | SII | 14.51% | -56.70% | 126.45% | 0.078 |
|  | SIRI | 4.77% | -5.58% | 26.59% | 0.054 |
|  | INFLA score | 16.05% | -4.95% | 95.54% | 0.054 |
| **CVD Outcomes: Arrhythmias** | | | | | |
| **Exposure** | **Inflammation biomarker** | **Proportion mediation** | **95% Lower** | **95% Upper** | ***P*** |
| IBD | Albumin | 6.24% | 1.22% | 16.87% | 0.014 |
|  | CRP | 7.36% | 2.61% | 19.14% | <0.001 |
|  | Lymphocyte | -0.29% | -3.17% | 1.82% | 0.766 |
|  | Platelet | -4.51% | -13.92% | -0.50% | 0.034 |
|  | Eosinophil | -0.19% | -2.40% | 1.82% | 0.798 |
|  | Monocyte | 0.23% | -1.13% | 1.83% | 0.628 |
|  | Neutrophil | 4.95% | 0.32% | 15.00% | 0.038 |
|  | WBC | 1.72% | 0.13% | 5.47% | 0.030 |
|  | LMR | 2.20% | -0.19% | 8.55% | 0.070 |
|  | NLR | 8.14% | 1.61% | 22.96% | 0.012 |
|  | PLR | 1.27% | -6.77% | 9.53% | 0.728 |
|  | CAR | 7.62% | 3.02% | 18.50% | <0.001 |
|  | SII | 6.00% | -1.27% | 18.87% | 0.086 |
|  | SIRI | 4.35% | 1.83% | 11.11% | <0.001 |
|  | INFLA score | 5.73% | -1.60% | 19.79% | 0.126 |
| CD | Albumin | 0.11% | -9.54% | 9.35% | 0.982 |
|  | CRP | 6.15% | -0.79% | 24.47% | 0.090 |
|  | Lymphocyte | -3.03% | -11.92% | 0.27% | 0.086 |
|  | Platelet | -8.79% | -26.36% | -2.24% | 0.004 |
|  | Eosinophil | 0.04% | -1.61% | 2.31% | 0.844 |
|  | Monocyte | -0.35% | -3.27% | 0.81% | 0.502 |
|  | Neutrophil | -0.67% | -8.23% | 5.81% | 0.798 |
|  | WBC | 0.66% | -2.30% | 4.96% | 0.554 |
|  | LMR | 0.01% | -1.88% | 2.45% | 0.960 |
|  | NLR | -3.02% | -21.20% | 9.07% | 0.572 |
|  | PLR | -14.76% | -46.42% | -0.84% | 0.038 |
|  | CAR | 6.34% | -1.13% | 19.74% | 0.084 |
|  | SII | -11.26% | -34.37% | 3.37% | 0.136 |
|  | SIRI | 1.32% | -7.58% | 11.85% | 0.720 |
|  | INFLA score | -2.14% | -15.40% | 11.29% | 0.730 |
| UC | Albumin | 9.44% | 2.74% | 38.85% | 0.012 |
|  | CRP | 7.26% | 2.11% | 29.34% | 0.018 |
|  | Lymphocyte | -0.08% | -4.52% | 3.86% | 0.954 |
|  | Platelet | -4.44% | -22.65% | 2.29% | 0.134 |
|  | Eosinophil | -0.35% | -6.17% | 3.41% | 0.764 |
|  | Monocyte | 1.00% | -0.82% | 5.36% | 0.198 |
|  | Neutrophil | 6.10% | -1.45% | 34.59% | 0.096 |
|  | WBC | 1.80% | -0.25% | 8.68% | 0.094 |
|  | LMR | 2.75% | -1.15% | 14.68% | 0.140 |
|  | NLR | 10.65% | 3.39% | 55.33% | 0.010 |
|  | PLR | 3.69% | -4.09% | 21.59% | 0.314 |
|  | CAR | 7.41% | 2.46% | 29.01% | 0.012 |
|  | SII | 9.00% | 0.81% | 43.67% | 0.046 |
|  | SIRI | 4.76% | 1.48% | 19.33% | 0.022 |
|  | INFLA score | 4.07% | -6.20% | 24.66% | 0.360 |
| **CVD Outcomes: Atrial fibrillation** | | | | | |
| **Exposure** | **Inflammation biomarker** | **Proportion mediation** | **95% Lower** | **95% Upper** | ***P*** |
| IBD | Albumin | 7.11% | 1.33% | 20.40% | 0.018 |
|  | CRP | 7.47% | 2.30% | 23.12% | <0.001 |
|  | Lymphocyte | -0.11% | -3.64% | 2.72% | 0.932 |
|  | Platelet | -5.70% | -19.75% | -1.04% | 0.018 |
|  | Eosinophil | -0.33% | -3.10% | 1.89% | 0.692 |
|  | Monocyte | 0.20% | -1.17% | 1.91% | 0.636 |
|  | Neutrophil | 3.81% | -1.55% | 14.71% | 0.146 |
|  | WBC | 1.36% | -0.56% | 5.61% | 0.144 |
|  | LMR | 1.97% | -0.57% | 8.80% | 0.144 |
|  | NLR | 9.12% | 1.81% | 29.25% | 0.012 |
|  | PLR | 3.05% | -4.54% | 14.60% | 0.410 |
|  | CAR | 7.59% | 2.66% | 22.02% | <0.001 |
|  | SII | 7.04% | -0.94% | 25.58% | 0.076 |
|  | SIRI | 4.53% | 1.76% | 13.13% | <0.001 |
|  | INFLA score | 5.95% | -2.37% | 24.43% | 0.152 |
| CD | Albumin | 2.37% | -7.32% | 14.36% | 0.570 |
|  | CRP | 5.19% | -2.57% | 24.84% | 0.180 |
|  | Lymphocyte | -2.37% | -11.89% | 1.50% | 0.186 |
|  | Platelet | -12.51% | -41.19% | -4.11% | 0.004 |
|  | Eosinophil | 0.07% | -1.63% | 2.67% | 0.820 |
|  | Monocyte | -0.34% | -3.57% | 0.91% | 0.532 |
|  | Neutrophil | -0.68% | -8.98% | 6.98% | 0.822 |
|  | WBC | 0.54% | -2.86% | 5.35% | 0.628 |
|  | LMR | 0.03% | -2.84% | 4.12% | 0.934 |
|  | NLR | -1.49% | -19.82% | 12.06% | 0.802 |
|  | PLR | -15.47% | -53.65% | 0.12% | 0.052 |
|  | CAR | 5.28% | -3.46% | 20.83% | 0.200 |
|  | SII | -12.62% | -41.60% | 3.56% | 0.126 |
|  | SIRI | 2.43% | -5.82% | 15.62% | 0.468 |
|  | INFLA score | -2.70% | -17.83% | 12.50% | 0.674 |
| UC | Albumin | 9.35% | 1.37% | 52.03% | 0.030 |
|  | CRP | 7.28% | 1.12% | 39.55% | 0.042 |
|  | Lymphocyte | 0.21% | -6.27% | 6.98% | 0.906 |
|  | Platelet | -4.76% | -33.69% | 4.25% | 0.160 |
|  | Eosinophil | -0.71% | -8.56% | 5.59% | 0.626 |
|  | Monocyte | 0.80% | -1.05% | 6.29% | 0.230 |
|  | Neutrophil | 5.43% | -4.60% | 49.17% | 0.194 |
|  | WBC | 1.53% | -1.61% | 10.96% | 0.252 |
|  | LMR | 1.86% | -3.25% | 13.99% | 0.312 |
|  | NLR | 12.79% | 3.67% | 101.97% | 0.036 |
|  | PLR | 6.10% | -1.99% | 36.79% | 0.110 |
|  | CAR | 7.25% | 1.55% | 40.96% | 0.026 |
|  | SII | 11.63% | -18.30% | 75.18% | 0.064 |
|  | SIRI | 5.17% | 1.22% | 27.86% | 0.038 |
|  | INFLA score | 5.27% | -8.95% | 38.57% | 0.292 |
| **CVD Outcomes: Other supraventricular arrhythmias** | | | | | |
| **Exposure** | **Inflammation biomarker** | **Proportion mediation** | **95% Lower** | **95% Upper** | ***P*** |
| IBD | Albumin | 3.22% | -7.07% | 19.21% | 0.388 |
|  | CRP | 0.08% | -13.38% | 13.67% | 0.988 |
|  | Lymphocyte | 4.22% | -5.01% | 23.48% | 0.272 |
|  | Platelet | -2.60% | -19.79% | 6.29% | 0.398 |
|  | Eosinophil | 0.22% | -3.87% | 4.71% | 0.856 |
|  | Monocyte | 0.10% | -1.18% | 2.02% | 0.702 |
|  | Neutrophil | 3.16% | -5.19% | 23.91% | 0.366 |
|  | WBC | 0.39% | -4.57% | 6.53% | 0.818 |
|  | LMR | 3.14% | -2.02% | 19.98% | 0.182 |
|  | NLR | 6.59% | -1.00% | 35.58% | 0.094 |
|  | PLR | 3.45% | -6.36% | 22.49% | 0.376 |
|  | CAR | 0.74% | -10.36% | 14.17% | 0.836 |
|  | SII | 6.93% | -3.53% | 34.45% | 0.118 |
|  | SIRI | 3.09% | 0.18% | 14.65% | 0.044 |
|  | INFLA score | 2.11% | -14.40% | 28.95% | 0.708 |
| **CVD Outcomes: Venous thromboembolism** | | | | | |
| **Exposure** | **Inflammation biomarker** | **Proportion mediation** | **95% Lower** | **95% Upper** | ***P*** |
| IBD | Albumin | 8.59% | 4.07% | 16.85% | <0.001 |
|  | CRP | 4.18% | 0.96% | 9.12% | 0.008 |
|  | Lymphocyte | 0.76% | -2.26% | 4.00% | 0.578 |
|  | Platelet | 1.59% | -1.93% | 5.70% | 0.350 |
|  | Eosinophil | -0.14% | -1.79% | 1.42% | 0.832 |
|  | Monocyte | -0.01% | -0.69% | 0.50% | 0.904 |
|  | Neutrophil | 2.49% | -1.49% | 7.74% | 0.190 |
|  | WBC | 0.50% | -1.17% | 2.56% | 0.502 |
|  | LMR | -0.41% | -1.40% | 0.05% | 0.078 |
|  | NLR | 2.47% | -2.66% | 9.06% | 0.316 |
|  | PLR | 2.51% | -1.56% | 7.50% | 0.240 |
|  | CAR | 4.39% | 1.26% | 9.18% | 0.008 |
|  | SII | 3.44% | -2.09% | 10.40% | 0.166 |
|  | SIRI | 1.86% | -0.21% | 4.82% | 0.082 |
|  | INFLA score | 8.03% | 1.95% | 17.97% | 0.014 |
| UC | Albumin | 7.49% | 2.79% | 18.14% | <0.001 |
|  | CRP | 3.16% | 0.07% | 9.56% | 0.046 |
|  | Lymphocyte | 4.17% | 0.20% | 12.64% | 0.040 |
|  | Platelet | 1.20% | -3.55% | 6.72% | 0.608 |
|  | Eosinophil | -1.46% | -5.86% | 0.66% | 0.166 |
|  | Monocyte | -0.28% | -2.36% | 0.76% | 0.496 |
|  | Neutrophil | 1.15% | -4.93% | 8.03% | 0.652 |
|  | WBC | -0.80% | -4.69% | 1.94% | 0.472 |
|  | LMR | -0.49% | -2.53% | 0.79% | 0.356 |
|  | NLR | 2.90% | -2.61% | 10.73% | 0.306 |
|  | PLR | 3.24% | -0.57% | 10.32% | 0.096 |
|  | CAR | 3.12% | -0.09% | 8.94% | 0.054 |
|  | SII | 3.47% | -2.29% | 12.65% | 0.236 |
|  | SIRI | 1.88% | -0.58% | 6.32% | 0.158 |
|  | INFLA score | 5.70% | -1.33% | 16.81% | 0.092 |
| **CVD Outcomes: Deep vein thrombosis** | | | | | |
| **Exposure** | **Inflammation biomarker** | **Proportion mediation** | **95% Lower** | **95% Upper** | ***P*** |
| IBD | Albumin | 5.95% | 2.24% | 11.56% | <0.001 |
|  | CRP | 2.00% | -1.10% | 5.65% | 0.186 |
|  | Lymphocyte | -0.22% | -1.84% | 1.18% | 0.750 |
|  | Platelet | 0.50% | -2.71% | 3.78% | 0.730 |
|  | Eosinophil | 0.54% | -0.51% | 2.13% | 0.308 |
|  | Monocyte | 0.00% | -0.59% | 0.45% | 0.950 |
|  | Neutrophil | 1.28% | -2.46% | 5.20% | 0.450 |
|  | WBC | 0.51% | -0.82% | 2.21% | 0.404 |
|  | LMR | -0.36% | -1.08% | -0.02% | 0.044 |
|  | NLR | -0.88% | -6.76% | 5.30% | 0.756 |
|  | PLR | -0.29% | -5.96% | 4.85% | 0.928 |
|  | CAR | 2.24% | -0.81% | 5.87% | 0.140 |
|  | SII | 0.51% | -5.42% | 6.64% | 0.860 |
|  | SIRI | 1.14% | -1.01% | 3.62% | 0.302 |
|  | INFLA score | 3.77% | -1.72% | 10.14% | 0.172 |
| UC | Albumin | 6.04% | 2.06% | 13.23% | <0.001 |
|  | CRP | 1.81% | -1.28% | 6.16% | 0.250 |
|  | Lymphocyte | 1.56% | -2.16% | 6.61% | 0.382 |
|  | Platelet | -0.11% | -4.72% | 4.22% | 0.956 |
|  | Eosinophil | 0.49% | -1.35% | 2.66% | 0.550 |
|  | Monocyte | 0.14% | -0.52% | 1.41% | 0.598 |
|  | Neutrophil | 2.21% | -2.51% | 8.32% | 0.326 |
|  | WBC | 0.36% | -1.48% | 2.65% | 0.654 |
|  | LMR | -0.17% | -2.09% | 1.57% | 0.780 |
|  | NLR | 1.68% | -3.53% | 7.11% | 0.520 |
|  | PLR | 1.50% | -2.90% | 6.54% | 0.484 |
|  | CAR | 1.87% | -1.29% | 5.87% | 0.220 |
|  | SII | 2.13% | -3.29% | 8.76% | 0.416 |
|  | SIRI | 1.78% | 0.03% | 4.51% | 0.044 |
|  | INFLA score | 4.37% | -1.99% | 12.71% | 0.134 |

Significance of mediating effects were determined by 1000 quasi-Bayesian approximation iterations.

CVD with less than 100 observed cases were not analyzed due to power issue, but still contributed to the analysis for corresponding main categories.

*CRP, C-reactive protein; LMR, lymphocyte to monocyte ratio; NLR, neutrophil to lymphocyte ratio; PLR, Platelet to lymphocyte ratio; CAR, C-reactive protein to albumin ratio; SII, Systemic immune-inflammatory index; SIRI, Systemic inflammation response index.*

# Table S17. Results of mediation analysis using stringent IBD subtype definition.

| **CVD Outcomes: Any CVD** | | | | | |
| --- | --- | --- | --- | --- | --- |
| **Exposure** | **Inflammation biomarker** | **Proportion mediation** | **95% Lower** | **95% Upper** | ***P*** |
| CD | Albumin | 4.46% | -1.73% | 15.62% | 0.160 |
|  | CRP | 10.76% | 4.48% | 28.54% | <0.001 |
|  | Lymphocyte | -2.96% | -10.77% | 0.08% | 0.064 |
|  | Platelet | -0.70% | -7.06% | 5.15% | 0.756 |
|  | Eosinophil | -0.07% | -2.51% | 1.72% | 0.864 |
|  | Monocyte | -0.34% | -2.99% | 0.66% | 0.464 |
|  | Neutrophil | 4.38% | -0.50% | 13.91% | 0.076 |
|  | WBC | 2.84% | 0.10% | 9.93% | 0.036 |
|  | LMR | -0.04% | -2.47% | 1.64% | 0.848 |
|  | NLR | 3.12% | -6.25% | 15.45% | 0.492 |
|  | PLR | -1.47% | -13.74% | 8.35% | 0.730 |
|  | CAR | 10.94% | 3.99% | 28.02% | 0.004 |
|  | SII | 3.43% | -6.87% | 17.76% | 0.482 |
|  | SIRI | 5.94% | 0.09% | 17.06% | 0.048 |
|  | INFLA score | 13.61% | 4.08% | 36.06% | 0.006 |
| UC | Albumin | 13.82% | 5.86% | 50.63% | 0.010 |
|  | CRP | 8.51% | 3.38% | 36.38% | 0.016 |
|  | Lymphocyte | -0.66% | -4.79% | 1.98% | 0.482 |
|  | Platelet | -0.84% | -9.06% | 5.37% | 0.700 |
|  | Eosinophil | -0.09% | -3.05% | 2.29% | 0.854 |
|  | Monocyte | 0.62% | -1.02% | 4.20% | 0.326 |
|  | Neutrophil | 7.77% | 2.12% | 31.11% | 0.010 |
|  | WBC | 2.28% | 0.21% | 9.97% | 0.030 |
|  | LMR | -0.09% | -3.70% | 3.07% | 0.912 |
|  | NLR | 10.70% | 3.08% | 45.62% | 0.018 |
|  | PLR | 4.97% | -1.23% | 21.55% | 0.100 |
|  | CAR | 9.10% | 3.59% | 36.40% | 0.004 |
|  | SII | 10.13% | 3.37% | 47.90% | 0.008 |
|  | SIRI | 4.40% | 1.33% | 17.56% | 0.004 |
|  | INFLA score | 10.11% | 1.84% | 41.21% | 0.028 |
| IBD-U | Albumin | 1.09% | -4.51% | 8.03% | 0.666 |
|  | CRP | 0.82% | -5.85% | 7.41% | 0.760 |
|  | Lymphocyte | 0.01% | -3.46% | 4.01% | 0.978 |
|  | Platelet | 1.34% | -2.80% | 7.34% | 0.448 |
|  | Eosinophil | 2.10% | -2.25% | 9.02% | 0.312 |
|  | Monocyte | -0.01% | -2.75% | 2.49% | 0.990 |
|  | Neutrophil | 6.83% | 1.62% | 19.54% | 0.006 |
|  | WBC | 3.45% | 0.38% | 11.03% | 0.018 |
|  | LMR | 2.00% | -2.69% | 11.31% | 0.366 |
|  | NLR | -0.77% | -8.87% | 8.04% | 0.818 |
|  | PLR | -3.26% | -14.02% | 4.38% | 0.384 |
|  | CAR | 0.87% | -4.71% | 8.68% | 0.690 |
|  | SII | 1.09% | -7.58% | 10.43% | 0.774 |
|  | SIRI | 1.40% | -3.08% | 7.98% | 0.500 |
|  | INFLA score | 9.88% | 2.68% | 24.27% | 0.008 |
| **CVD Outcomes: Ischemic heart disease** | | | | | |
| **Exposure** | **Inflammation biomarker** | **Proportion mediation** | **95% Lower** | **95% Upper** | ***P*** |
| IBD-U | Albumin | 0.74% | -10.58% | 12.96% | 0.852 |
|  | CRP | 1.71% | -8.53% | 18.54% | 0.642 |
|  | Lymphocyte | 0.66% | -5.69% | 9.72% | 0.692 |
|  | Platelet | 5.35% | -0.40% | 28.26% | 0.068 |
|  | Eosinophil | 1.93% | -6.65% | 15.45% | 0.542 |
|  | Monocyte | -0.02% | -4.99% | 4.65% | 0.984 |
|  | Neutrophil | 8.60% | 0.56% | 37.92% | 0.038 |
|  | WBC | 3.89% | -0.36% | 19.55% | 0.072 |
|  | LMR | 4.46% | -6.40% | 25.10% | 0.294 |
|  | NLR | 2.54% | -8.55% | 23.54% | 0.624 |
|  | PLR | 1.30% | -12.30% | 17.82% | 0.814 |
|  | CAR | 1.62% | -8.37% | 19.82% | 0.612 |
|  | SII | 7.01% | -4.78% | 33.30% | 0.166 |
|  | SIRI | 3.07% | -3.58% | 18.16% | 0.312 |
|  | INFLA score | 14.38% | 2.51% | 65.43% | 0.030 |
| **CVD Outcomes: Cerebrovascular disease** | | | | | |
| **Exposure** | **Inflammation biomarker** | **Proportion mediation** | **95% Lower** | **95% Upper** | ***P*** |
| CD | Albumin | 2.58% | -21.40% | 29.97% | 0.672 |
|  | CRP | 10.20% | -56.19% | 114.18% | 0.090 |
|  | Lymphocyte | 1.05% | -12.54% | 26.07% | 0.744 |
|  | Platelet | -1.01% | -25.77% | 25.19% | 0.794 |
|  | Eosinophil | -0.04% | -7.41% | 3.72% | 0.906 |
|  | Monocyte | -0.18% | -7.87% | 4.25% | 0.710 |
|  | Neutrophil | -0.36% | -21.02% | 24.48% | 0.948 |
|  | WBC | -0.14% | -14.31% | 11.89% | 0.930 |
|  | LMR | 0.23% | -8.48% | 13.02% | 0.824 |
|  | NLR | 9.05% | -44.84% | 86.23% | 0.206 |
|  | PLR | 11.65% | -12.81% | 78.28% | 0.094 |
|  | CAR | 10.34% | -19.21% | 123.25% | 0.096 |
|  | SII | 14.09% | -28.83% | 89.32% | 0.104 |
|  | SIRI | 10.47% | -48.79% | 95.23% | 0.094 |
|  | INFLA score | -0.54% | -50.30% | 35.29% | 0.948 |
| **CVD Outcomes: Heart failure** | | | | | |
| **Exposure** | **Inflammation biomarker** | **Proportion mediation** | **95% Lower** | **95% Upper** | ***P*** |
| IBD | Albumin | 8.91% | 3.93% | 19.22% | <0.001 |
|  | CRP | 7.41% | 3.48% | 15.63% | <0.001 |
|  | Lymphocyte | 0.73% | -2.61% | 4.20% | 0.610 |
|  | Platelet | 1.19% | -2.90% | 5.92% | 0.506 |
|  | Eosinophil | 1.08% | -0.20% | 3.50% | 0.092 |
|  | Monocyte | 0.23% | -0.97% | 1.57% | 0.628 |
|  | Neutrophil | 4.45% | 0.37% | 11.99% | 0.036 |
|  | WBC | 1.34% | 0.00% | 3.73% | 0.050 |
|  | LMR | 3.32% | 0.44% | 10.37% | 0.022 |
|  | NLR | 7.22% | 2.40% | 17.11% | 0.002 |
|  | PLR | 3.99% | -0.15% | 10.14% | 0.060 |
|  | CAR | 7.61% | 3.80% | 15.48% | <0.001 |
|  | SII | 8.54% | 3.59% | 19.53% | 0.004 |
|  | SIRI | 3.76% | 1.86% | 8.14% | <0.001 |
|  | INFLA score | 10.49% | 3.18% | 24.75% | 0.004 |
| CD | Albumin | 8.74% | -8.51% | 57.19% | 0.164 |
|  | CRP | 15.73% | -115.63% | 165.01% | 0.088 |
|  | Lymphocyte | -4.79% | -36.55% | 2.19% | 0.076 |
|  | Platelet | -4.32% | -37.06% | 9.28% | 0.338 |
|  | Eosinophil | -0.01% | -4.85% | 4.00% | 0.974 |
|  | Monocyte | -0.35% | -7.27% | 2.74% | 0.584 |
|  | Neutrophil | 4.89% | -8.06% | 37.44% | 0.288 |
|  | WBC | 3.78% | -1.69% | 44.38% | 0.122 |
|  | LMR | -0.01% | -4.57% | 3.96% | 0.966 |
|  | NLR | 11.20% | -9.35% | 107.31% | 0.110 |
|  | PLR | -1.19% | -45.82% | 25.13% | 0.876 |
|  | CAR | 16.32% | -49.65% | 159.32% | 0.070 |
|  | SII | 7.12% | -20.30% | 49.79% | 0.402 |
|  | SIRI | 11.30% | 0.08% | 105.21% | 0.050 |
|  | INFLA score | 16.57% | -29.88% | 109.40% | 0.110 |
| IBD-U | Albumin | 4.59% | -1.37% | 15.51% | 0.124 |
|  | CRP | -1.36% | -10.84% | 6.49% | 0.680 |
|  | Lymphocyte | 1.12% | -2.92% | 8.00% | 0.492 |
|  | Platelet | -0.61% | -7.79% | 4.12% | 0.740 |
|  | Eosinophil | 0.81% | -5.02% | 7.86% | 0.720 |
|  | Monocyte | -0.01% | -2.35% | 2.17% | 0.972 |
|  | Neutrophil | 0.56% | -6.08% | 7.57% | 0.842 |
|  | WBC | 0.02% | -4.91% | 4.42% | 0.972 |
|  | LMR | 3.22% | -2.86% | 17.58% | 0.266 |
|  | NLR | -0.04% | -8.85% | 9.50% | 0.990 |
|  | PLR | -0.88% | -10.70% | 10.07% | 0.816 |
|  | CAR | -1.32% | -10.47% | 6.12% | 0.702 |
|  | SII | 0.48% | -9.19% | 11.29% | 0.896 |
|  | SIRI | 1.83% | -3.75% | 8.83% | 0.426 |
|  | INFLA score | 1.10% | -9.02% | 12.27% | 0.788 |
| **CVD Outcomes: Arrhythmias** | | | | | |
| **Exposure** | **Inflammation biomarker** | **Proportion mediation** | **95% Lower** | **95% Upper** | ***P*** |
| CD | Albumin | 1.41% | -8.32% | 11.89% | 0.724 |
|  | CRP | 5.52% | -0.69% | 21.56% | 0.080 |
|  | Lymphocyte | -2.93% | -11.97% | 0.34% | 0.090 |
|  | Platelet | -6.59% | -21.09% | -0.69% | 0.034 |
|  | Eosinophil | -0.04% | -2.56% | 1.71% | 0.882 |
|  | Monocyte | -0.32% | -3.62% | 0.88% | 0.516 |
|  | Neutrophil | 0.22% | -7.16% | 8.36% | 0.930 |
|  | WBC | 1.23% | -2.10% | 7.20% | 0.430 |
|  | LMR | 0.00% | -1.67% | 1.95% | 0.984 |
|  | NLR | 0.21% | -13.93% | 14.39% | 0.966 |
|  | PLR | -8.67% | -38.28% | 4.64% | 0.178 |
|  | CAR | 5.71% | -1.30% | 22.18% | 0.114 |
|  | SII | -5.76% | -29.98% | 9.39% | 0.438 |
|  | SIRI | 3.56% | -3.65% | 15.47% | 0.316 |
|  | INFLA score | 1.45% | -10.64% | 17.83% | 0.784 |
| UC | Albumin | 11.37% | 2.36% | 67.70% | 0.036 |
|  | CRP | 7.93% | 1.26% | 60.73% | 0.044 |
|  | Lymphocyte | 0.01% | -6.27% | 6.97% | 0.992 |
|  | Platelet | -4.97% | -35.43% | 3.47% | 0.134 |
|  | Eosinophil | -0.31% | -5.93% | 3.36% | 0.698 |
|  | Monocyte | 1.04% | -2.69% | 7.70% | 0.304 |
|  | Neutrophil | 6.66% | -2.56% | 34.09% | 0.092 |
|  | WBC | 1.66% | -1.62% | 11.32% | 0.184 |
|  | LMR | 3.45% | -1.48% | 30.21% | 0.136 |
|  | NLR | 12.79% | -12.55% | 75.92% | 0.054 |
|  | PLR | 4.89% | -9.77% | 39.49% | 0.218 |
|  | CAR | 8.41% | 1.36% | 46.88% | 0.046 |
|  | SII | 9.61% | -3.21% | 58.21% | 0.062 |
|  | SIRI | 5.03% | 1.09% | 31.88% | 0.034 |
|  | INFLA score | 4.69% | -14.63% | 38.07% | 0.390 |
| **CVD Outcomes: Atrial fibrillation** | | | | | |
| **Exposure** | **Inflammation biomarker** | **Proportion mediation** | **95% Lower** | **95% Upper** | ***P*** |
| CD | Albumin | 3.63% | -5.48% | 17.19% | 0.358 |
|  | CRP | 5.49% | -0.74% | 23.19% | 0.074 |
|  | Lymphocyte | -2.45% | -10.99% | 0.85% | 0.166 |
|  | Platelet | -9.65% | -29.81% | -2.71% | 0.006 |
|  | Eosinophil | -0.07% | -3.11% | 2.03% | 0.882 |
|  | Monocyte | -0.34% | -4.04% | 0.87% | 0.502 |
|  | Neutrophil | 0.18% | -7.71% | 8.66% | 0.956 |
|  | WBC | 1.19% | -2.32% | 7.67% | 0.460 |
|  | LMR | 0.01% | -1.98% | 2.55% | 0.960 |
|  | NLR | 1.14% | -12.78% | 15.84% | 0.802 |
|  | PLR | -9.27% | -41.73% | 4.80% | 0.178 |
|  | CAR | 5.68% | -1.38% | 23.13% | 0.110 |
|  | SII | -7.01% | -34.35% | 8.85% | 0.386 |
|  | SIRI | 4.44% | -2.47% | 17.56% | 0.194 |
|  | INFLA score | 1.16% | -11.79% | 18.74% | 0.824 |
| **CVD Outcomes: Other supraventricular arrhythmias** | | | | | |
| **Exposure** | **Inflammation biomarker** | **Proportion mediation** | **95% Lower** | **95% Upper** | ***P*** |
| IBD | Albumin | 3.22% | -7.07% | 19.21% | 0.388 |
|  | CRP | 0.08% | -13.38% | 13.67% | 0.988 |
|  | Lymphocyte | 4.22% | -5.01% | 23.48% | 0.272 |
|  | Platelet | -2.60% | -19.79% | 6.29% | 0.398 |
|  | Eosinophil | 0.22% | -3.87% | 4.71% | 0.856 |
|  | Monocyte | 0.10% | -1.18% | 2.02% | 0.702 |
|  | Neutrophil | 3.16% | -5.19% | 23.91% | 0.366 |
|  | WBC | 0.39% | -4.57% | 6.53% | 0.818 |
|  | LMR | 3.14% | -2.02% | 19.98% | 0.182 |
|  | NLR | 6.59% | -1.00% | 35.58% | 0.094 |
|  | PLR | 3.45% | -6.36% | 22.49% | 0.376 |
|  | CAR | 0.74% | -10.36% | 14.17% | 0.836 |
|  | SII | 6.93% | -3.53% | 34.45% | 0.118 |
|  | SIRI | 3.09% | 0.18% | 14.65% | 0.044 |
|  | INFLA score | 2.11% | -14.40% | 28.95% | 0.708 |
| **CVD Outcomes: Venous thromboembolism** | | | | | |
| **Exposure** | **Inflammation biomarker** | **Proportion mediation** | **95% Lower** | **95% Upper** | ***P*** |
| UC | Albumin | 7.88% | 3.06% | 19.36% | <0.001 |
|  | CRP | 3.62% | 0.58% | 10.56% | 0.018 |
|  | Lymphocyte | 3.95% | 0.03% | 14.90% | 0.046 |
|  | Platelet | 1.74% | -2.27% | 7.49% | 0.356 |
|  | Eosinophil | -0.70% | -3.59% | 0.63% | 0.258 |
|  | Monocyte | -0.10% | -1.79% | 0.81% | 0.742 |
|  | Neutrophil | 1.71% | -2.88% | 7.83% | 0.460 |
|  | WBC | -0.46% | -3.97% | 1.90% | 0.644 |
|  | LMR | -0.42% | -2.41% | 0.63% | 0.406 |
|  | NLR | 3.15% | -2.32% | 11.99% | 0.258 |
|  | PLR | 3.19% | -0.38% | 9.97% | 0.074 |
|  | CAR | 3.68% | 0.72% | 9.59% | 0.010 |
|  | SII | 3.75% | -1.00% | 13.20% | 0.144 |
|  | SIRI | 1.84% | -0.24% | 5.76% | 0.092 |
|  | INFLA score | 6.60% | 0.44% | 19.20% | 0.032 |
| IBD-U | Albumin | 7.32% | -0.68% | 32.06% | 0.074 |
|  | CRP | -2.92% | -23.58% | 9.37% | 0.556 |
|  | Lymphocyte | 0.48% | -6.31% | 9.27% | 0.784 |
|  | Platelet | -0.68% | -9.62% | 8.29% | 0.812 |
|  | Eosinophil | 1.28% | -7.24% | 13.13% | 0.672 |
|  | Monocyte | 0.00% | -4.10% | 4.43% | 0.996 |
|  | Neutrophil | 2.30% | -6.18% | 17.33% | 0.536 |
|  | WBC | 0.56% | -6.38% | 9.23% | 0.804 |
|  | LMR | -0.02% | -3.34% | 3.15% | 0.954 |
|  | NLR | -1.31% | -18.07% | 15.92% | 0.826 |
|  | PLR | -1.52% | -19.17% | 12.70% | 0.768 |
|  | CAR | -2.21% | -18.69% | 13.21% | 0.662 |
|  | SII | -2.61% | -25.54% | 13.37% | 0.702 |
|  | SIRI | -1.96% | -17.18% | 9.00% | 0.630 |
|  | INFLA score | 3.05% | -11.48% | 21.37% | 0.568 |
| **CVD Outcomes: Deep vein thrombosis** | | | | | |
| **Exposure** | **Inflammation biomarker** | **Proportion mediation** | **95% Lower** | **95% Upper** | ***P*** |
| UC | Albumin | 5.61% | 1.70% | 12.60% | 0.004 |
|  | CRP | 1.99% | -0.78% | 6.43% | 0.182 |
|  | Lymphocyte | 1.27% | -2.20% | 6.62% | 0.428 |
|  | Platelet | 0.47% | -3.43% | 4.34% | 0.746 |
|  | Eosinophil | 0.21% | -1.03% | 1.95% | 0.588 |
|  | Monocyte | 0.08% | -0.73% | 1.14% | 0.698 |
|  | Neutrophil | 2.40% | -1.38% | 7.51% | 0.244 |
|  | WBC | 0.37% | -1.46% | 2.44% | 0.608 |
|  | LMR | -0.24% | -1.96% | 0.95% | 0.638 |
|  | NLR | 1.77% | -3.44% | 7.87% | 0.454 |
|  | PLR | 1.73% | -1.92% | 6.19% | 0.304 |
|  | CAR | 2.00% | -0.74% | 5.80% | 0.168 |
|  | SII | 2.28% | -2.08% | 8.38% | 0.326 |
|  | SIRI | 1.52% | -0.01% | 3.95% | 0.052 |
|  | INFLA score | 4.28% | -1.39% | 12.29% | 0.138 |

Significance of mediating effects were determined by 1000 quasi-Bayesian approximation iterations.

CVD with less than 100 observed cases were not analyzed due to power issue, but still contributed to the analysis for corresponding main categories.

*CRP, C-reactive protein; LMR, lymphocyte to monocyte ratio; NLR, neutrophil to lymphocyte ratio; PLR, Platelet to lymphocyte ratio; CAR, C-reactive protein to albumin ratio; SII, Systemic immune-inflammatory index; SIRI, Systemic inflammation response index.*

**
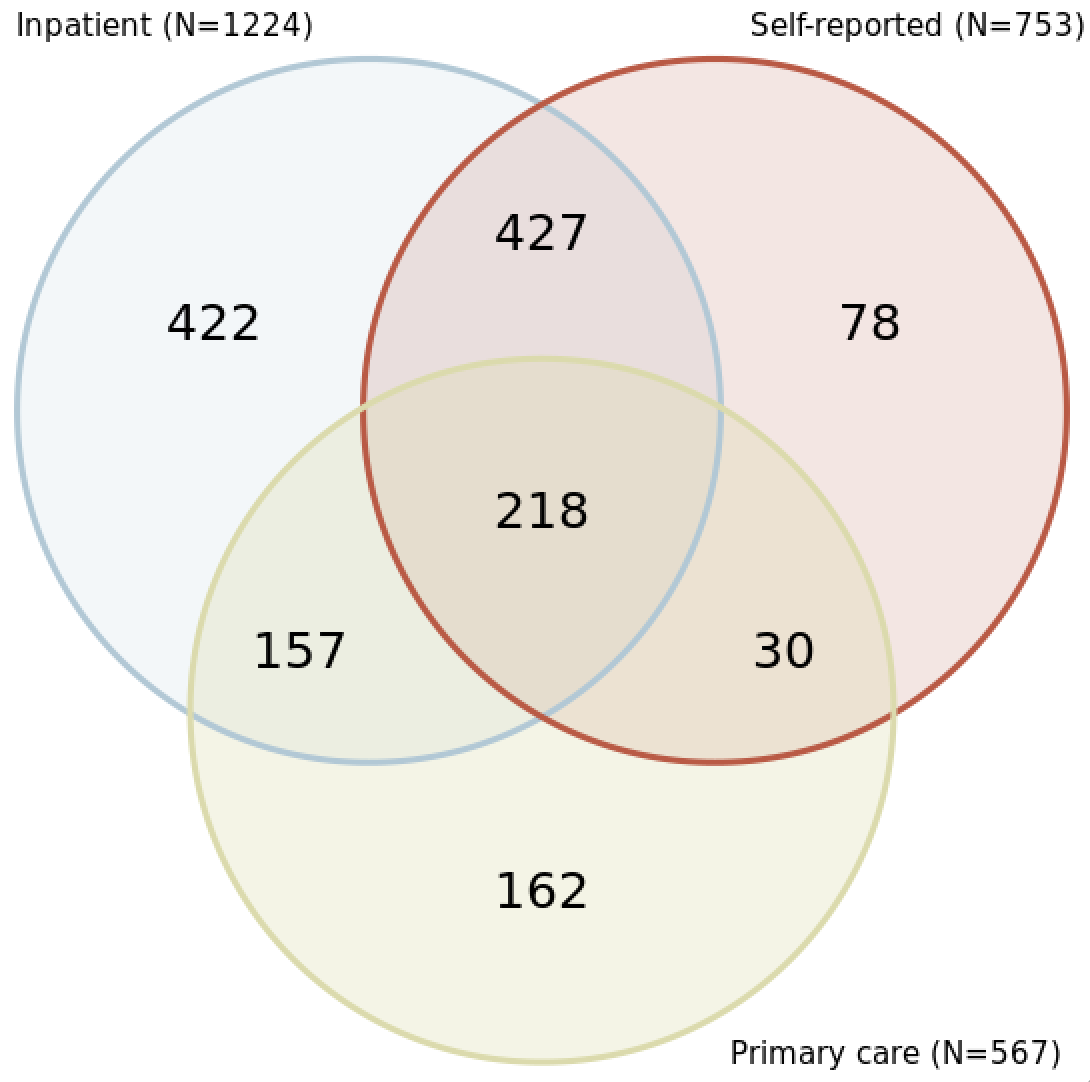
**

# Figure S1. Source of inflammatory bowel disease (IBD).

**
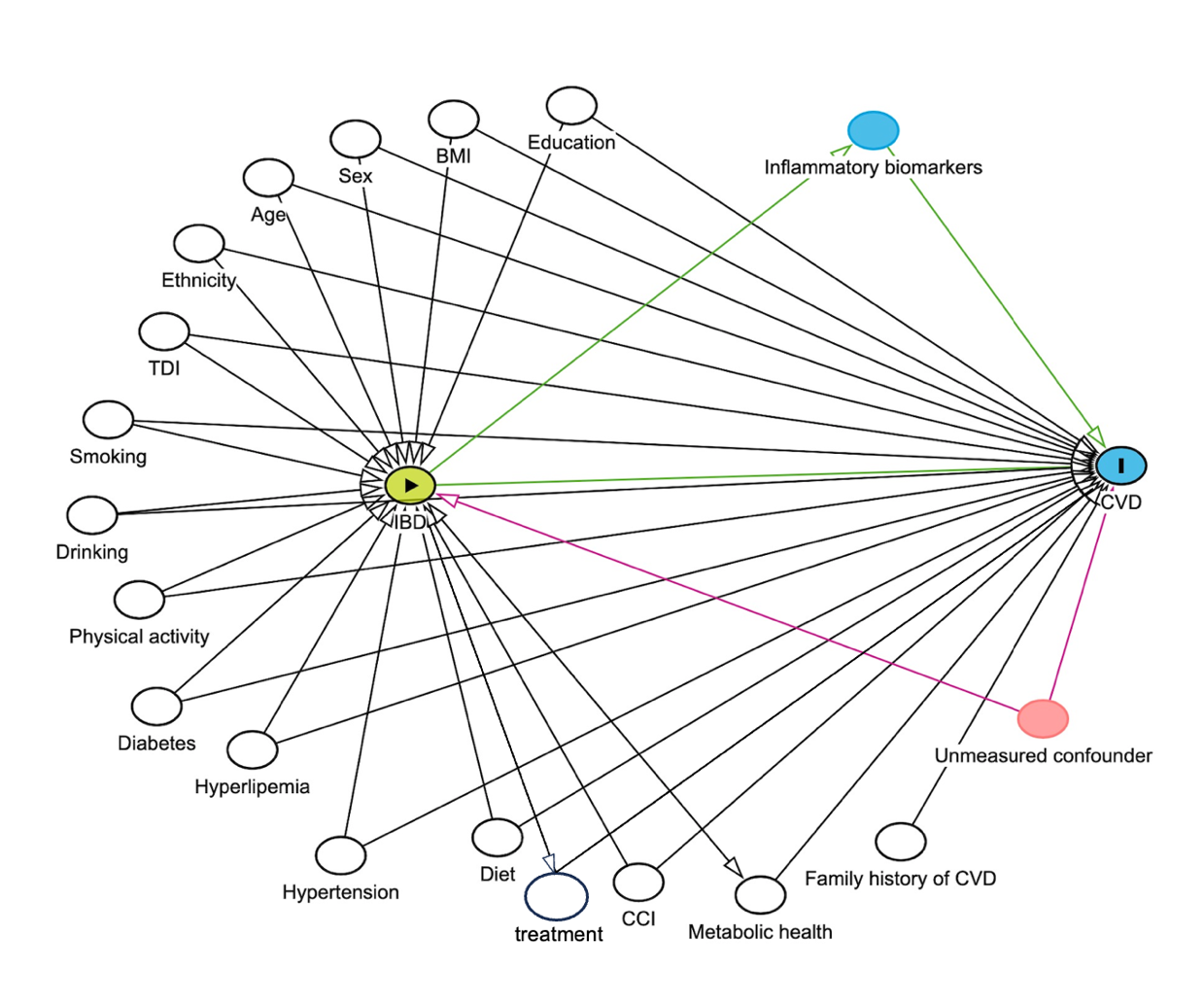
**

# Figure S2. Directed acyclic graph (DAG) of the potential causal relationship between exposure (IBD) and outcome (CVD) and other factors influencing the relationship.


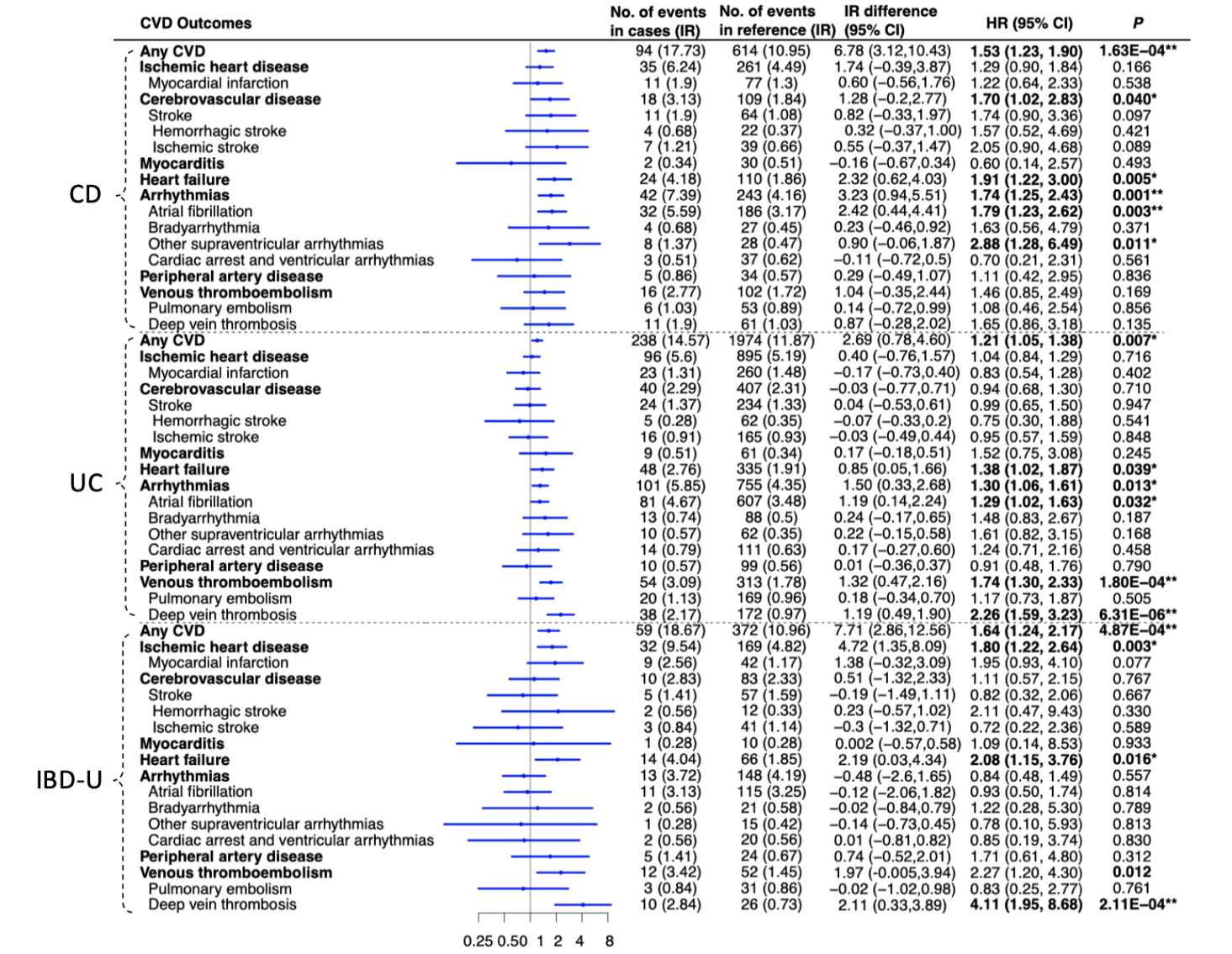


# Figure S3. Forest plot of associations between Crohn’s disease (CD), ulcerative colitis (UC), and IBD-unclassified (IBD-U) and cardiovascular disease (CVD). Incidence rate (IR) difference expressed as per 1,000 person-years. CI, confidence interval. The association with Bonferroni corrected *P* values<0.05 was labelled as **, while Bonferroni corrected *P*≥0.05 and *P* value<0.05 were labelled as *.


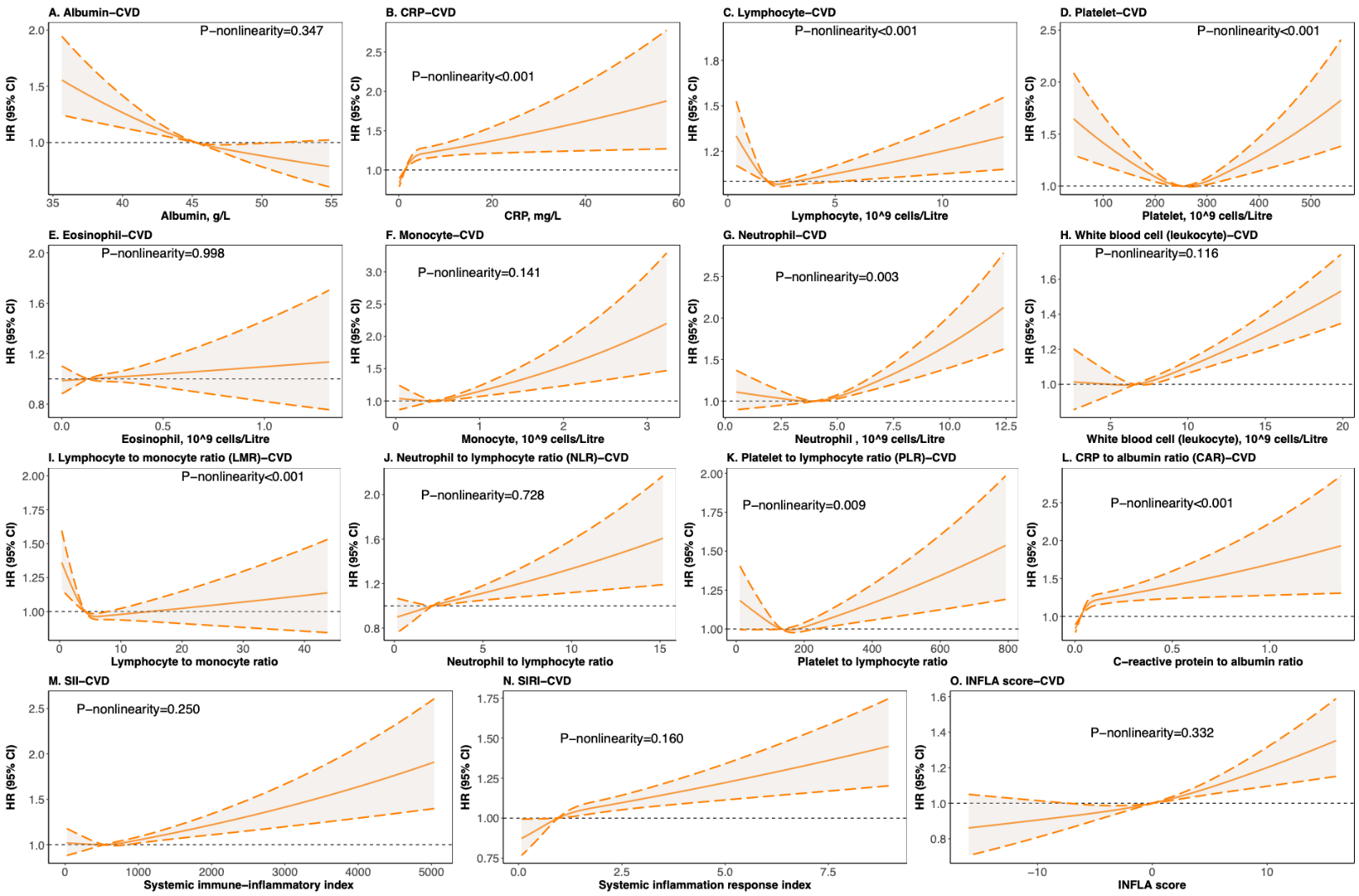


# Figure S4. Restricted cubic spline function (RCS) assessing the nonlinear association between inflammatory biomarkers and incident any cardiovascular disease (CVD). Median value was used as the reference value for the RCS curve.
